# *In Vivo* Clonal Analysis Reveals Random Monoallelic Expression in Lymphocytes That Traces Back to Hematopoietic Stem Cells

**DOI:** 10.3389/fcell.2022.827774

**Published:** 2022-08-08

**Authors:** Nadiya Kubasova, Clara F. Alves-Pereira, Saumya Gupta, Svetlana Vinogradova, Alexander Gimelbrant, Vasco M. Barreto

**Affiliations:** ^1^ Chronic Diseases Research Centre, Nova Medical School, CEDOC, Lisbon, Portugal; ^2^ Genetagus, Egas Moniz – Cooperativa de Ensino Superior, CRL, Monte de Caparica, Portugal; ^3^ Center of Cancer Systems Biology, Department of Cancer Biology, Dana-Farber Cancer Institute, Boston, MA, United States; ^4^ Department of Genetics, Harvard Medical School, Boston, MA, United States; ^5^ Broad Institute of MIT and Harvard, Cambridge, MA, United States; ^6^ Department of Genetics, Smurfit Institute of Genetics, Trinity College Dublin, University of Dublin, Dublin, Ireland; ^7^ UCIBIO, Departamento de Ciências da Vida, Faculdade de Ciências e Tecnologia, Universidade NOVA de Lisboa, Costa da Caparica, Portugal

**Keywords:** allele-specific expression, random monoallelic expression (RME), allelic imbalance (AI), epigenetics, clonal analysis, hematopoietic stem cell (HSC), X-chromosome inactivation (XCI), RNA-seq

## Abstract

Evaluating the epigenetic landscape in the stem cell compartment at the single-cell level is essential to assess the cells’ heterogeneity and predict their fate. Here, using a genome-wide transcriptomics approach *in vivo*, we evaluated the allelic expression imbalance in the progeny of single hematopoietic cells (HSCs) as a read-out of epigenetic marking. After 4 months of extensive proliferation and differentiation, we found that X-chromosome inactivation (XCI) is tightly maintained in all single-HSC derived hematopoietic cells. In contrast, the vast majority of the autosomal genes did not show clonal patterns of random monoallelic expression (RME). However, a persistent allele-specific autosomal transcription in HSCs and their progeny was found in a rare number of cases, none of which has been previously reported. These data show that: 1) XCI and RME in the autosomal chromosomes are driven by different mechanisms; 2) the previously reported high frequency of genes under RME in clones expanded *in vitro* (up to 15%) is not found in clones undergoing multiple differentiation steps *in vivo*; 3) prior to differentiation, HSCs have stable patterns of autosomal RME. We propose that most RME patterns in autosomal chromosomes are erased and established *de novo* during cell lineage differentiation.

## Introduction

One of the most remarkable features of multicellular organisms is the diversity of cellular phenotypes within each body. Isogenic cells display distinct phenotypes due to different epigenetic features or chromatin states that contribute to specific gene expression programs. Technical progress in next-generation sequencing (NGS) methods has produced a wealth of data on transcriptomics and genome-wide chromatin states of different lineages and stages within each lineage. However, distinguishing stable and reversible modes of gene regulation remains a challenge (van der Veeken et al., 2019). Likewise, the epigenetic and functional inter-clonal diversity within cell lineages has been difficult to capture. One proxy for approaching these questions is to explore the allelic differences in gene expression.

Diploid eukaryotic organisms inherit one allele from each parent and, in most cases, the two alleles of each gene are expressed at the same time and roughly similar levels in each cell. Exceptions to this biallelic expression pattern arise from asymmetries between the two alleles, leading to unequal expression that can be quantified as an “allelic imbalance” (AI) ranging from 0 to 1, with 0.5 corresponding to the balanced biallelic expression. Imbalance in allelic expression may have a genetic basis due to inherited differences in each allele’s *cis*-regulatory regions or acquired somatic DNA modifications or, alternatively, be caused by allele-specific epigenetic differences accumulated by the somatic cell. Parent-of-origin genomic imprinting (Reik and Walter, 2001) and X-chromosome inactivation (XCI) (Disteche and Berletch, 2015), the most well-studied examples of allelic expression imbalance due to epigenetic differences, cannot shed light on inter-clonal lineage diversity; in the former process, all somatic cells from the organism are virtually identical concerning the genomic imprint; in the latter, only two different cell populations emerge in females (differing in which X-chromosome was inactivated). Potentially more useful are the random epigenetic-based allelic expression imbalances that have been identified in autosomal genes at frequencies ranging from 2% to up to 15% of all expressed genes (Gimelbrant et al., 2007; Jeffries et al., 2012; Zwemer et al., 2012; Eckersley-Maslin et al., 2014; Gendrel et al., 2014). Some cells may express mostly or exclusively (monoallelically) one allele of these autosomal genes, whereas others express mostly or exclusively the other allele, a phenomenon known as random monoallelic expression (RME). These imbalances in heterozygous organisms establish clones within each cell lineage with structural and functional differences, and the population of clones, although emerging from isogenic cells, is said to be phenotypically diverse. The most spectacular and extensively studied examples of phenotypic diversity within initially isogenic cell populations due to RME are the antigen and olfactory receptor genes (Vettermann and Schlissel, 2010; Monahan and Lomvardas, 2015). However, it remains to be addressed if the concept applies broadly at the functional level to more genes (Gimelbrant et al., 2007), what is the real potential for clonal diversity based on the combinations of genes with distinct allelic expression levels, when these patterns are first established, how stable they are, and what parallels can be drawn between XCI and the RME of autosomal genes.

The studies reporting measurable frequencies of autosomal genes with random allelic expression imbalances were mainly performed in collections of clones expanded *in vitro*. In most cases, these clones were grown without undergoing differentiation or under limited differentiation. Building upon previous work (Alves-Pereira et al., 2014), here we report an allele-specific genome-wide transcriptome analysis of B and T cell populations emerging *in vivo* from a single hematopoietic stem cell (HSC). It is known that cells undergoing differentiation from embryonic stem cells acquire patterns of RME (Gendrel et al., 2014; Marion-Poll et al., 2021). HSCs are specialized cells and the heterogeneous repopulation phenotypes observed in single-HSC reconstitution studies possibly reflect epigenetic differences within the HSC pool (Sieburg et al., 2006; Dykstra et al., 2007; Benveniste et al., 2010; Morita et al., 2010; Yu et al., 2016). Thus, it is assumed that HSCs have genes under RME. Our aim was to evaluate whether regions in the autosomal chromosomes can keep stable expression patterns after extensive differentiation from a specialized cell type. This is the first report of a genome-wide transcriptomic analysis with allele-specific resolution of clones that differentiated and proliferated extensively *in vivo*.

## Results

### A Single HSC Gives Rise to Myeloid and Lymphoid Cells in the Blood

**FIGURE 1:**
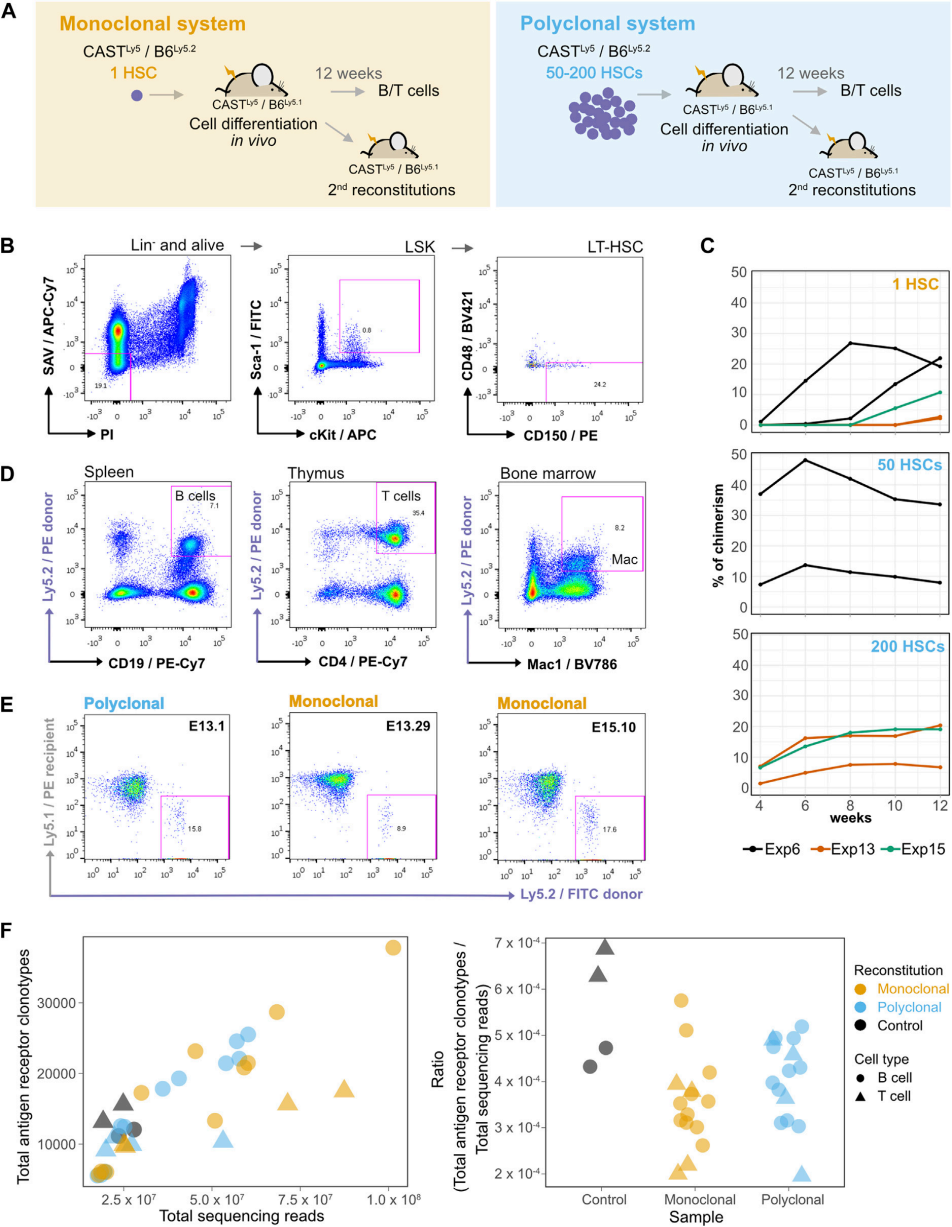
A single hematopoietic stem cell (HSC) gives rise to myeloid and lymphoid cells in the blood with long-term reconstitution. **(A)** Establishment of monoclonal and polyclonal hematopoietic systems *in vivo*. A single HSC or 50–200 HSCs were injected into sub-lethally irradiated recipient mice to generate a monoclonal or a polyclonal hematopoietic system, respectively. Different donor mice were used in each experiment. Both donor and recipient animals were the F1 progeny of CAST × B6 crosses, but the recipient and donor cells could be distinguished by the presence of a polymorphism in the pan-leukocyte antigen Ly5 [donor animals: F1(CAST^Ly5/Ly5^ × B6^Ly5.2/Ly5.2^), recipient animals: F1(CAST^Ly5/Ly5^ × B6^Ly5.2/Ly5.1^)]. Secondary reconstitutions and isolation of B/T cell populations were performed after 12 weeks of cell differentiation *in vivo*. **(B)** Long-term HSC (LT-HSC) isolation. The bone marrow cells of an F1 CAST^Ly5/Ly5^ × B6^Ly5.2/Ly5.2^ mouse were stained with a cocktail of biotin-conjugated antibodies for surface markers of lineage-committed cells (anti-B220, anti-CD19, anti-Mac1, anti-Ter119, anti-Gr1, and anti-CD3), and subsequently, lineage-marked cells were depleted using MACS Streptavidin MicroBeads. After depletion, cells were stained with fluorophore-conjugated antibodies: APC-conjugated anti-c-Kit, FITC-conjugated anti-Sca-1, BV421-conjugated anti-CD48, PE-conjugated anti-CD150, Streptavidin/APC-Cy7 (SAV/APC-Cy7), and PI, and sorted on a FACSAriaIII. The cells were gated for PI^−^/APC-Cy7^−^ to exclude dead cells and any remaining lineage-positive cells, then for c-Kit^+^/Sca-1^+^ to obtain Lin^−^Sca^+^c-Kit^+^ (LSK) cells, and finally gated for CD48^−^/CD150^+^ to obtain LT-HSCs. **(C)** Evolution of donor-derived cell population percentages over time in the peripheral blood of the recipient animals. After blood collection, red cells were lysed, and the cells were then stained with anti-Ly5.2 and analyzed on a FACSCanto or FACScan instrument. **(D)** A single donor HSC differentiates into lymphoid and myeloid hematopoietic populations *in vivo*. Cells from different hematopoietic organs of recipient animals were isolated, stained, gated on PI^−^, FITC anti-Ly5.1^+^, and PE anti-Ly5.2^−^, and identified as splenic B cells (PE-Cy7 anti-CD19^+^), CD4 thymocytes (PE-Cy7 anti-CD4^+^), or bone marrow macrophages (BV786 anti-Mac1^+^). **(E)** A single donor HSC repopulates secondary recipients. Representative plots of secondary reconstitutions 4 weeks post-reconstitution with bone marrow cells isolated from polyclonal and monoclonal primary reconstituted animals. Blood samples of secondary reconstituted mice were lysed for red cells, stained with FITC-conjugated anti-Ly5.2 for donor cells and PE-conjugated anti-Ly5.1 for recipient cells, and analyzed using FACSCanto. **(F)** VDJ clonotypes in different populations of donor-HSC-derived B and T cells expanded *in vivo,* and in the control animal. On the left panel, the numbers of sequenced reads (*x-axis*) were plotted against the number of unique VDJ rearrangements (“clonotypes”) identified with the MiXCR software tool on each sample (*y-axis*). The right panel shows the number of antigen clonotypes normalized by the total number of reads.

This work’s main goal is to study stable transcriptional states using transcriptomics with allelic resolution in a clonal system recreated *in vivo*. For this purpose, we introduced single HSCs from a donor female mouse into sub-lethally irradiated recipient females. These mice carried the Ly5.1 or Ly5.2 pan-leukocyte markers to distinguish recipient and donor cells, respectively (Supplementary Figure S1). The donor female F1 mice obtained by crossing C57BL/6J (B6) females with CAST/EiJ (CAST) males are characterized by high heterozygosity across the genome (Frazer et al., 2007), i.e., about 1 SNP per 80 bp of non-repetitive genome sequence, on average, therefore enabling allele-specific analysis. The transplanted cell was left to expand and differentiate *in vivo*, producing clonal multilineage cell populations derived from a single HSC. In parallel, 50 or 200 HSCs were also transplanted per animal to generate oligoclonal or polyclonal control populations (Figure 1A).

The HSC population is heterogeneous, and several protocols based on flow cytometry were developed to distinguish between long-term HSCs (LT-HSCs) and short-term HSCs (ST-HSCs) (Mayle et al., 2013). We eventually used the CD150^+^ and CD48^−^ signaling lymphocyte activation molecule family markers on lineage negative and Sca-1^+^/c-Kit^+^ (LSK) cells isolated from the bone marrow of donor mice (Kiel et al., 2005) to single-sort the LT-HSC population (Figure 1B). HSCs were introduced by intravenous retro-orbital injection into recipient mice. The presence of donor cells was evaluated over 12 weeks by identifying the Ly5.2^+^ cells in the blood of recipient mice (Figure 1C). From 16 experiments, 12 weeks after injection, we were able to reconstitute with a single HSC 7.7% (35/453) of recipient mice with a percentage of blood chimerism in the 1%–44% range, whereas for mice injected with 50 or 200 HSCs, on average 76.9% (30/39) were reconstituted and the blood chimerism was in the 2%–88% range (Supplementary Figure S2; Supplementary Table S1). This single-cell reconstitution efficiency is in the range of what has been described (Smith et al., 1991; Osawa et al., 1996; Wagers et al., 2002; Boyer et al., 2019).

Twelve weeks after injection, the animals with chimerism were sacrificed to isolate HSC-derived splenic donor B cells (CD19^+^IgM^+^), donor thymocytes (CD4^+^CD8^+^), and myeloid cell populations (Mac-1^+^) from monoclonal and polyclonal animals (Supplementary Figure S3; Figure 1D). We used bone marrow cells to produce secondary reconstitutions (Figure 1E), showing that these CD150^+^/CD48^−^ HSCs originate long-term and multilineage reconstitutions. RNA isolation and whole-transcriptome sequencing were performed for the HSC-derived B and T cell samples from the reconstituted animals. B and T cells from an unmanipulated donor female were used as additional non-clonal controls.

To gain quantitative insight into reconstitution dynamics in the lymphoid lineage from the single-HSC and control reconstituted animals, we used MiXCR-3.0.12 (Bolotin et al., 2017; Bolotin et al., 2015) to detect the antigen receptor V(D)J rearrangement clonotypes of sorted B and T cell samples. We observed roughly the same number of rearrangements in the single-HSC reconstitution samples, the samples produced from 50 to 200 HSCs, and the control samples, suggesting that there is a substantial cellular expansion in the single-HSC derived hematopoietic system before V(D)J rearrangement, which first occurs in pro-B and pro-T cells (Figure 1F). Thus, a single HSC gives rise to long-term reconstitutions, produces myeloid and lymphoid lineages in the recipient animal, can reconstitute a secondary recipient animal, and generates a repertoire of V(D)J clonotypes similar to those of the polyclonal and non-clonal controls. Taken together, the data mean that the cells used in the reconstitutions meet the definition of HSC (Kiel et al., 2005; Dykstra et al., 2007; Wilkinson et al., 2020) and that the clonal complexity in lymphoid populations derived from a single HSC is representative of the clonal complexity found in non-manipulated hematopoietic systems.

### Single-HSC Reconstitutions Produce Clonal Hematopoietic Systems

**FIGURE 2:**
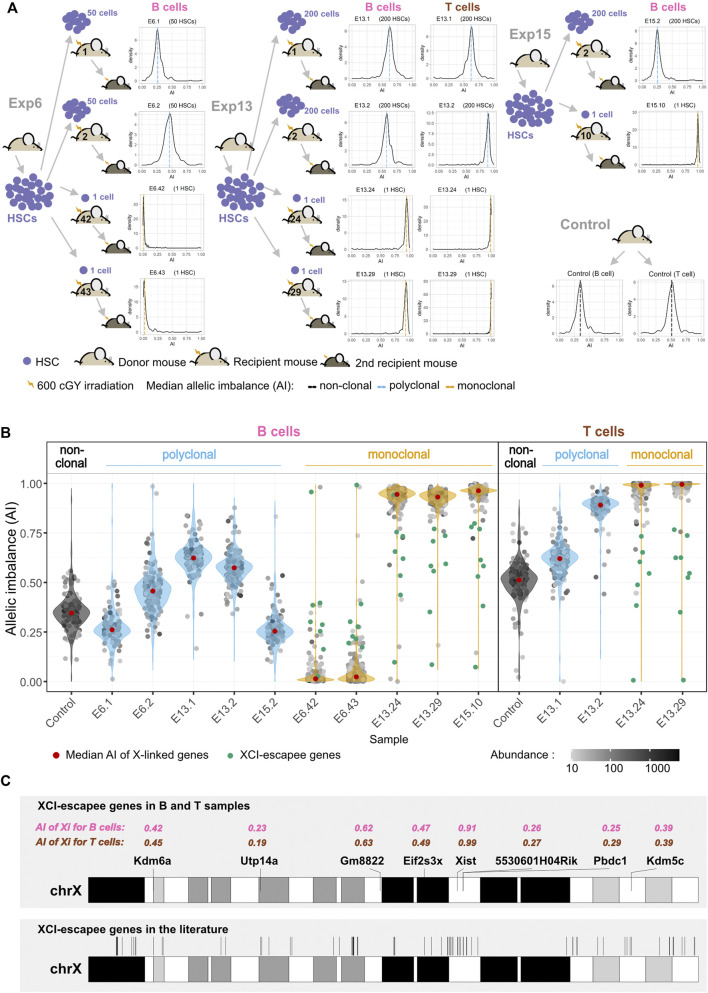
Single-HSC reconstitutions produce clonal hematopoietic systems. **(A)** Schematic representation of single and multiple HSC reconstitutions that originated the samples used for RNA-seq in this study (experiments E6, E13, and E15). In each experiment, HSCs isolated from one donor mouse F1(CAST^Ly5/Ly5^ × B6^Ly5.2/Ly5.2^) were injected into multiple sub-lethally irradiated recipient animals F1(CAST^Ly5/Ly5^ × B6^Ly5.2/Ly5.1^). Different donors were used for each experiment. All animals showed long-term reconstitutions, and both monoclonal and polyclonal cells from primary repopulated animals reconstituted a secondary recipient (see representative cytometry profiles in Figure 1). The density plots represent the allelic ratios of X chromosome-linked genes for each sample, as measured by RNA-seq. **(B)** AI of X-linked genes and X-Chromosome Inactivation (XCI) escapee genes. Violin plots superimposing dot plots of X-linked genes allelic ratios per clonal/polyclonal B/T cell sample. For grey dots, the opacity reflects the relative abundance in trimmed mean of M (TMM)-normalized counts. Genes significantly escaping XCI (green dots) are the ones for which the AI value is significantly above (or below) the median AI value of all genes plus (or minus) 0.1 when the CAST (or B6) X chromosome is expressed (more details are given in the *Materials and Methods* section). **(C)** X chromosome ideogram annotating the location of XCI escapee genes confirmed in this study for B and T cells (upper ideogram) and in the literature (lower ideogram). The AI of XCI escapee genes are denoted in pink (for B cell samples) and brown (for T cell samples).

For each experiment, HSCs isolated from one donor mouse [F1(CAST^Ly5/Ly5^ × B6^Ly5.2/Ly5.2^)] were injected into multiple sub-lethally irradiated recipient animals [F1(CAST^Ly5/Ly5^ × B6^Ly5.2/Ly5.1^)] and allowed to expand *in vivo*. A different donor was used in each experiment. Donor-HSC-derived B cells from polyclonal and monoclonal expansions of three different experiments (E6, E13, and E15) were FACS-sorted and cDNA was sequenced (RNA-seq); for experiment 13, donor-HSC-derived T cells were additionally sorted and sequenced. B and T cells from one unmanipulated animal of the same genotype as donors were used as non-clonal control populations (Figure 2A). We took advantage of XCI to internally confirm the monoclonality vs*.* oligo or polyclonality of the reconstitutions. A single HSC produces not only multilineage long-term reconstitutions but also hematopoietic cell populations that are clonal. In a hematopoietic system derived from a single female HSC, all cells must have inactivated the same X chromosome, producing a complete skewing of the maternal and paternal X-linked AI [maternal allele/(maternal + paternal alleles)], which will be equal to 1 or 0. Given that the *Xist* non-coding RNA is only expressed from the inactivated X chromosome, we first performed Sanger sequencing on *Xist* cDNA, focusing on two strain-specific SNPs. As expected, the chromatograms show two overlapping peaks for the control animals, whereas only one peak was observed in the chromatogram of single-HSC reconstituted animals (Supplementary Figure S4). We then deepened this analysis by calculating the AI for the X-linked genes from the NGS transcriptomics data. The AI value for the B cells of the unmanipulated control is in agreement with that reported for the same F1 mice (Chadwick and Willard, 2005); the value is below 0.5 due to polymorphisms in the *Xist locus*. T cells from the same animal show a much higher AI value (a bias that is also observed for the E13.2 reconstituted animal), probably because the cells expressing the B6 X chromosome have a slight advantage in the T cell lineage compared to the B lineage. The AI values for the polyclonal B cells fluctuate around the AI value of the unmanipulated mouse because, compared to this animal, much fewer cells are contributing to the hematopoietic system in the polyclonal reconstituted controls. We do not have a precise way of estimating how many HSCs per animal engrafted and produced a lineage, but based on the number of injected cells and the probability of HSC reconstitution for the individual HSC reconstitutions, that number is probably 5–20 HSCs per animal. As expected, in the single-HSC reconstituted mice the median AI value for X-linked genes is close to one (0.96 ± 0.03), namely in E13.24, E13.29, and E15.10, or zero (0.02 ± 0.01), as in E6.42 and E6.43 (medians of AI value for each sample as a red dot inside each violin plot of Figure 2B). Intriguingly, in samples from some single-HSC reconstituted animals, notably E13.24 and E13.29, for B cells, the median AI value is close to, but distinctly below, one. Three scenarios were considered to explain this puzzling observation: 1) more than one HSC was erroneously injected in these mice; 2) XCI is leaky in the sorted lymphocytes, given that inactivated X of mature naïve T and B cells has been reported to lack the typical heterochromatic modifications (Wang et al., 2016); 3) contaminating recipient (polyclonal) cells were present in the sorting cells. To sort out these hypotheses, we quantified the Ly5.1 and Ly5.2 SNPs in the NGS data. Half of the samples (*n* = 8) had around 1% of contaminating recipient cells; six samples had contaminating cells in the 2.5%–5% range, and the B cell samples of animals E13.24 and E13.29 (E13.24_B and E13.29_B) had contaminating cells in the 5%–10% range (Supplementary Figure S5). Since these two samples are precisely the ones with the most noticeable median AI deviation from 1, and T cell samples of the same animals do not show the same pattern, we conclude that the injections were indeed performed with single HSCs and that, at the assay’s resolution level, the data do not support the hypothesis that XCI in lymphocytes is leaky. Thus, the dataset is composed of monoclonal samples with a moderate, low or extremely low frequency of contaminating cells, and oligoclonal or polyclonal control samples.

Additionally, we searched for genes with nonrandom allelic biases in B or T samples, using stringent criteria (robust expression in all samples within a tissue and a 0.15 threshold). In the absence of reciprocal crosses, we cannot establish if these biases are caused by epigenetics (genomic imprinting) or genetics (differences in the promoter and other regulatory regions between the B6 and CAST alleles). We then compared our list of genes with nonrandom allelic biases with a list of imprinted genes from other studies (Supplementary Table S2; Supplementary Figure S6). Within the imprinted genes listed in the supplementary data of Tucci et al., 2019 and from the geneimprint database (https://www.geneimprint.com/), 55 were detected as robustly expressed [trimmed mean of M (TMM)-normalized counts > 10] in our B cell samples, and 62 genes in T cells. From these, only *Zrsr1* and *Igf2r* showed nonrandom allelic bias in B cells, and in addition to them, also *Airn* in T cells.

### Murine X-Linked Escapees Identified by Single-HSC Reconstitutions

Genes expressed from both the active and inactive X chromosomes are known as XCI escapees. In mice, XCI escapees have been studied using three systems: 1) single-cell RNA-seq (Borensztein et al., 2017; Chen et al., 2016); 2) heterozygous female mice knocked out for specific X-linked genes, such as *Xist* or *Hprt* (Berletch et al., 2015; Yang et al., 2010) or heterozygous female mice for an X-linked gene linked to a reporter (Wu et al., 2014); 3) and clonal female F1 hybrid cell lines (Calabrese et al., 2012; Li et al., 2012; Splinter et al., 2011). We sought to determine whether single-HSC reconstitution could be an additional strategy to identify hematopoietic lineage-specific X escapees. X-linked genes with expression from the Xi (inactive X chromosome) of at least 10% of total expression (Carrel and Willard, 2005) were identified taking into account the recipient cell contamination in each monoclonal sample (Figure 2B; see *Materials and Methods*). We identified a total of eight escapees, which were escapees both in B and T samples: *5530601H04Rik*, *Eif2s3x*, *Gm8822*, *Kdm5c*, *Kdm6a*, *Pbdc1*, *Utp14a,* and *Xist* (Supplementary Figure S7). These genes were plotted along the X chromosome and, as verified before (Li et al., 2012), they are not clustered (Figure 2C). Considering the literature, 117 genes have been described as XCI escapees in different mouse tissues and cell lines (Yang et al., 2010; Li et al., 2012; Wu et al., 2014; Berletch et al., 2015). Some of these genes were excluded from our analysis for lack of expression (36 genes), insufficient number of SNPs to measure AI (two genes), or for not being listed in the annotation reference used in this work (one gene). Overall, we could detect allelic expression for 78 genes known to escape XCI in these studies. Seven of the eight escapees identified in our B and T samples belong to this group; the only exception is *Gm8822*, an XCI pseudogene escapee in our dataset that was not the subject of investigation in other studies. Interestingly, 71 of the known escapee genes do not escape XCI in lymphocytes, which is consistent with the notion of tissue-specific XCI (Supplementary Table S3). Overall, we show that single-HSC transfer is an effective method to study lineage-specific XCI in blood cells.

### The Vast Majority of the Mitotically Stable Allelic Biases in Lymphocytes are not Established During the HSC Stage

**FIGURE 3:**
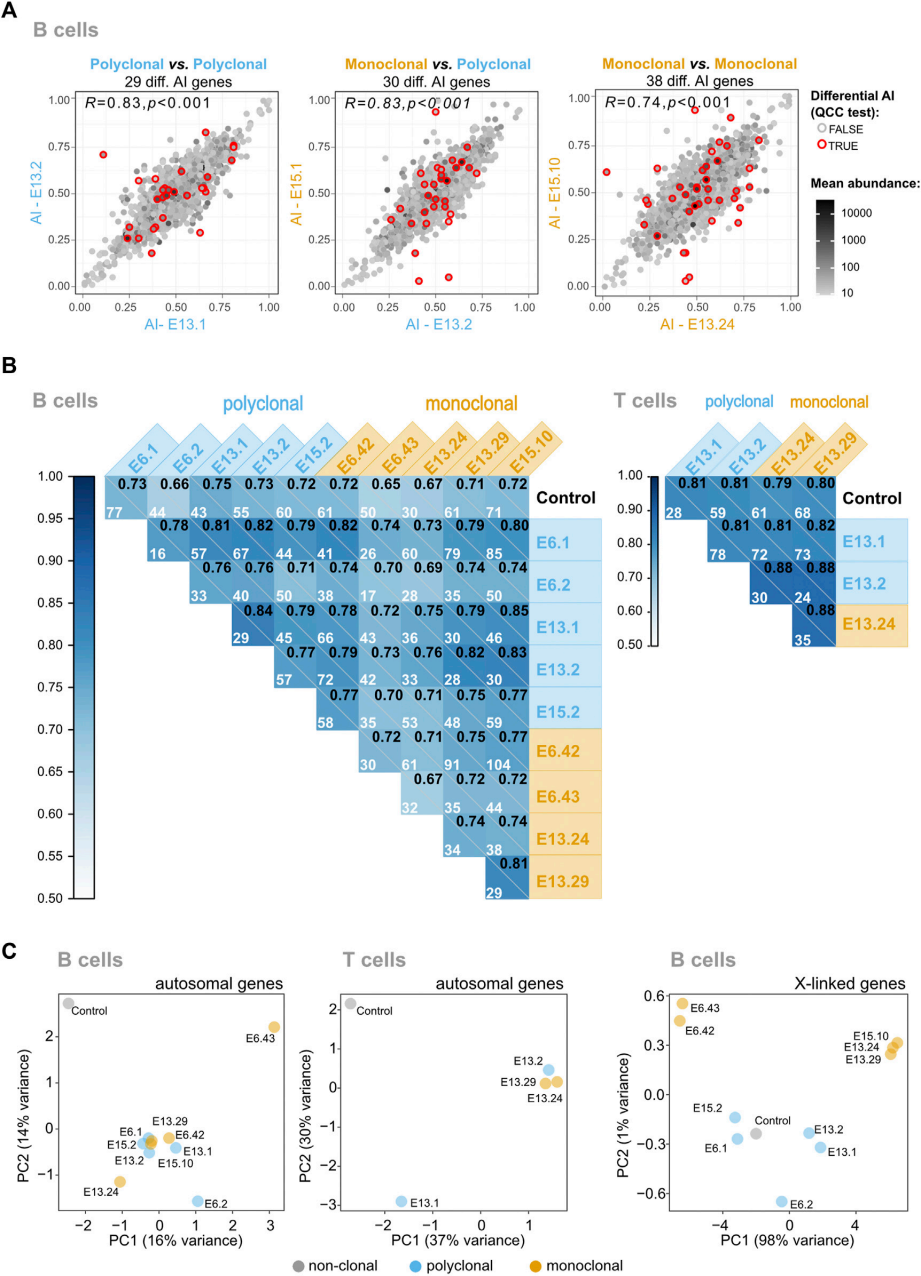
The vast majority of mitotically stable allelic biases of the hematopoietic system are not established during the HSC stage. **(A)** Representative plots of pairwise AI comparisons (monoclonal vs. polyclonal samples, polyclonal vs. polyclonal samples; and monoclonal vs. monoclonal samples). Red circles signal the genes for which differential AI remained statistically significant after quality control constant (QCC) correction, and the total number of these genes per comparison is shown above each plot. The Pearson’s coefficient correlation for all AI pairwise comparisons is also shown, in the upper left corner of each dot plot. A grayscale coloring the dots represents the mean expression between the two samples, calculated from each sample’s TMM-normalized counts. **(B)** Correlograms for B and T samples. Pearson’s coefficient correlation of AI for all pairwise comparisons between samples. Within each square, the Pearson’s coefficient is represented in the upper right corner, and the number of genes with a significant differential AI in each pairwise comparison after applying QCC correction on the binomial test is also shown. **(C)** Visualization of high-dimensional data of autosomal AI in a low-dimensional space using Principal Component Analysis suggests that the monoclonal animals have more variable AI values because of the slightly higher scattering compared to the polyclonal animals, but fails to reveal major differences between the two groups. As a control to show the impact of the AI values in the clustering of the samples in the low-dimensional space, the data from the X-linked genes of the monoclonals were added; as expected, these samples cluster according to the X chromosome (CAST or B6) that is expressed.

To test the genome for the presence of autosomal regions in B and T cells with stable monoallelic patterns of expression reminiscent of XCI (able to persist even after an extensive program of differentiation), we generated pairwise AI comparisons of monoclonal vs. polyclonal samples, polyclonal vs*.* polyclonal samples; and monoclonal vs*.* monoclonal samples (Figure 3A; Supplementary Figure S8). In the same way as for XCI, allele-specific expression for autosomal genes can be quantified as a fraction of one allele relative to the sum of both alleles: a/(a + A). This value of AI thus ranges from AI = 0 for the exclusive expression of allele A to AI = 1 for the exclusive expression of allele a, with AI = 0.5 for an equal expression of both alleles. A comparison of identical samples should align all genes over the diagonal; deviations from the diagonal indicate differences in AI between the samples for a given gene. We calculated the Pearson’s coefficient correlation of AI for all pairwise comparisons between samples and the number of genes with a significant differential AI in each pairwise comparison after applying quality control constant (QCC) correction on the binomial test (Figure 3B). If the samples from the monoclonal mice kept epigenetic states in autosomal regions in a clone-specific manner, then the correlations involving at least one monoclonal sample would be lower than the correlations found for the comparisons between controls. This was not observed. Likewise, the number of genes displaying significant differences for each pairwise comparison varies from 16 to 104 in B cells (Figure 3B) but the comparisons between the monoclonals do not stand out as the ones with more significant genes. Finally, Principal Component Analysis or PCA (an algorithm for high-dimensional data visualization in a low-dimensional space) of the AI for autosomal genes would have revealed a cluster of control samples and, if each clonal line kept distinct epigenetic states, the monoclonal samples would display a more scattered distribution (Figure 3C). The high-dimensional data analysis suggests a slightly higher scattering of the monoclonal sample AI values compared to the polyclonal samples, but it is difficult to translate these patterns into quantitative insights. We conclude that the regions in the autosomal chromosomes behaving like the X chromosomes in terms of the stable transcriptional states may not exist or represent only a small proportion of the genome that cannot be detected using these two analyses.

### Stable Transcriptional States of HSC-origin Persist in the Differentiated B Cells for a Small Number of Genes

**FIGURE 4:**
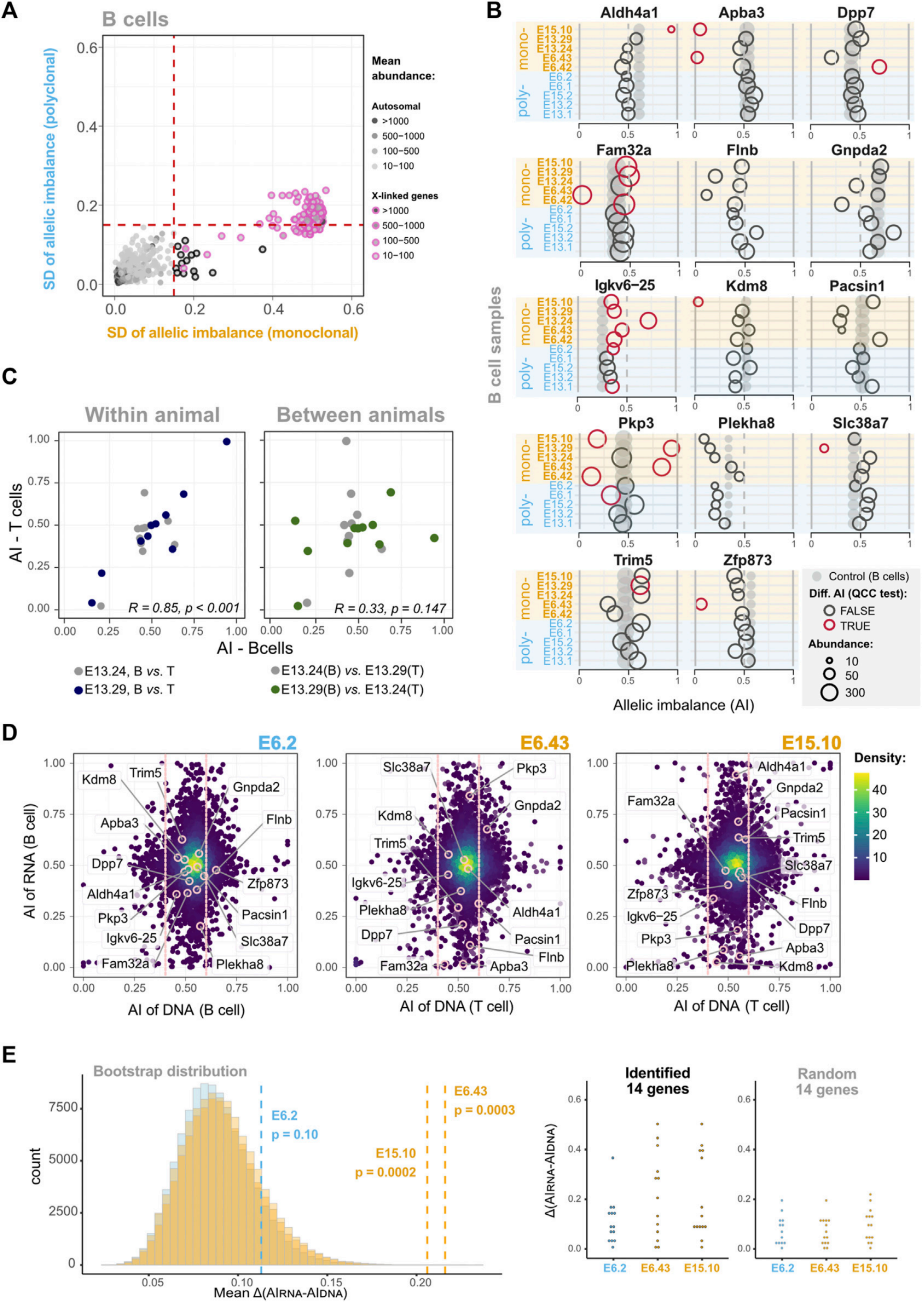
In some *loci*, the memory of allele-specific gene regulatory state persists over many cell divisions throughout extensive differentiation. **(A)** Dot plot showing standard deviations (SD) of AIs for five B cell monoclonal samples (*x-axis*) against the SD of AIs for five polyclonal samples (*y-axis*). Dashed vertical and horizontal lines—arbitrarily set at an AI SD of 0.15—represent the threshold above which genes were considered as potentially intrinsically imbalanced. Dots represent genes, black-circled dots highlight genes with higher AI variance among monoclonal samples in the autosomes, while pink-circled dots denote the X-linked genes (control). The genes included in this analysis have AI differences statistically significant after QCC correction in at least one pairwise comparison (see the matrix of Figure 3B for all pairwise comparisons) and are expressed in all the 10 B cell samples; see Supplementary Figure S9 for the same SD-based analysis without filtering the genes. **(B)** Comparison of putative mitotically stable allelically imbalanced genes between all B cell samples. Grey dots represent the AI values of the unmanipulated animal control sample, and empty circles are the AI values of monoclonal or polyclonal samples. Red circles represent comparisons for which the AI difference between the manipulated animal sample and unmanipulated control remained statistically significant after QCC correction. Not all genes show statistically significant differences with the unmanipulated control, but all represented genes have at least one statistically significant AI difference in monoclonal or polyclonal pairwise comparisons. The diameter of dots/circles is proportional to the reads abundance. **(C)** Dot plots showing the AI of putative transcriptionally stable allelically imbalanced genes in B cells (*x-axis*) against the corresponding ones in T cells (*y-axis*). Pairwise comparisons for two monoclonal animals are shown. In the left plot, the B and T cell data for each of the two animals are paired (within animal comparison), whereas the right plot is an artificial control in which the B and T cell data from different animals are paired (comparison between animals). Each plot shows the Pearson’s coefficient correlation considering the combined animal datasets; for the left graph, Pearson’s coefficient correlations for each animal are *R* = 0.33 (*p* = 0.147) and *R* = 0.85 (*p* < 0.001). **(D)** AI from RNA-seq data plotted against AI from whole exome sequencing (WES) data for the same animals (polyclonal sample E6.2, and monoclonal samples E6.43 and E15.10). Only genes with abundance>10 TMM-normalized counts are represented. For the DNA axis (*x-axis*), all of these genes fall in the vicinity of the dotted vertical lines highlighting the 0.4–0.6 AI “balanced” range. **(E)** Difference between the AIs in DNA data and RNA data (AI_DNA_−AI_RNA_) in two monoclonal samples for the genes highlighted in **(B)**. In the left panel, the histogram represents the distributions of the means of the difference for 13 or 14 randomly sampled genes generated by bootstrapping the transcriptomics data (100,000 replicates per distribution). The dashed lines show the observed AI_DNA_−AI_RNA_ means for the 13 and 14 of the 14 putative mitotically stable allelically imbalanced genes detected in the monoclonal samples E6.43 and E15.10, respectively, which are statistically different from the mean of a random sample considering the respective distributions (*p* = 0.0003 and *p* = 0.0002, respectively), unlike the AI_DNA_−AI_RNA_ mean for the 14 putative mitotically stable allelically imbalanced genes in the E6.2 polyclonal sample (*p* = 0.10). The right panel shows the distribution of the | AI_DNA_−AI_RNA_ | observed for the putative mitotically stable allelically imbalanced genes and a random sample of size 14 in E6.2 and E15.10, and 13 in E6.43. Whenever present, abundance values are TMM-normalized counts.

Since the previous analyses failed to detect a small percentage of genes with stable epigenetic states, we developed an alternative strategy to scrutinize the dataset further. If a gene has clone-specific AI, then the dispersion of the AI values in monoclonal samples should be higher than in the control group. Thus, we plotted the AI standard deviations of B cell monoclonal (*x-axis*) and polyclonal (*y-axis*) samples, for all the genes that had shown a statistically significant differential AI in at least one of the pairwise comparisons, as long as the gene was expressed in all the samples (Supplementary Table S4). The plot highlighted 14 genes with higher standard deviation dispersion values in the monoclonal set than in the polyclonal set (Figure 4A; Supplementary Figure S9). The fact that, above a threshold of standard deviation, no gene is found to have a standard deviation in the polyclonal set higher than in the monoclonal set suggests that the identified genes are not exceptions due to the multiple comparisons that were performed (*p* < 2.7 × 10^−6^, one-sided Wilcoxon test). The representation of these genes’ AI values for each animal confirms the higher standard deviation dispersion in the monoclonal group compared to the polyclonal group (Figure 4B). This shows that as the cells in the monoclonal animals suffered the same differentiation programs when expanding from the HSC to the B cells, each revealed unique allele-specific stable transcriptional states. *Pkp3* is a clear example. We can see that in some monoclonal samples *Pkp3* is transcribed mostly from the maternal allele, whereas in others it is the paternal transcript that dominates and there is also a case of balanced expression from both alleles. To further confirm that this observation is biologically meaningful and not a statistical artifact, we took advantage of the NGS transcriptomic data produced for the T cells from two monoclonal mice (E13.24 and E13.29). B and T cells share the lineage up to the common lymphoid progenitor and then split into independent lineages. If the stable clonal AI biases found in some B lineage genes were already present in the HSC, in T cells it is reasonable to expect that these genes, if expressed, kept the original AI biases. To test this prediction, we plotted the B and T AI pairwise values for each gene from each of the two animals (Figure 4C). The plot clearly shows a strong correlation of the dataset within each of the animals (E13.24 and E13.29). To produce an artificial control set, we then associated, for each gene, the B cell AI value from animal 1 to the T cell value of animal 2 and vice-versa, which eliminated the correlation. Clearly, we have found persistent patterns of allelic expression that are already present in HSC and are then independently preserved by the B and T cells from each animal. However, before concluding that these findings are evidence of stable epigenetic states, the possibility that these few examples result from the loss of heterozygosity (LOH) events should be addressed. In the clonal mice, during the initial stage of reconstitution, when the number of progenitor cells is low, any genetic event in a progenitor cell affecting an allele’s expression could have a sizable impact on the AI levels of the emerging populations. Thus, we performed exome sequencing in a subset of samples to evaluate whether B6 and CAST’s exons are equally represented for these 14 genes (Figure 4D). The data revealed no obvious LOH for any of the genes involved. In addition, these 14 genes have not been associated with LOH or replication fragile sites and lack the molecular features typically associated with these regions, such as high expression levels and a large size (Helmrich et al., 2006; Barlow et al., 2013). Finally, we performed a bootstrap analysis (100,000 replicates per distribution) to evaluate the likelihood of randomly finding a group of genes with the mean difference between the AIs in DNA and RNA data (AI_DNA_—AI_RNA_) as high as the ones we found in the monoclonal animals (Figure 4E). We focused on monoclonal animals for which we generated transcriptomics and exome sequencing data. The bootstrapping revealed that, for E6.43 and E15.10, random sampling is unlikely to produce a group of genes with higher AI_DNA_—AI_RNA_ mean differences than the ones we found (*p* = 0.0003 and *p* = 0.0002, respectively). Taking into account the absence of obvious LOH as measured by the exome sequencing, the *p*-values of the bootstrapping analysis, and the fact that the two monoclonal animals investigated for LOH were from independent experiments, we conclude that the high standard deviation of the AI values for these 14 genes is not a result of LOH and likely reflects stable transcriptional biases originally present in the cloned HSC. Finally, genes with monoallelic expression have often been linked to the chromosomal regions with asynchronous DNA replication timing (AS-RT; Chess et al., 1994; Mostoslavsky et al., 2001; Singh et al., 2003; Ensminger and Chess, 2004). A recent study (Blumenfeld et al., 2021) mapped genome-wide replication asynchrony in B cell clones using the same F1 mouse cross. We assessed the intersection of the 14 RME genes we identified in single HSC-derived populations and the AS-RT regions listed in Blumenfeld et al. (2021). No intersections were detected. However, the numbers of genes and clones are insufficient to reach clear conclusions.

### Abelson Clones Show a Higher Number of Genes With Clonal Specific AI Than Lymphocytes Differentiated From a Single HSC

**FIGURE 5:**
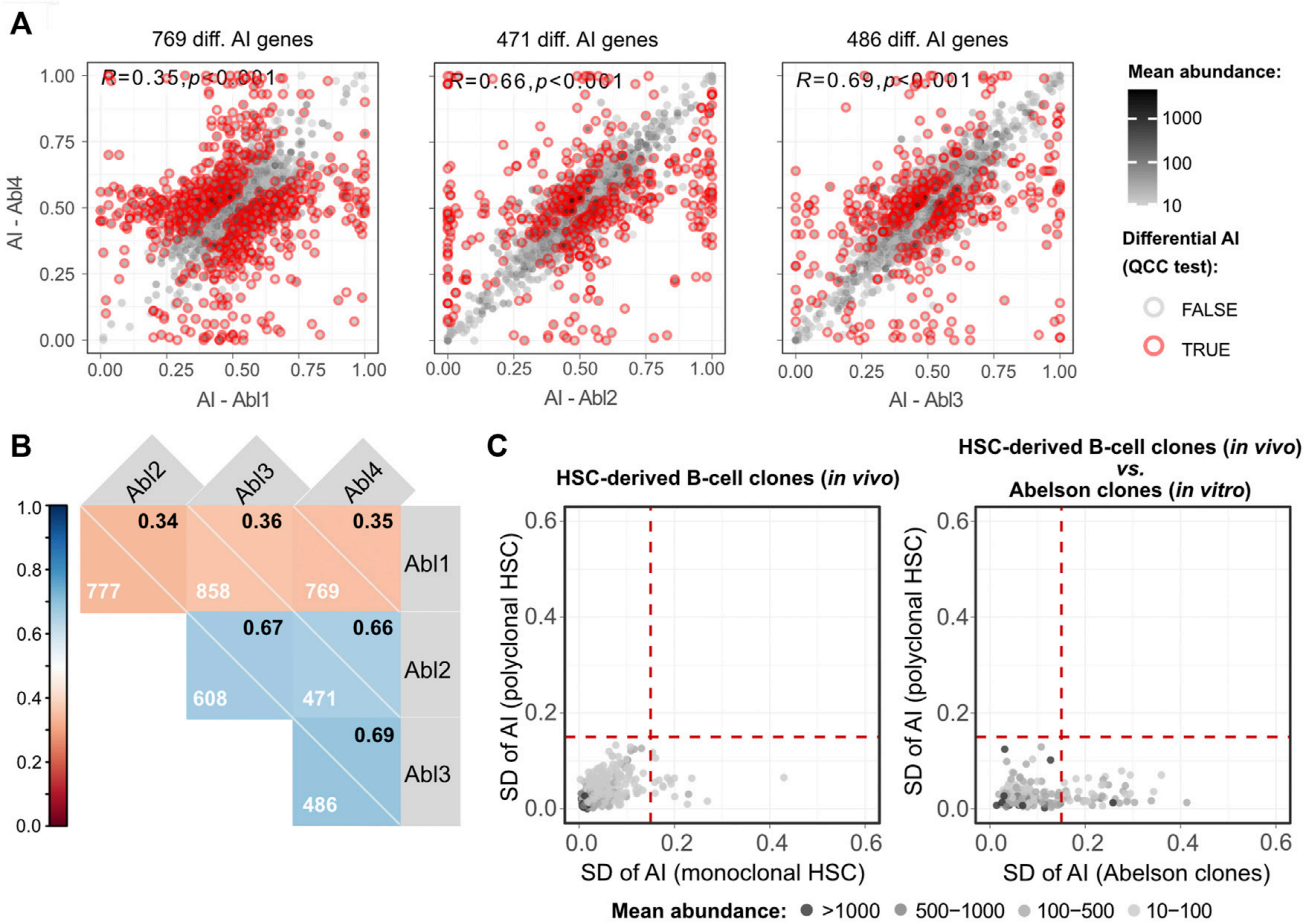
Abelson clones show a higher number of genes with clonal-specific AI than lymphocytes differentiated from a single HSC. **(A)** Representative dot plots of pairwise comparisons of AI between different Abelson-immortalized B cell clones. Pearson’s coefficient correlation of AI and the number of genes with a significant differential AI (after the QCC test) between samples are shown. Mean abundance levels (mean TMM-normalized counts) are represented as continuous grayscale colors. **(B)** Correlogram with pairwise comparisons of Abelson-immortalized B cell clones. Pearson’s coefficient correlation of AI for all pairwise comparisons between samples. Each square shows the Pearson’s coefficient in the upper right corner and the number of genes with a significant differential AI in each pairwise comparison after applying QCC correction on the binomial test. **(C)** Two dot plots showing SDs of AIs for four monoclonal (*x-axis*) against four polyclonal (*y-axis*) HSC-derived B cell samples (left plot), and SD of AI for all four Abelson clones (*x-axis*) against the SD of AI for four polyclonal HSC-derived B cell samples (*y-axis*) (right plot). WES data were used to exclude transcripts with possible loss of heterozygosity. Dashed vertical and horizontal lines represent the threshold above which genes were considered as potentially intrinsically imbalanced and were arbitrarily set at an AI SD of 0.15. Mean abundance levels (mean TMM-normalized counts) are represented as binned grayscale colors.

One lingering question is to what extent allele-specific expression states persist in clonal populations over multiple differentiation steps. Our analysis suggests that the incidence of such stable states is much lower than was previously reported in clonal cells not undergoing differentiation (Eckersley-Maslin et al., 2014; Gendrel et al., 2014; Gimelbrant et al., 2007; Jeffries et al., 2012; Zwemer et al., 2012). However, in this work we used a much more stringent statistical approach to allele-specific analysis, relying on technical replicates for RNA-seq libraries to exclude false positives (Mendelevich et al., 2021). This raises the possibility that the differences could be due, at least in part, to the differences in experimental and statistical procedures compared to previous studies. To exclude this potential source of discrepancy, we applied the same analytical pipeline to RNA-seq data generated from clonal cells that grew without differentiation. We used the v-Abl pro-B clonal cell lines Abl.1, Abl.2, Abl.3, and Abl.4, which were derived previously from 129S1/SvImJ (129S1) x CAST/EiJ (CAST) F1 female mice (Zwemer et al., 2012), with two replicate RNA-seq libraries prepared and sequenced per sample (Gupta et al., 2021). To control for the possible LOH, exome sequencing data for the Abl.1-4 clones (Gupta et al., 2021) was considered. Genes whose genomic DNA showed total allelic counts of <10 or 0.3<AI>0.7 were excluded from RNA-seq analysis. We found that all pairwise comparisons have at least fourfold more genes with significant differences (Figures 5A,B; Supplementary Figure S10) than the pairwise comparison of CAST x B6 HSC-derived clones with the highest number of genes with significant differences (Figure 3B). Furthermore, the AI values in the collection of Abelson clones also have a higher dispersion than the collection of the HSC-derived clones (Figure 5C). It is unlikely that these massive differences result from genetic differences between 129S1 and B6 because the two strains share an ancestor after the split from CAST (Witmer et al., 2003). The data suggest that in clones undergoing differentiation there is erasure and intraclonal reestablishment of AI.

## Discussion

There is an ongoing debate on whether phenotypic diversity due to epigenetics or somatic DNA recombination is a general phenomenon that improves the function of defined cellular populations. There is also an open discussion on the quantification of clonal RME in autosomal genes and whether this is a widespread phenomenon *in vivo* or a characteristic of clones grown *in vitro* (Reinius and Sandberg, 2018; Vigneau et al., 2018; Rv et al., 2021). To address the latter question, we have performed a thorough analysis of random allelic expression biases in clonal B and T cell populations emerging *in vivo* after prolonged and extensive lineage differentiation in mice injected with single murine HSCs. The accepted model of establishment and maintenance of RME asserts that the allelic biases are established during differentiation stages and are stably propagated across subsequent differentiation steps (Gendrel et al., 2014; Marion-Poll et al., 2021). We report two surprising findings that lead us to update this model. First, the analysis of these monoclonal and genetically unmanipulated hematopoietic systems allowed us to conclude that after prolonged (more than 4 months between HSC transfer and collection) and extensive cell division and lineage differentiation, the percentage of autosomal genes displaying RME is much lower than the estimates from collections of clones grown *in vitro* [<0.2% vs*.* ∼2%–15% (Gimelbrant et al., 2007; Jeffries et al., 2012; Zwemer et al., 2012; Eckersley-Maslin et al., 2014; Gendrel et al., 2014)], suggesting that, if present *in vivo*, the presumed stable allelic transcriptional states that are established at each differentiation step are meta-stable across differentiation stages, i.e., they are (progressively) erased and reestablished along the differentiation steps. This inference is, to our understanding, the simplest explanation for the almost complete lack of biases we report in B and T cells derived from a single HSC. It is improbable that an HSC, which has already gone through a differentiation process, would have fewer stable allelic biases than an embryonic stem cell, which has been shown to carry hundreds of allelic biases (Eckersley-Maslin et al., 2014). However, we cannot exclude entirely the possibility that HSCs are remarkable for carrying almost no random allelic biases, as we observed in the derived B and T cells. Second, to our knowledge, we have identified for the first time rare regions in the autosomal chromosomes that keep stable allelic transcriptional states along lineage differentiation in the hematopoietic lineage. Below we discuss the implications of the technique we used and the findings for XCI, hematology, RME, and phenotypic diversity.

### XCI in a Monoclonal Hematopoietic System

XCI has relied on the analysis of rodent/human somatic cell hybrids (Brown et al., 1997), primary human cell lines (Carrel and Willard, 1999), murine or human embryonic stem cells (Pintacuda and Cerase, 2015; Patel et al., 2017), murine and human-induced pluripotent stem cells (Fan and Tran, 2011), and transgenic mice with a genetically engineered *Xist locus* (Berletch et al., 2015). The former are *in vitro* systems, and the latter is an animal model in which the activation of one of the X chromosomes is imposed due to the deletion of *Xist*. Here we show that it is possible to study lineage-specific chromosome inactivation *in vivo* using genetically unmanipulated cells. Single-HSC reconstitution of mice identified genes escaping from XCI in B and T cells that had been previously identified in different tissues (Yang et al., 2010; Li et al., 2012; Wu et al., 2014; Berletch et al., 2015). Given the extraordinary differentiation of the hematopoietic cells from the HSCs, the interest in tissue-specific epigenetics (Sierra and Anguera, 2019), and the possibility of reactivation of X chromosome in lymphocytes (Wang et al., 2016; Syrett et al., 2019), this system can be used to produce an atlas of lineage-specific XCI in the blood cells in mice and potentially also in human cells, if single human HSCs are shown to produce monoclonal human hematopoietic systems in reconstituted mice (Beyer and Muench, 2017).

### Autosomes Versus X Chromosome Parallels

RME in autosomal regions and XCI have in common the stochastic component leading to expression vs*.* silencing. Since the 1990s, this common feature has recurrently tempted many to draw parallels between XCI and RME in reviews or opinion pieces [e.g., (Efstratiadis, 1995; Goldmit and Bergman, 2004; Chess, 2016; Gendrel et al., 2016)] and also original articles [e.g., (Mostoslavsky et al., 2001; Pereira et al., 2003)]. At the molecular level, three observations stand out. First, at least one gene has been found to play a role in XCI and RME (Mould et al., 2013). Second, high concentrations of long interspersed nuclear element sequences, which were implicated in XCI (Chow et al., 2010), have been proposed to characterize *loci* involved in RME (Allen et al., 2003). Finally, three non-coding RNA autosomal genes, *ASAR6, ASAR6-141,* and *ASAR15*, display *XIST*-like features because they are monoallelically expressed, remain associated with the chromosome from which they are expressed, were shown to silence the nearby alleles, and their disruption resulted in delayed replication timing and the reactivation of previously silent alleles of nearby genes (Stoffregen et al., 2011; Donley et al., 2013; Donley et al., 2015; Heskett et al., 2020). Despite these possible common mechanistic features, our study established a fundamental difference: during lineage differentiation, RME lacks the stability of XCI. This stability is due to a multilayered process of gene and chromosome silencing (Dossin and Heard, 2021) that, if present in regions of the autosomes, would probably compromise the dynamics needed for the onset of the different programs of lineage commitment during hematopoiesis.

### Applications of Stable Imprints in the Autosomal Regions

The identification of a few regions in the autosomal chromosomes with stable epigenetic states in the hematopoietic lineage could be explored in the future to develop clonality assays for the hematopoietic system. These assays have typically relied on finding significant skewing of the XCI ratio from the 1:1 ratio, which is limited to females and has a low resolution (Ayachi et al., 2020). By focusing on polymorphisms in the autosomal regions with stable epigenetic states, it should be possible to design clonality assays for both sexes that are more sensitive to decreases in HSC output than the assays based on XCI.

### Models of RME

**FIGURE 6:**
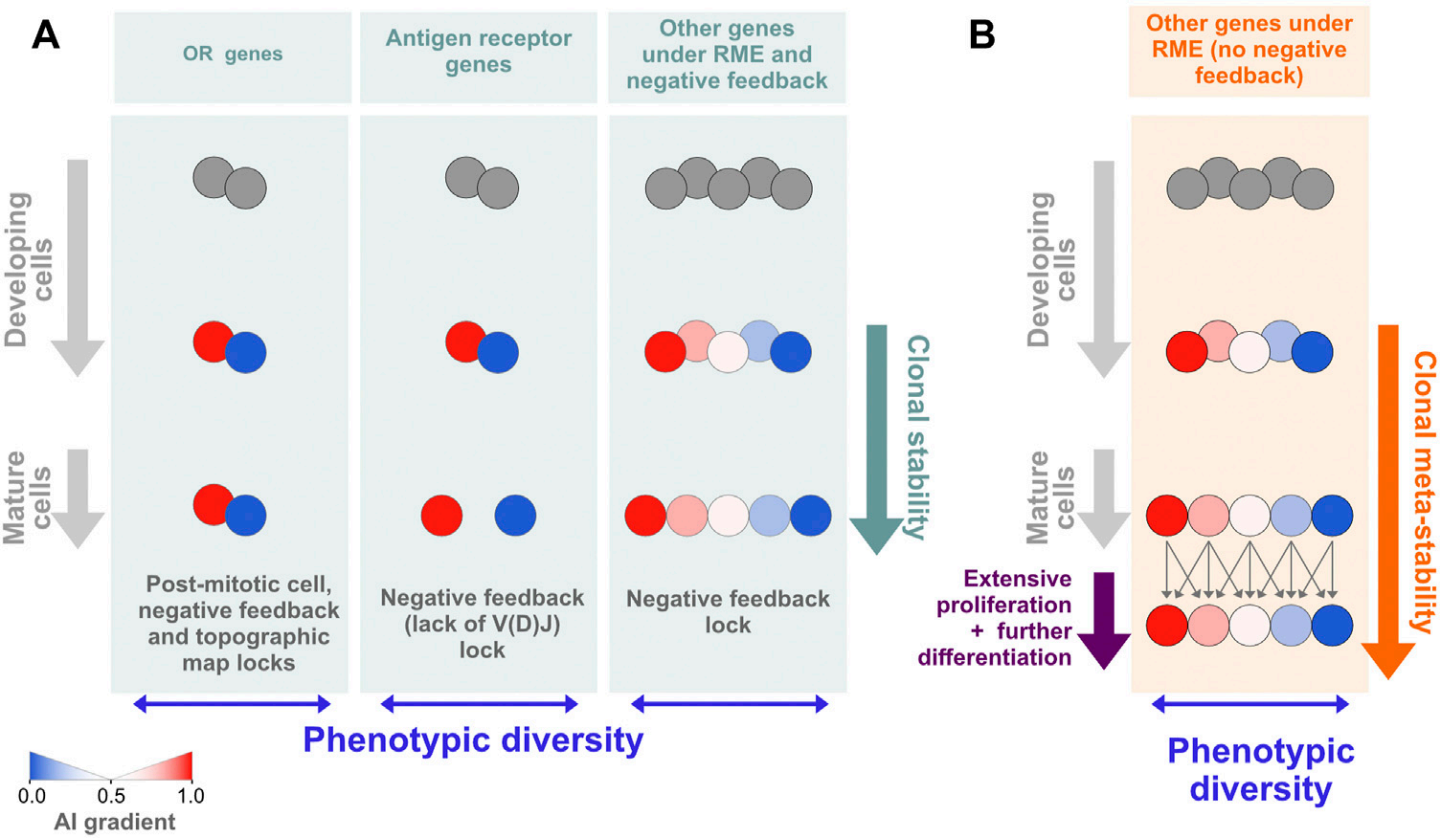
Models of RME. **(A)** For most autosomal genes under RME, the epigenetic states leading to allelic biases are established *de novo* during differentiation and shortly before the genes are expressed. This model of RME is characterized by documented (e.g., olfactory receptor and antigen receptor genes) or probable clonal stability due to the existence of locks that stabilize the AI [reviewed in (Barreto et al., 2021)]. One notable lock is the negative feedback triggered by the protein expression of one allelic form that prevents further gene or allelic activation (or recombination, in the case of the antigen receptors). **(B)** A model of RME in which the AI for each clone is meta-stable, i.e., it can change within a certain range during extensive periods of proliferation and differentiation. Assuming that HSCs have an initial percentage of genes under RME close to that estimated for cells from collections of developmentally frozen clones grown *in vitro*, our data are compatible with a meta-stable model of RME.

As a way to reconcile the lack of allelic expression imbalances in extensively differentiated *in vivo* grown clones with the data on *in vitro* grown clones that do not undergo differentiation in culture and show higher levels of AI, we propose that the evolutionary selection pressure shaping RME is at the level of the phenotypic diversity displayed by a population of cells, which does not absolutely require the perpetuation of the allelic biases at the clonal level. What should be crucial is that, within a given developmental stage, the cells keep distinct allelic biases, but these may be established only shortly before the cell population becomes phenotypically defined. This solution is simpler and more economical than the one based on marks that are established early and then propagated for many divisions before the genes in those regions are expressed. In an extreme view of this model, the cells may change the AI stochastically from one stage of differentiation to the next (Figure 6B). The key idea of the model is the uncoupling of population phenotypic diversity from clonal stability. These two concepts are typically seen as intertwined. For decades, the classic examples of autosomal RME and the generation of phenotypic diversity within initially isogenic cell populations have been the antigen and odorant receptors, for which the univocal association between the phenotype and the clone or long-living cell is essential. In the case of the antigen receptors, the stability of the phenotype is required because the process of V(D)J recombination that builds a functional antigen receptor gene is coupled to stringent negative and positive cellular selection steps in the bone marrow or the thymus, and the emerging clone is not allowed to completely reinvent its antigen receptor after exiting the primary lymphoid organs. Although for a different reason, which is the preservation of the topographic map of the olfactory experience throughout life, each olfactory sensory neuron is also committed to the expression of a single odorant receptor gene (and allele). These examples of phenotypic diversity are spectacular but also exceptional in the sense that an antigen receptor gene depends on a unique process of somatic DNA recombination, and the odorant receptor genes make up the largest gene family in the mammalian genome. The RME of less unique genes, particularly in the blood cells, which circulate permanently, may be better described using the cell population dispersion of AI values as a proxy for phenotypic diversity at a given moment (Figure 6B) rather than as a collection of clones with immutable AI values throughout lineage differentiation (Figure 6A). This working hypothesis turns RME into a more dynamic process than what is normally assumed when considering mitotically inherited RME, but it should not be confused with the stochastic or dynamic RME detected by single-cell RNA-seq (Reinius et al., 2016) because it has a longer time-scale and thus it is not explained by transcriptional bursts.

### Limitations and Future Prospects

This study was designed to provide the first direct *in vivo* comparison between the stability of XCI and RME of autosomal genes. The data are conclusive in showing that XCI and RME are different phenomena and in the description of stable RME in the autosomes from an HSC to differentiated cells as a rare phenomenon. One limitation of the work is that the real number of genes showing this type of RME could be different from our estimate. It could be lower than 14 *loci* if, for instance, our exome sequencing strategy fails to identify all cases of LOH or other genetic alterations. It could also be higher, if the number of clones we studied is limiting, the specific genetic background used does not distinguish the allelic transcripts from additional autosomal genes under RME or the B cell lineage is not representative of the other hematopoietic lineages with respect to stable RME. Nevertheless, given the evidence we gathered, it is unlikely that no autosomal gene has this stable RME and it is also unlikely that more than 0.5% of the autosomal genes show this pattern. A second limitation is the uncertainty regarding the nature of the stable marks we identified. The study was designed to distinguish between many mitotically stable marks and very few marks or none. We have clearly shown that stable marks already present in the HSCs are extremely rare in the differentiated B cells. Whether all these rare marks are epigenetic or due to somatic genetic variations in the HSCs or early differentiating cells is an open question. Most of the *loci* with stable AI that we identified do not have remarkable features in terms of RNA expression levels or open reading frame size that could make them more prone to accumulate mutations (Supplementary Figure S11). In the most thorough published study, whole-genome sequencing of *in vitro* HSC-derived small clones (about 500 cells) cultured for up to 14 days revealed that each HSC from an 8-month old B6 mouse has about 110 single nucleotide variants (SNVs) and 26 insertions or deletions (indels; Druce, 2021). The majority of these mutations are intergenic, the vast majority of mutations in genes (>98.5%) are not in exons, and most are not expected to lead to changes in transcription. Thus, considering the random distribution of mutations in the genome of HSCs (Druce, 2021), even taking into account a network of gene interactions in which mutations in *trans* could impact the AI value of a given gene (e.g., mutations in transcription factor, RNA interacting proteins or regulatory RNA genes) SNVs and indels are unlikely to produce the highly diverse AI pattern seen for *Pkp3*, with biases present in at least four out of five monoclonal animals (Supplementary Table S5); based on these calculations, a reconstitution advantage of HSC with mutations in *Pkp3* also fails to explain the data. *Pkp3* is thus the most solid example to date of an autosomal gene with stable marks already present in the HSCs that are likely to be epigenetic (Supplementary Table S6). However, genetic variation is a working hypothesis for the several other cases in which the AI standard deviation is mainly affected by a single outlier. Whole-genome sequencing will never be fully conclusive given the complex network of gene interactions. Thus, only future experiments with drugs that interfere with epigenetic marks but not with genetic mutations will clarify this issue. A third limitation is the comparison of our data with the data from Abelson clones. This comparison of allele-specific expression data across distinct experiments (e.g., Abelson clones and reconstituted cell populations) is controlled by the RNA-seq replicates and overdispersion analysis, which remove nearly all between-experiment technical variation (Mendelevich et al., 2021). Thus, our estimate of RME as being much lower than the estimates from *in vitro* clones not undergoing extensive differentiation should hold. However, it would be more informative to compare our findings on clones extensively differentiated *in vivo* from HSCs with *ex vivo* sorted cells from the same animals that are shortly expanded in the absence of major differentiation before the RNA is collected. This is one of the forthcoming experiments, which also include an investigation of RME frequency as a function of the degree of differentiation from the progenitor to the tested population, the characterization of the *cis*-regulatory features of genes showing stable RME from the HSC to differentiated cells, and the reappreciation of the apparent interconnection between clonal stability and phenotypic diversity.

## Materials and Methods

### Animal Breeding

All mice were bred and maintained at the specific pathogen-free animal facilities of the Instituto Gulbenkian de Ciência (IGC, Oeiras, Portugal). C57BL/6J-Ly5.1 (C57BL/6J strain carrying the pan-leukocyte marker Ly5.1), C57BL/6J-Ly5.2 (C57BL/6J strain carrying the pan-leukocyte marker Ly5.2), and CAST/EiJ were originally received from The Jackson Laboratory (Bar Harbor, ME, United States). Animals used in reconstitution experiments were bred at our animal facility to generate female heterozygous F1 donor (CAST/EiJ × C57BL/6J-Ly5.2) and recipient (CAST/EiJ × C57BL/6J-Ly5.1) animals. Donor animals used in cell transfer experiments were <5 week-old and recipient animals were 5–16 week-old. This research project was reviewed and approved by the Ethics Committee of the IGC and by the Portuguese National Entity that regulates the use of laboratory animals.

### HSCs Isolation

The bone marrow was flushed out and single-cell-suspended with FACS buffer (1 × PBS, 2% FBS) from the tibia and femur using a syringe. The erythrocytes were lysed with red blood cell lysis buffer (RBC lysis buffer; 155 mM NH_4_Cl, 10 mM NaHCO_3_, 0.1 mM EDTA, pH 7.3) for 5 min and immediately rinsed and washed with FACS buffer. The cells were blocked with FcBlock (anti-CD16/32) for 15 min at 4°C and washed. Enrichment for lineage-negative cells was performed by incubating the cell suspension with a cocktail of biotin-conjugated antibodies for surface markers of lineage-committed cells (anti-CD45R/B220, anti-CD19, anti-CD11b/Mac1, anti-Ly-76/Ter119, anti-Ly6G/Gr1, and anti-CD3) and, subsequently, lineage-marked cells were depleted using MACS Streptavidin MicroBeads (Miltenyi Biotec) for negative selection of lineage-positive cells by immunomagnetic separation using a MACS column (Miltenyi Biotec). Cells were further stained with PI and fluorophore-conjugated antibodies: APC-conjugated anti-c-Kit, PE-Cy7-conjugated anti-Sca-1, BV421-conjugated anti-CD48, PE-conjugated anti-CD150, and Streptavidin-APC-Cy7, to isolate LH-HSCs (Kiel et al., 2005). Single LT-HSCs were sorted on a FACSAriaIII using the single-cell deposition unit into the individual wells of Terasaki plates (no. 452256, MicroWell 60-well MiniTray, Nunc Brand, Thermo Fisher Scientific Inc.) preloaded with 15 μl of FACS buffer. Each well was examined in a 4°C room using an inverted microscope and only the wells with a unique cell were used in the reconstitutions.

### Animal Reconstitutions

Recipient females (5–16 week-old) received a sublethal 600 cGy dose of whole-body gamma irradiation (Gammacell 2000 Mølsgaard Medical), 2–6 h before an intravenous retro-orbital injection with a single HSC or 50–200 HSCs. Recipient animals were analyzed routinely 4 weeks after injection and every 2 weeks for up to 12 weeks for the presence of chimerism in the peripheral blood. Blood samples were collected from the submandibular vein in 0.5 M EDTA, erythrocytes were lysed using RBC lysis buffer, and the cells were then stained with PE-conjugated anti-Ly5.1 and FITC-conjugated anti-Ly5.2 antibodies, and analyzed on a FACSCanto or FACScan.

### Processing of Animal Samples

Animals showing chimerism 12 weeks post-reconstitution were sacrificed and processed by removing thymi, spleens, and bone marrow. Single-cell suspensions from bone marrow were obtained as described above using a syringe, and a 70-μM nylon mesh for the spleen and thymus. Erythrocytes were lysed with RBC lysis buffer for 5 min and immediately rinsed and washed with FACS buffer. Around 30% of the cell suspension from bone marrow was saved for reconstitution of sublethally irradiated secondary recipient female mice, which were injected by intravenous retro-orbital administration, and analyzed for chimerism 4 weeks post-injection as described above. Different stainings with labeled antibodies were used to analyze and sort lymphoid populations in the spleen and thymus and the myeloid population in bone marrow or spleen with FACSAriaIII, after cell blocking with FcBlock (anti-CD16/32). In experiment 6, a combination of PI, APC-Cy7-conjugated anti-Ly5.1, and PE-conjugated anti-Ly5.2 was used with markers PE-Cy7-conjugated anti-CD19, APC-conjugated anti-IgM, and BV786-conjugated anti-Mac1 for spleen; PE-Cy5-conjugated anti-CD4 and BV605-conjugated anti-CD8 for thymus; and PE-Cy5-conjugated anti-B220 and APC-conjugated anti-IgM for bone marrow. In experiments 13 and 15, a combination of PI, FITC-conjugated anti-Ly5.1, and PE-conjugated anti-Ly5.2 was used with markers PE-Cy7-conjugated anti-CD19 and APC-conjugated anti-IgM for spleen; PE-Cy7-conjugated anti-CD4 and BV605-conjugated anti-CD8 for thymus; and BV786-conjugated anti-Mac1 for bone marrow.

### RNA Extraction

After cell sorting, pellets were harvested by centrifugation and resuspended in 0.25 ml of TRIzol Reagent or 0.1 ml of Absolutely RNA Nanoprep Kit (Agilent #400753) lysis buffer. Homogenized samples were stored at −80°C until RNA isolation, which was performed according to the manufacturer’s protocols.

### Monoclonality Screening

To test for monoclonality before sequencing, RNA was isolated from the same repopulated animals using sorted cell populations other than the sequenced ones. cDNA was prepared using SuperScript IV (ThermoFisher #18090050) following the manufacturer’s recommendations. Two sets of primers, each flanking a different SNP of *Xist*, were used, namely: Fw1 5′aga​cgc​ttt​cct​gaa​ccc​ag with R1 5′aag​atg​ctg​cag​tca​ggc; and Fw2 5′gga​gtg​aag​agt​gct​gga​gag with R2 5′gtc​agt​gcc​act​att​gca​gc. PCR was performed with GoTaq DNA polymerase (Promega #M3005) using the following program: 5 min at 95°C, 45 cycles of 30 s at 95°C, 30 s at 60°C, and 25 s at 72°C, and a final elongation of 7 min at 72°C. The amplicons were separated in agarose gel, purified, and sequenced by Sanger sequencing with Fw1 or R2 primers.

### cDNA Library Preparation and Whole-Transcriptome Sequencing

Omega Bioservices, USA, performed cDNA library preparation and whole-transcriptome sequencing. According to the manufacturer’s protocol, RNA-seq libraries were prepared using SMART-Seq v4 Ultra Low Input RNA Kit (Clontech). Technical replicates of 10 ng of RNA were used as input. The RNA was primed by an oligo(dT) primer (3′ SMART-Seq CDS Primer II A), and first-strand cDNA synthesis was performed at 42°C for 90 min and 70°C for 10 min. The resulting cDNA was then amplified *via* PCR using the following program: 1 min at 95°C, eight cycles of 10 s at 98°C, 30 s at 65°C, and 3 min at 68°C, and a final elongation of 10 min at 72°C. 15–200 pg full-length cDNA was tagged and fragmented by the Nextera XT transposome (Illumina) and amplified by PCR: 30 s at 95°C, 12 cycles of 10 s at 95°C, 30 s at 55°C, and 30 s at 72°C, then 5 min at 72°C. Mag-Bind RxnPure Plus magnetic beads (Omega Bio-tek) were used to purify the library and provide a size-selection step. The libraries were then pooled in equimolar concentrations and sequenced on Illumina HiSeq 2500 machine (150 bp, paired-end).

### Allele-Specific Gene Expression Analysis From RNA-Seq

RNA-seq data analysis for AI estimation followed the ASEReadCounter* tool adapted from the GATK pipeline (Castel et al., 2015) for the pre-processing read alignment steps up to allele counts, and the statistical R package Qllelic.v0.3.2 for calculation of the QCC and estimation of confidence intervals for differential AI analysis (Mendelevich et al., 2021). RNA-seq reads were trimmed with cutadapt.v.1.14 using the wrapper trim_galore to remove nextera adapters. Sequencing reads were aligned with the reference genome (maternal) and imputed (paternal) with the STAR aligner v.2.5.4a, with default filtering parameters and accepting only uniquely aligned reads. Samtools mpileup (v.1.3.1) was used to estimate allele-specific coverage over SNPs. Gene models were generated by collapsing all exons belonging to the same gene, based on the GRCm38.68 RefSeq GTF file downloaded from ftp://ftp.ensembl.org/pub/release-68/gtf/, where overlapping regions belonging to multiple genes were excluded. Point estimates of AI for a gene were obtained as the ratio of maternal allele counts over total allelic gene counts. Gene abundance counts were obtained with featureCounts from the same bam files generated with the ASEReadCounter* alignment pipeline, and abundance was estimated as TMM-normalized counts with edgeR (Robinson and Oshlack, 2010). Genes with substantially low expression (<10 TMM-normalized counts) and nonrandom allelic biases (i.e., genes with AI lower than 0.1 or higher than 0.9 in all the 11 B cell samples or all the five T cell samples), which can be due to differences or parental imprinting, were removed from the analysis. Genes showing evidence of LOH in at least one sample, obtained from whole-exome sequencing (WES) data, were removed entirely from the analysis (in all samples). In all samples (polyclonal and monoclonal) of E6 the same large deletion was found. To mitigate the impact of *trans* effects from this deletion on the AI results at the genome-wide level, we used more stringent exclusion criteria (i.e., genes with AI lower than 0.25 or higher than 0.75 in all the four samples were removed from the analysis for B cells).

### XCI Escapees

An X-linked gene was considered an XCI escapee if substantial expression from the inactive X chromosome was identified in the single-HSC derived sample. The comparisons were performed by applying the binomial test with QCC correction for technical replicates (Mendelevich et al., 2021). To consider a gene as an escapee, we defined three criteria: 1) in at least two samples from the same tissue (B or T cells) or at least one B cell sample and one T cell sample, the AI value must be above (or below) the median AI value of all genes plus (or minus) 0.1 when the CAST (or B6) X chromosome is expressed; 2) the expression must be higher than 10 TMM-normalized counts (Robinson and Oshlack, 2010); 3) and the median of AI in the control samples (polyclonal and non-clonal samples) must be balanced (0.5 ± 0.2) (Supplementary Figure S7).

### VDJ Clonotypes

Immunoglobulin rearrangements were detected with MiXCR-3.0.12 (Bolotin et al., 2015; Bolotin et al., 2017), by alignment of RNA-seq raw reads with reference germline V, D, J, and C gene sequences and assembly into clonotypes using the same analysis tool.

### DNA Library Preparation and Whole-Exome Sequencing

DNA was recovered from samples (E6.2-B220^+^IgM^+^ from bone marrow, E6.43-CD4^+^CD8^−^ from thymus, E15.10-CD4^+^CD8^−^ from thymus) stored in TRIzol Reagent according to the instructions of the manufacturers, resuspended in DNase-free water, and stored at −20°C. Novogene, UK, performed DNA library preparation and WES using Agilent SureSelect Mouse All ExonV6 kit (Agilent Technologies) following the manufacturer’s recommendations, and x index codes were added to attribute sequences to each sample. The genomic DNA samples were randomly fragmented by sonication (Covaris) to the size of 180–280 bp fragments. The remaining overhangs were converted into blunt ends via exonuclease/polymerase activities. After adenylation of 3′ ends of DNA fragments, adapter oligonucleotides were ligated. DNA fragments with ligated adapter molecules on both ends were selectively enriched in a PCR reaction. The libraries were hybridized with biotin-labeled probes, and magnetic beads with streptomycin were used to capture the exons. After washing beads and digesting the probes, the captured libraries were enriched in a PCR reaction to add index tags. The products were purified with the AMPure XP system (Beckman Coulter). DNA libraries were sequenced on an Illumina platform (150 bp, paired-end). Read alignment and allele counts were based on the ASEReadCounter* pipeline; genes with total allelic counts of <10 and genes with nominal AI >0.75 or <0.25 were excluded.

### Abelson Clones

The v-Abl pro-B clonal cell lines Abl.1, Abl.2, Abl.3, and Abl.4 were derived previously from 129S1/SvImJ × CAST/EiJ F1 female mice by expansion of FACS-sorted single cells after immortalization (Zwemer et al., 2012). Immortalized B cell clonal lines were cultured in Roswell Park Memorial Institute (RPMI) medium (Gibco), containing 15% FBS (Sigma), 1 × L-Glutamine (Gibco), 1 × Penicillin/Streptomycin (Gibco), 0.1% β-mercaptoethanol (Sigma). The culture medium also contained 1% DMSO. On day 2 of the culture, 5 × 10^6^ live cells were collected after sucrose gradient centrifugation (Histopaque-1077, Sigma, Cat 10771), and RNA was extracted from 2 × 10^5^ cells using Sera-Mag SpeedBeadsTM (GE Healthcare), a magnetic beads-based protocol. Two libraries were prepared per clone using the SMARTseqv4 kit (Clontech), starting with 10 ng input RNA for each library according to manufacturer’s instructions. Abl.1 clone was sequenced on the Illumina NextSeq 500 machine (75 bp, single-end); clones Abl.2, Abl.3, and Abl.4 were sequenced on Illumina HiSeq 4000 machine (150 bp, paired-end). RNA-seq data analysis followed the same pipeline as for HSC-derived clones *in vivo*, with exception of the maternal reference genome, which was 129S1/SvImJ. These data were originally generated for the work described by Gupta et al. (2021).

The remaining cells after sucrose gradient collection were washed with 1 × PBS and frozen on dry ice for genomic DNA extraction by GenElute Kit (Sigma, #G1N10-1KT). LC Sciences (TX, United States) performed library preparation, QC, and WES (50x). SureSelect (Agilent Technologies) was used for exome capture following recommendations of the manufacturer. Reads were generated using a Hiseq X Ten sequencing instrument (Illumina; 150 bp, paired-end). Read alignment and allele counts were based on the pipeline used for the RNA-seq of Abelson clone genes with total allelic counts of <10 and those with nominal AI >0.7 or <0.3 were excluded (Gupta et al., 2021).

### Statistical Analysis

The difference between the AI point estimates of two clones, or the difference of point estimate and a threshold (e.g., X-chr escapees), was accepted as significant after accounting for experiment-specific overdispersion of two technical replicates using the R package Qllelic.v0.3.2 (Mendelevich et al., 2021).

## Data Availability

The datasets presented in this study can be found in the NCBI GEO repository with the GSE174040 and GSE144007 accession numbers.
