# Supplementary material for: In Vivo Clonal Analysis Reveals Random Monoallelic Expression in Lymphocytes That Traces Back to Hematopoietic Stem Cells
Source: Front Cell Dev Biol. 2022 Aug 8;10:827774. doi: 10.3389/fcell.2022.827774 (PMC9393635; doi:10.3389/fcell.2022.827774)
Supplement: Supplementary file 1 [file DataSheet1.pdf]

Supplementary Data

Supplementary Tables

Supplementary Table 1. Percentages of reconstituted animals for 16 experiments at 12 weeks post injection.

Experiment#	1 HSC			50 or 200 HSCs		
	**#injected animals**	**#reconstituted animals**	**% of reconstituted animals**	**#injected animals**	**#reconstituted animals**	**% of reconstituted animals**
**1**	29	2	7	4	4	100
**2**	28	1	4	2	0	0
**3**	26	4	15	2	2	100
**4**	21	2	10	2	2	100
**5**	54	3	6	3	2	66
**6**	58	11	19	4	4	100
**7**	21	1	5	4	4	100
**8**	18	1	6	2	1	50
**9**	26	1	4	2	1	50
**10**	15	0	0	2	2	100
**11**	15	0	0	2	0	0
**12**	16	0	0	2	0	0
**13**	34	2	6	2	2	100
**14**	36	0	0	2	2	100
**15**	26	4	15	2	2	100
**16**	30	3	10	2	2	100
mean			*7.7*			*76.9*

Supplementary Table 2. List of genes with nonrandom allelic imbalance (AI) in the B and T cell samples of this study (TMM-normalized counts > 10) or reported in the literature to display parental imprinting.

**B cells**																		
								**AI**									**Abundance**	
**Gene**	**Chr**	**Biased?**	**Imprinted**	**Control**	**E13.1**	**E13.2**	**E15.2**	**E13.24**	**E13.29**	**E15.10**	**E6.1**	**E6.2**	**E6.42**	**E6.43**	**Mean(AI)**	**Std(AI)**	**Mean**	**Std**
Dyrk3	chr1	yes	-	0.10	0.12	0.11	0.11	0.07	0.08	0.08	0.10	0.13	0.12	0.08	0.10	0.02	29.45	6.98
Cyp27a1	chr1	yes	-	1.00	0.99	1.00	1.00	1.00	0.99	0.99	0.99	0.99	-	0.99	0.99	0.00	26.62	8.49
Kcnk2	chr1	yes	-	0.00	0.00	0.00	0.00	0.01	0.01	0.00	0.01	0.01	0.00	0.02	0.01	0.01	20.76	5.10
Gm10075	chr1	yes	-	-	0.90	0.93	0.91	0.90	0.93	0.90	0.91	0.86	0.90	0.88	0.90	0.02	14.89	4.55
A530040E14Rik	chr1	yes	-	0.06	0.09	0.08	0.03	0.12	0.02	0.01	0.03	0.03	0.03	0.07	0.05	0.04	34.66	9.75
Nek6	chr2	yes	-	0.05	0.11	0.07	0.07	0.08	0.08	0.14	0.10	0.09	0.11	0.11	0.09	0.02	26.97	6.01
Fmn1	chr2	yes	-	0.01	0.05	0.03	0.01	0.07	0.05	0.02	0.05	0.07	0.07	0.10	0.05	0.03	28.41	7.02
Bmyc	chr2	yes	-	0.98	0.99	1.00	0.97	1.00	1.00	1.00	0.99	1.00	1.00	-	0.99	0.01	18.70	4.62
Gm13699	chr2	yes	-	1.00	1.00	0.99	1.00	0.99	0.99	1.00	0.99	0.97	1.00	0.98	0.99	0.01	52.72	22.39
Gm13654	chr2	yes	-	0.95	0.97	0.96	0.99	0.99	1.00	0.98	1.00	0.99	0.98	0.96	0.98	0.02	30.81	8.13
Gm13456	chr2	yes	-	0.00	0.10	0.07	0.00	-	0.15	0.12	-	-	-	-	0.07	0.06	25.22	6.60
Mir5115	chr2	yes	-	0.00	0.00	0.00	0.00	0.00	0.00	0.00	0.00	0.00	0.00	0.00	0.00	0.00	202.32	118.67
Adamtsl4	chr3	yes	-	0.03	0.04	0.08	0.02	0.08	0.02	0.03	0.01	0.03	0.01	0.03	0.03	0.02	24.18	13.28
Gnb4	chr3	yes	-	0.02	0.04	0.03	0.02	0.12	0.05	0.03	0.01	0.04	0.08	0.08	0.05	0.03	69.14	11.29
Alpk1	chr3	yes	-	0.00	0.02	0.01	-	0.01	0.02	0.01	0.02	0.01	0.03	0.01	0.01	0.01	19.44	6.26
Sec24d	chr3	yes	-	0.11	0.08	0.13	0.07	0.09	0.13	0.12	0.04	0.09	0.06	0.05	0.09	0.03	33.45	12.01
Rpsa-ps10	chr3	yes	-	0.04	0.05	0.07	0.04	0.02	-	0.05	-	-	-	-	0.05	0.02	11.94	0.98
Synpo2	chr3	yes	-	0.00	0.01	0.01	-	-	-	-	0.02	0.08	0.02	-	0.03	0.03	15.36	3.89
Cgn	chr3	yes	-	0.01	0.01	0.02	0.00	0.04	0.05	0.00	0.01	0.04	0.07	0.11	0.03	0.04	14.70	3.38
Coro2a	chr4	yes	-	0.96	0.98	0.95	0.98	0.96	0.94	0.92	0.98	0.95	0.98	0.93	0.96	0.02	55.64	8.31

Alpl	chr4	yes	-	0.04	0.04	0.04	0.04	0.04	0.08	0.13	0.02	0.04	0.03	0.05	0.05	0.03	105.33	43.99
Ldlrap1	chr4	yes	-	0.94	0.92	0.93	0.93	0.86	0.88	0.89	0.95	0.90	0.90	0.85	0.90	0.03	30.58	6.67
Gm11223	chr4	yes	-	0.05	0.00	0.01	0.01	0.00	0.02	0.01	0.01	0.02	0.01	0.03	0.02	0.01	21.76	6.97
Sfn	chr4	yes	-	0.92	0.92	0.91	0.93	0.96	0.95	0.92	0.92	0.94	0.98	0.95	0.94	0.02	28.95	13.16
Gm12715	chr4	yes	-	0.98	0.97	0.97	0.98	0.98	0.98	0.99	0.97	0.98	0.98	0.94	0.97	0.01	61.80	18.21
Impad1	chr4	yes	-	0.98	0.97	0.94	0.93	0.89	0.94	0.94	0.99	0.92	0.94	0.92	0.94	0.03	14.62	2.00
Steap4	chr5	yes	-	0.07	0.05	0.02	0.01	0.03	0.05	0.07	0.01	0.02	0.01	0.07	0.04	0.02	20.59	7.68
Gbp8	chr5	yes	-	0.04	0.04	0.03	0.02	0.01	0.01	0.01	0.02	0.03	0.02	0.01	0.02	0.01	57.42	13.06
Acacb	chr5	yes	-	-	-	0.01	-	-	0.01	0.08	0.02	0.11	0.11	0.12	0.07	0.05	11.62	1.46
Stbd1	chr5	yes	-	0.01	0.01	0.02	0.00	0.00	0.00	0.01	0.01	0.00	0.01	0.00	0.01	0.01	26.98	6.17
Alox5ap	chr5	yes	-	0.05	0.02	0.08	0.05	0.03	0.05	0.04	0.04	0.07	0.04	0.04	0.05	0.02	26.24	8.41
Myo1h	chr5	yes	-	0.01	0.06	0.02	0.02	0.06	0.02	0.02	0.03	0.06	0.06	0.02	0.03	0.02	13.77	2.10
Gm10874	chr5	yes	-	0.01	0.00	0.00	0.00	-	0.01	0.00	0.01	-	0.01	-	0.01	0.00	13.59	3.34
Gm15710	chr5	yes	-	0.02	0.02	0.06	0.00	-	0.09	0.00	0.03	-	0.00	0.08	0.03	0.04	34.06	13.11
Mir5105	chr5	yes	-	0.00	0.00	0.00	0.00	0.00	0.00	0.00	0.00	0.00	0.00	0.00	0.00	0.00	313.78	178.10
Clec2h	chr6	yes	-	0.00	0.00	0.00	0.00	0.00	0.00	0.00	0.00	0.00	0.00	0.00	0.00	0.00	294.60	62.94
Clec4a3	chr6	yes	-	-	0.06	0.05	0.05	-	-	0.10	-	0.11	-	0.06	0.07	0.03	13.20	2.60
Clec12a	chr6	yes	-	1.00	0.99	0.99	0.99	0.97	0.97	0.98	0.99	0.96	0.98	0.93	0.98	0.02	15.21	2.71
Gm20730	chr6	yes	-	0.09	0.05	0.05	0.03	0.02	0.03	0.04	0.04	0.03	0.04	0.05	0.04	0.02	52.67	21.41
Igkv4-91	chr6	yes	-	0.97	0.96	0.97	0.93	0.95	0.94	0.96	0.94	0.92	0.94	0.94	0.95	0.02	17.22	4.63
Dynlt1-ps1	chr6	yes	-	-	0.00	0.00	0.01	-	0.02	0.00	0.00	0.00	0.04	-	0.01	0.02	13.93	2.66
Apoe	chr7	yes	-	0.97	0.98	0.98	0.97	0.98	0.97	0.97	0.98	0.96	0.97	0.97	0.97	0.01	107.02	46.32
Gprc5b	chr7	yes	-	0.01	0.03	0.03	0.02	0.01	0.01	0.02	-	-	-	-	0.02	0.01	17.15	5.23
Pglyrp1	chr7	yes	-	0.93	0.97	0.97	0.98	0.92	0.98	0.91	0.99	0.96	0.96	0.96	0.96	0.03	23.61	6.66
Lilra6	chr7	yes	-	-	0.93	0.93	0.90	0.87	0.92	0.94	0.95	0.87	0.94	0.89	0.91	0.03	19.78	4.45

Capn5	chr7	yes	-	-	0.95	0.95	0.96	0.92	0.96	0.86	0.91	0.93	0.89	0.93	0.93	0.03	14.69	4.79
Gvin1	chr7	yes	-	0.05	0.04	0.02	0.03	0.00	0.02	0.03	0.01	0.01	-	0.09	0.03	0.03	16.73	5.17
Insc	chr7	yes	-	0.00	0.00	0.00	0.00	0.00	0.00	0.00	0.00	0.03	0.03	0.11	0.02	0.03	17.41	3.63
Trim30d	chr7	yes	-	0.99	0.99	0.98	0.98	0.98	0.98	0.99	0.99	0.95	0.95	0.93	0.97	0.02	19.19	2.47
Trim12a	chr7	yes	-	0.98	0.96	0.98	0.96	0.92	0.97	0.99	0.99	0.96	0.99	0.97	0.97	0.02	64.18	9.89
Fam169b	chr7	yes	-	0.91	0.85	0.87	0.94	0.88	0.88	0.87	0.87	0.86	0.92	0.95	0.89	0.03	64.05	15.16
Mir5102	chr7	yes	-	0.00	0.00	0.00	0.00	0.00	0.00	0.00	0.00	0.00	0.00	0.00	0.00	0.00	142.56	66.93
Gm7901	chr8	yes	-	0.00	0.00	-	0.00	0.01	0.01	0.00	0.00	-	0.00	0.02	0.01	0.01	13.29	3.10
Ip6k2	chr9	yes	-	0.09	0.07	0.05	0.02	0.06	0.07	0.05	0.03	0.10	0.01	0.04	0.05	0.03	31.60	4.00
Lars2	chr9	yes	-	0.06	0.10	0.10	0.08	0.10	0.09	0.09	0.08	0.07	0.09	0.07	0.09	0.01	4769.05	1573.31
Rpl29	chr9	yes	-	0.88	0.96	0.96	0.89	0.92	0.93	0.96	0.95	0.91	0.89	0.94	0.93	0.03	99.00	27.05
Gm10720	chr9	yes	-	-	0.00	0.02	-	0.01	0.01	-	0.01	0.00	-	0.01	0.01	0.01	38.25	26.99
Gm10722	chr9	yes	-	-	0.09	0.10	-	0.09	0.09	0.12	0.10	0.10	0.11	0.09	0.10	0.01	303.50	258.29
Bcl2a1a	chr9	yes	-	0.07	0.01	0.01	0.00	0.01	0.01	0.01	0.00	0.02	0.02	0.04	0.02	0.02	29.98	7.15
Gm10718	chr9	yes	-	-	0.01	0.01	-	0.02	0.04	0.02	0.00	0.02	0.03	0.02	0.02	0.01	275.95	230.59
Gm10719	chr9	yes	-	-	0.01	0.01	-	0.02	0.01	0.01	0.02	0.02	0.01	0.02	0.01	0.00	75.49	61.02
Gm10717	chr9	yes	-	-	0.03	0.03	-	0.02	0.03	0.03	0.03	0.03	0.03	0.03	0.03	0.00	495.87	416.14
AC131780.1	chr9	yes	-	-	0.01	0.02	-	0.02	0.02	0.02	0.03	0.02	0.02	0.03	0.02	0.00	78.49	67.10
Gm11168	chr9	yes	-	-	0.03	0.02	-	0.03	0.03	0.03	0.03	0.02	0.03	0.02	0.03	0.00	139.24	117.04
Gm17535	chr9	yes	-	-	0.01	0.02	-	0.02	0.02	-	0.03	0.02	-	0.02	0.02	0.00	36.71	26.63
Gstt3	chr10	yes	-	0.05	0.10	0.05	0.02	0.11	0.06	0.02	0.04	-	0.06	0.14	0.06	0.04	14.89	2.07
Rhobtb1	chr10	yes	-	0.02	0.05	0.02	0.01	0.02	0.02	0.03	0.01	0.04	0.06	0.10	0.03	0.03	43.92	5.97
Mtap7	chr10	yes	-	0.94	0.97	1.00	1.00	1.00	-	-	0.98	1.00	-	-	0.98	0.02	16.00	2.88
Tspan8	chr10	yes	-	0.00	-	-	0.00	0.01	0.01	0.00	0.00	0.01	-	-	0.00	0.01	16.28	8.94
Gm9855	chr10	yes	-	0.01	0.05	0.01	0.00	-	0.03	0.01	0.02	-	0.02	-	0.02	0.02	13.14	1.99

Grm6	chr11	yes	-	0.06	0.04	0.03	0.03	0.04	0.04	0.04	0.03	0.03	0.03	0.02	0.03	0.01	25.44	5.30
Arhgap27	chr11	yes	-	0.93	0.95	0.94	0.95	0.90	0.94	0.93	0.94	0.95	0.96	0.95	0.94	0.01	86.25	18.85
Havcr1	chr11	yes	-	0.09	0.11	0.06	0.06	0.07	0.07	0.05	0.10	0.03	0.06	0.05	0.07	0.02	17.52	2.98
Gm12185	chr11	yes	-	-	0.03	0.03	0.12	-	-	0.03	0.06	0.12	0.05	-	0.06	0.04	14.29	2.66
Gm5431	chr11	yes	-	0.08	0.09	0.13	0.08	0.08	0.11	0.10	0.08	0.14	0.10	0.15	0.10	0.03	29.21	5.81
Gm11428	chr11	yes	-	-	1.00	1.00	-	0.99	1.00	1.00	1.00	-	-	-	1.00	0.00	13.92	5.39
9930111J21Rik1	chr11	yes	-	1.00	1.00	1.00	1.00	1.00	1.00	1.00	1.00	1.00	1.00	1.00	1.00	0.00	91.04	23.50
Gm12355	chr11	yes	-	0.00	0.00	0.00	0.00	0.00	0.00	0.00	0.00	0.00	0.01	0.00	0.00	0.00	16.00	4.57
Hmga1-rs1	chr11	yes	-	0.01	0.00	0.00	0.00	0.00	0.00	0.00	0.00	0.00	0.00	0.01	0.00	0.00	24.46	12.21
Fau-ps2	chr11	yes	-	-	0.11	0.06	-	0.04	0.07	-	-	0.02	0.05	0.02	0.05	0.03	34.60	17.87
2610035D17Rik	chr11	yes	-	1.00	1.00	1.00	1.00	1.00	1.00	1.00	1.00	0.99	-	0.99	1.00	0.00	35.28	11.03
Itpk1	chr12	yes	-	0.92	0.95	0.95	0.92	0.90	0.94	0.95	0.96	-	0.94	0.94	0.94	0.02	15.62	3.64
AC073565.1	chr12	yes	-	-	0.07	0.05	0.04	0.12	0.07	0.06	0.04	0.04	0.06	0.05	0.06	0.02	27.18	8.57
AC160990.6	chr12	yes	-	-	0.06	0.06	0.04	0.07	-	0.05	0.03	-	0.04	0.06	0.05	0.01	13.17	2.35
Scamp1	chr13	yes	-	0.13	0.06	0.04	0.03	-	0.08	0.04	0.10	0.15	0.05	0.05	0.07	0.04	14.81	2.33
mmu-mir-2134-4	chr13	yes	-	0.00	0.00	0.00	-	0.00	0.00	0.00	0.00	0.00	0.00	0.00	0.00	0.00	128.09	100.87
Gm9800	chr14	yes	-	0.08	0.05	0.05	0.05	0.07	0.05	0.05	0.04	0.03	0.04	0.07	0.05	0.02	52.77	14.85
Lgals3	chr14	yes	-	-	0.95	-	0.95	0.99	0.95	0.97	0.93	-	-	-	0.96	0.02	15.45	4.66
Gm10076	chr14	yes	-	0.00	0.00	0.00	0.00	0.00	0.00	0.00	0.00	0.00	0.00	0.00	0.00	0.00	78.84	21.72
AC242409.1	chr14	yes	-	-	0.01	0.02	-	0.01	0.01	0.01	0.01	0.01	0.01	0.01	0.01	0.00	1802.84	1412.14
Ly6e	chr15	yes	-	0.94	0.95	0.97	0.96	0.97	0.98	0.97	0.96	0.96	0.95	0.98	0.96	0.01	1998.51	544.30
Ly6a	chr15	yes	-	0.97	0.99	0.99	0.99	0.99	1.00	1.00	0.99	0.99	0.98	1.00	0.99	0.01	264.51	97.88
Sdf2l1	chr16	yes	-	0.98	0.96	0.96	0.99	0.91	0.96	0.97	0.96	0.95	0.98	0.99	0.96	0.02	31.13	15.07
Mx2	chr16	yes	-	0.04	0.06	0.05	0.03	0.10	0.04	0.07	0.04	0.03	0.05	0.06	0.05	0.02	46.56	21.27
Pcp4	chr16	yes	-	-	0.09	0.12	-	0.10	0.12	0.13	-	0.14	-	0.15	0.12	0.02	16.33	6.40

H2-Q4	chr17	yes	-	0.02	0.04	0.04	0.01	0.03	0.04	0.04	0.01	0.03	0.02	0.02	0.03	0.01	213.75	48.73
H2-Ea-ps	chr17	yes	-	0.00	0.00	0.00	0.00	0.00	0.00	0.00	0.00	0.00	0.00	0.00	0.00	0.00	1529.94	258.23
Decr2	chr17	yes	-	0.97	0.89	-	1.00	0.92	-	-	0.94	-	0.96	-	0.95	0.04	11.70	1.09
H2-Q10	chr17	yes	-	0.00	0.01	0.00	0.00	0.00	0.00	0.00	0.00	0.00	0.01	0.00	0.00	0.00	50.80	9.51
Gm11127	chr17	yes	-	-	0.94	0.91	0.92	0.97	0.92	0.91	0.89	0.92	0.90	0.93	0.92	0.02	25.66	5.65
H2-Q2	chr17	yes	-	-	0.05	0.00	0.00	-	0.00	0.05	0.00	-	0.10	-	0.03	0.04	28.56	8.50
D17H6S56E-5	chr17	yes	-	0.99	0.99	0.98	0.99	0.99	0.99	0.98	0.99	0.98	0.99	0.99	0.99	0.00	614.17	129.62
Rnaset2a	chr17	yes	-	1.00	0.99	1.00	0.99	1.00	1.00	1.00	1.00	0.98	1.00	1.00	1.00	0.01	23.76	5.11
Rnf125	chr18	yes	-	0.06	0.02	0.10	0.05	0.06	0.06	0.07	0.04	0.05	0.03	0.06	0.06	0.02	22.10	5.89
Osbpl1a	chr18	yes	-	0.01	0.04	0.04	0.02	0.06	0.04	0.03	0.06	0.10	0.04	0.09	0.05	0.03	22.08	5.77
Gm5506	chr18	yes	-	1.00	1.00	1.00	1.00	1.00	1.00	1.00	1.00	0.99	0.99	0.98	1.00	0.00	68.35	28.15
4930481A15Rik	chr19	yes	-	-	-	-	-	0.97	0.98	1.00	1.00	-	1.00	0.99	0.99	0.01	13.24	1.95
Zrsr1*	**chr11**	**yes**	**yes**	**0.00**	**0.02**	**0.00**	**0.00**	**0.00**	**0.01**	**0.00**	**0.00**	**0.00**	**0.01**	**0.01**	**0.01**	**0.00**	**49.26**	**14.26**
Igf2r*	**chr17**	**yes**	**yes**	-	**0.97**	**1.00**	-	**1.00**	**0.97**	**0.94**	**0.97**	**0.92**	**0.97**	**0.87**	**0.96**	**0.04**	**18.65**	**5.97**
Eef1b2	chr1	no	yes	0.45	0.40	0.46	0.49	0.47	0.43	0.46	0.50	0.46	0.48	0.48	0.46	0.03	111.83	26.24
Stx6	chr1	no	yes	0.49	0.49	0.52	0.46	0.46	0.52	0.50	0.44	0.49	0.47	0.41	0.48	0.03	74.91	14.47
Mr1	chr1	no	yes	0.60	0.51	0.51	0.49	0.54	0.51	0.52	0.50	0.56	0.46	0.40	0.51	0.05	15.15	3.74
Bcl2l1	chr2	no	yes	-	0.36	-	-	-	-	-	-	-	0.60	-	0.48	0.17	10.77	0.49
H13	chr2	no	yes	0.50	0.50	0.45	0.45	0.46	0.43	0.44	0.44	0.47	0.44	0.48	0.46	0.03	153.46	38.25
Rbms1	chr2	no	yes	0.48	0.55	0.52	0.56	0.56	0.54	0.52	0.51	0.59	0.54	0.55	0.54	0.03	112.54	25.06
Stx16	chr2	no	yes	0.37	0.54	0.55	0.46	0.53	0.49	0.48	0.53	0.53	0.56	0.52	0.51	0.05	37.78	8.41
Gnas	chr2	no	yes	0.24	0.37	0.44	-	0.30	0.32	0.38	-	-	-	0.30	0.34	0.06	32.03	45.22
Zfp64	chr2	no	yes	0.47	0.56	0.54	0.49	0.58	0.52	0.53	0.56	0.54	0.55	0.28	0.51	0.08	23.72	5.30
Npepl1	chr2	no	yes	0.48	0.52	0.52	0.52	0.50	0.50	0.50	0.51	0.49	0.54	0.48	0.51	0.02	155.37	28.31
Blcap	chr2	no	yes	0.68	0.54	0.47	0.55	0.63	0.52	0.61	0.51	0.28	0.54	0.55	0.53	0.10	27.23	6.29

Casd1	chr6	no	yes	0.53	0.53	0.57	0.53	0.49	0.50	0.47	0.50	0.48	0.52	0.57	0.52	0.03	70.24	13.51
Copg2	chr6	no	yes	0.51	0.46	0.51	0.47	0.53	0.49	0.46	0.51	0.52	0.46	0.46	0.49	0.03	98.66	20.32
Klhdc10	chr6	no	yes	0.71	0.56	0.47	0.33	0.59	0.49	0.53	0.45	0.52	0.48	0.61	0.52	0.10	19.02	2.39
Herc3	chr6	no	yes	0.52	0.51	0.48	0.45	0.45	0.40	0.47	0.43	0.45	0.42	0.48	0.46	0.04	66.47	18.52
Etv6	chr6	no	yes	0.31	0.44	0.37	0.34	0.40	0.41	0.38	0.37	0.46	0.41	0.25	0.38	0.06	30.32	4.70
Ampd3	chr7	no	yes	0.52	0.49	0.53	0.49	0.50	0.54	0.56	0.58	0.57	0.52	0.50	0.53	0.03	32.88	4.09
Lsp1	chr7	no	yes	0.47	0.48	0.49	0.49	0.49	0.48	0.50	0.49	0.49	0.49	0.47	0.48	0.01	917.63	208.03
Ube3a	chr7	no	yes	0.44	0.51	0.49	0.50	0.48	0.49	0.53	0.51	0.53	0.50	0.54	0.50	0.03	152.05	30.48
Cd81	chr7	no	yes	0.52	0.50	0.50	0.52	0.48	0.48	0.48	0.51	0.50	0.52	0.52	0.50	0.02	480.41	162.64
Inpp5f	chr7	no	yes	0.55	0.53	0.55	0.53	0.56	0.56	0.53	0.57	0.58	0.52	0.56	0.55	0.02	94.37	13.30
Tnfrsf26	chr7	no	yes	0.61	0.62	0.64	0.57	0.64	0.63	0.59	0.61	0.63	0.61	0.52	0.61	0.04	23.80	3.80
Tssc4	chr7	no	yes	0.49	0.51	0.46	0.50	0.54	0.50	0.50	0.51	0.44	0.53	0.49	0.50	0.03	48.60	13.40
Dhcr7	chr7	no	yes	0.40	0.33	0.34	0.33	0.38	0.43	0.36	0.33	0.28	0.36	0.29	0.35	0.04	51.02	9.80
Nap1l4	chr7	no	yes	0.50	0.50	0.50	0.50	0.50	0.49	0.49	0.47	0.49	0.47	0.49	0.49	0.01	226.30	52.67
Arrdc2	chr8	no	yes	0.48	0.47	0.46	0.53	0.46	0.47	0.42	0.52	0.43	0.53	0.49	0.48	0.04	27.46	6.70
Tle3	chr9	no	yes	0.75	0.79	0.73	0.78	0.72	0.75	0.73	0.69	0.77	0.74	0.71	0.74	0.03	56.60	15.73
Ctsh	chr9	no	yes	0.53	0.55	0.57	0.56	0.55	0.53	0.55	0.56	0.55	0.54	0.53	0.55	0.01	511.41	83.16
Snx14	chr9	no	yes	0.38	0.37	0.40	0.42	0.43	0.38	0.44	0.44	0.40	0.42	0.35	0.40	0.03	43.49	11.38
Gramd1b	chr9	no	yes	0.29	0.32	0.27	0.24	-	0.26	0.38	0.23	0.27	0.18	0.17	0.26	0.06	13.31	2.44
*Commd1**	chr11	no	yes	0.59	0.60	0.59	0.46	0.57	0.48	0.58	0.50	0.56	0.47	0.42	0.53	0.06	47.42	6.24
B3gnt2	chr11	no	yes	-	-	-	-	-	-	-	0.53	-	0.44	-	0.48	0.06	13.16	2.21
Ppp2r5c	chr12	no	yes	0.51	0.52	0.52	0.52	0.51	0.53	0.53	0.52	0.54	0.51	0.57	0.53	0.02	147.42	23.19
Dync1h1	chr12	no	yes	0.50	0.50	0.50	0.56	0.50	0.48	0.52	0.53	0.45	0.52	0.50	0.51	0.03	78.09	19.17
Wars	chr12	no	yes	0.47	0.49	0.46	0.52	0.52	0.49	0.47	0.47	0.51	0.48	0.48	0.49	0.02	123.94	26.55
E2f3	chr13	no	yes	-	-	-	-	-	0.57	-	-	-	-	-	0.57	-	10.37	-

Pde4d	chr13	no	yes	0.54	0.55	0.63	-	-	-	-	0.58	0.43	0.59	-	0.55	0.07	15.15	4.74
Mbnl2	chr14	no	yes	0.43	0.47	0.51	0.50	0.58	0.49	0.49	0.46	0.49	0.45	0.46	0.48	0.04	109.62	9.66
Slc38a2	chr15	no	yes	0.62	0.67	0.67	0.65	0.64	0.66	0.66	0.72	0.71	0.67	0.68	0.67	0.03	153.67	34.35
Slc38a1	chr15	no	yes	0.69	0.65	0.59	0.66	0.64	0.64	0.68	0.65	0.63	0.67	0.67	0.65	0.03	150.45	18.18
Eif2c2	chr15	no	yes	0.42	0.47	0.48	0.41	0.46	0.43	0.48	0.46	0.50	0.50	0.50	0.46	0.03	128.45	16.01
Galnt6	chr15	no	yes	0.59	0.60	0.64	0.63	0.61	0.58	0.63	0.64	0.64	0.67	0.65	0.62	0.03	48.46	16.48
Trappc9	chr15	no	yes	0.51	0.53	0.56	0.52	0.54	0.54	0.59	0.49	0.56	0.57	0.45	0.53	0.04	87.17	15.57
Chrac1	chr15	no	yes	0.45	0.50	0.48	0.49	0.52	0.47	0.54	0.45	0.53	0.51	0.55	0.50	0.04	75.88	15.41
Bbx	chr16	no	yes	0.45	0.46	0.43	0.47	0.47	0.45	0.45	0.44	0.38	0.42	0.46	0.44	0.03	104.36	11.07
Runx1	chr16	no	yes	0.62	0.55	0.55	0.61	0.55	0.55	0.59	0.61	0.54	0.55	0.56	0.57	0.03	70.18	9.61
Qk	chr17	no	yes	0.47	0.58	0.54	0.56	0.49	0.56	0.55	0.58	0.54	0.46	0.52	0.53	0.04	165.04	22.39
Arid1b	chr17	no	yes	0.58	0.55	0.55	0.54	0.53	0.59	0.56	0.53	0.55	0.51	0.58	0.55	0.02	148.73	30.14
*Impact**	chr18	no	yes	0.11	0.12	0.12	0.04	0.12	0.03	0.14	0.10	0.16	0.10	0.26	0.12	0.06	23.19	7.53
Tbc1d12	chr19	no	yes	0.49	-	-	-	-	-	-	-	-	-	-	0.49	-	11.73	-
Gm16299	chr19	no	yes	-	-	-	-	-	-	-	-	-	-	0.38	0.38	-	16.92	-
Xlr4b	chrX	no	yes	0.44	0.76	0.75	0.41	-	-	0.95	0.21	0.33	-	0.01	0.49	0.32	23.60	9.47
Xist	chrX	no	yes	0.69	0.47	0.48	0.79	0.10	0.09	0.07	0.81	0.65	0.96	0.99	0.55	0.34	147.62	46.74
																**mean**	**137.37**	**49.16**

T cells

				AI							Abundance	
**Gene**	**Chr**	**Biased**	**Imprinted**	**Control**	**E13.1**	**E13.2**	**E13.24**	**E13.29**	**Mean(AI)**	**Std(AI)**	**Mean**	**Std**
Slc41a1	chr1	yes	-	0.13	0.13	0.13	0.12	0.12	0.13	0.01	159.17	27.98
Ica1l	chr1	yes	-	0.04	0.07	0.05	0.05	0.05	0.05	0.01	93.14	13.99
Chst10	chr1	yes	-	0.89	0.91	0.89	0.92	0.91	0.90	0.01	58.83	10.24

A630001G21Rik	chr1	yes	-	0.95	0.91	0.92	0.94	0.92	0.93	0.01	16.10	2.25
Gm10075	chr1	yes	-	0.88	0.90	0.89	0.90	0.88	0.89	0.01	56.92	25.13
Slc30a4	chr2	yes	-	0.97	0.98	0.94	0.96	0.98	0.97	0.02	27.91	11.33
Sqrdl	chr2	yes	-	1.00	1.00	0.99	0.98	0.99	0.99	0.01	28.84	7.37
Serinc3	chr2	yes	-	0.11	0.13	0.10	0.10	0.09	0.10	0.01	2287.71	472.72
Eya2	chr2	yes	-	0.95	0.97	0.93	0.90	0.98	0.95	0.03	15.14	2.20
Lypd6b	chr2	yes	-	1.00	1.00	0.99	1.00	1.00	1.00	0.00	26.13	2.72
Ltk	chr2	yes	-	-	0.00	0.06	0.02	0.02	0.03	0.03	12.02	0.88
Pkig	chr2	yes	-	0.04	0.04	0.05	0.04	0.04	0.04	0.00	200.04	112.50
Ldlrad3	chr2	yes	-	0.92	0.95	0.96	0.95	0.95	0.95	0.01	75.77	17.22
Rapgef4	chr2	yes	-	0.02	0.01	0.03	0.02	0.01	0.02	0.01	38.93	9.36
Gm13699	chr2	yes	-	1.00	1.00	0.99	0.99	0.99	0.99	0.00	41.56	22.89
D430041D05Rik	chr2	yes	-	0.02	0.01	0.02	0.02	0.03	0.02	0.01	98.80	30.47
Gm13654	chr2	yes	-	0.98	0.99	0.99	0.98	0.97	0.98	0.01	19.98	8.29
Gm13736	chr2	yes	-	0.00	-	0.00	0.06	0.00	0.01	0.03	18.91	8.85
Mir5115	chr2	yes	-	0.00	0.00	0.00	0.00	0.00	0.00	0.00	196.70	80.98
Serpini1	chr3	yes	-	0.04	0.08	0.05	0.11	0.07	0.07	0.03	74.54	21.42
Ugt8a	chr3	yes	-	0.00	-	0.07	0.05	0.02	0.04	0.03	22.81	4.81
Cnn3	chr3	yes	-	0.92	0.94	0.94	0.94	0.96	0.94	0.01	199.41	36.28
Ptgr1	chr4	yes	-	0.12	0.13	0.13	0.10	0.12	0.12	0.01	51.67	9.07
Slc5a9	chr4	yes	-	0.96	0.98	0.95	0.97	0.95	0.96	0.01	16.36	3.16
Spata6	chr4	yes	-	0.93	0.90	0.90	0.86	0.89	0.89	0.02	65.59	7.33
2010015L04Rik	chr4	yes	-	0.91	0.92	0.85	0.86	0.87	0.88	0.03	66.96	8.51
Tnfrsf14	chr4	yes	-	0.07	0.14	0.15	0.11	0.10	0.11	0.03	13.73	2.69
Ubxn10	chr4	yes	-	0.02	0.02	0.02	0.06	0.03	0.03	0.02	22.41	2.61

Gm11223	chr4	yes	-	0.03	0.03	0.02	0.02	0.02	0.02	0.01	274.90	59.44
Tmem245	chr4	yes	-	0.89	0.85	0.88	0.87	0.88	0.88	0.02	59.50	20.27
Gm12715	chr4	yes	-	0.98	0.98	0.99	0.99	0.97	0.98	0.01	43.82	8.38
Impad1	chr4	yes	-	0.95	0.89	0.90	0.88	0.87	0.90	0.03	37.78	10.39
Gm12372	chr4	yes	-	0.00	-	0.01	0.04	0.00	0.01	0.02	18.85	2.02
Crmp1	chr5	yes	-	0.06	0.04	0.04	0.03	0.03	0.04	0.01	132.65	23.10
Acad12	chr5	yes	-	0.01	0.02	0.04	0.08	0.02	0.04	0.03	22.37	2.27
Cldn4	chr5	yes	-	0.13	0.15	0.10	0.08	0.09	0.11	0.03	72.85	27.50
Zfp498	chr5	yes	-	0.05	0.08	0.07	0.11	0.10	0.08	0.02	11.94	2.29
Ccdc164	chr5	yes	-	0.10	0.08	0.12	0.07	0.08	0.09	0.02	135.51	26.84
Mir5105	chr5	yes	-	0.00	0.00	0.00	0.00	0.00	0.00	0.00	356.07	161.94
Plekha8	chr6	yes	-	0.00	0.01	0.05	0.02	0.04	0.02	0.02	21.32	5.88
Clstn3	chr6	yes	-	0.00	0.01	0.03	0.02	0.01	0.01	0.01	21.45	4.25
Gimap8	chr6	yes	-	0.95	0.87	0.94	0.93	0.91	0.92	0.03	39.72	6.51
Lpar5	chr6	yes	-	0.05	0.06	0.07	0.05	0.06	0.06	0.01	13.87	2.77
Trbv21	chr6	yes	-	0.01	0.01	0.01	0.01	0.01	0.01	0.00	37.43	6.57
Dynlt1-ps1	chr6	yes	-	0.00	-	0.00	0.00	0.00	0.00	0.00	29.11	9.16
Apoe	chr7	yes	-	0.95	0.98	0.90	0.94	0.97	0.95	0.03	24.59	8.53
Capn5	chr7	yes	-	0.05	0.07	0.09	0.07	0.09	0.07	0.02	169.72	44.46
Capn12	chr7	yes	-	-	0.01	0.01	0.03	0.00	0.02	0.01	14.92	3.14
Trim12a	chr7	yes	-	0.92	0.95	0.98	0.96	0.97	0.96	0.02	49.85	7.66
Plekhf1	chr7	yes	-	0.11	0.10	0.10	0.11	0.12	0.11	0.01	107.04	36.15
Mir5102	chr7	yes	-	0.00	0.00	0.00	0.00	0.00	0.00	0.00	155.10	16.95
Lass4	chr8	yes	-	0.06	0.07	0.12	0.07	0.05	0.07	0.03	20.44	1.84
Rab11fip1	chr8	yes	-	0.93	-	0.88	0.89	0.91	0.90	0.02	14.06	1.79

Nwd1	chr8	yes	-	-	0.99	0.96	0.97	0.98	0.98	0.01	32.60	14.59
Lig4	chr8	yes	-	0.93	0.92	0.93	0.93	0.93	0.93	0.01	78.18	18.31
Gm10282	chr8	yes	-	0.09	0.04	0.06	0.08	0.08	0.07	0.02	54.31	13.17
Slc37a2	chr9	yes	-	0.90	0.92	0.94	0.91	0.94	0.92	0.02	46.03	5.23
Arpp21	chr9	yes	-	0.91	0.89	0.91	0.89	0.89	0.90	0.01	590.87	66.46
Ip6k2	chr9	yes	-	0.04	0.08	0.08	0.06	0.06	0.07	0.02	98.49	28.55
Lars2	chr9	yes	-	0.07	0.15	0.10	0.09	0.11	0.10	0.03	5596.21	486.73
Ncam1	chr9	yes	-	0.04	0.03	0.03	-	0.03	0.03	0.00	27.51	20.57
Slc35f2	chr9	yes	-	0.06	0.07	0.06	0.11	0.10	0.08	0.02	52.46	3.76
Rpl29	chr9	yes	-	0.87	0.93	0.96	0.86	0.88	0.90	0.04	92.68	25.55
Mmp13	chr9	yes	-	0.01	-	0.00	0.03	0.00	0.01	0.01	15.18	3.71
Pcbp3	chr10	yes	-	0.08	0.06	0.06	0.06	0.04	0.06	0.01	200.81	60.52
Perp	chr10	yes	-	0.01	0.01	0.00	0.00	0.00	0.00	0.00	20.42	4.97
Slc17a8	chr10	yes	-	0.00	-	0.00	0.02	0.00	0.01	0.01	16.07	4.89
Akap12	chr10	yes	-	0.97	0.97	0.97	0.98	0.98	0.97	0.00	45.49	9.65
Lyz2	chr10	yes	-	0.09	0.03	0.03	0.03	0.09	0.05	0.03	38.75	11.67
P4ha2	chr11	yes	-	0.00	0.03	0.02	0.03	0.01	0.02	0.01	15.45	3.41
Il9r	chr11	yes	-	0.13	0.10	0.08	0.08	0.06	0.09	0.02	20.33	6.78
Cacng4	chr11	yes	-	0.09	0.06	0.00	0.08	0.05	0.06	0.03	39.10	9.33
Ntn1	chr11	yes	-	0.91	0.96	0.89	0.91	0.88	0.91	0.03	19.86	5.28
Arhgap27	chr11	yes	-	0.89	0.89	0.91	0.94	0.92	0.91	0.02	45.40	9.11
Slit3	chr11	yes	-	0.08	0.04	0.03	0.10	0.02	0.05	0.03	30.59	14.82
Gm12355	chr11	yes	-	0.00	0.00	0.00	0.00	0.00	0.00	0.00	30.80	8.11
Hmga1-rs1	chr11	yes	-	0.00	0.00	0.00	0.00	0.00	0.00	0.00	35.95	14.22
2610035D17Rik	chr11	yes	-	1.00	1.00	0.99	1.00	1.00	1.00	0.00	28.46	6.97

Srp54a	chr12	yes	-	0.85	-	1.00	0.93	0.98	0.94	0.07	11.61	1.75
Map4k5	chr12	yes	-	0.92	0.90	0.87	0.89	0.91	0.90	0.02	51.42	12.59
Greb1	chr12	yes	-	0.08	0.11	0.08	0.10	0.08	0.09	0.02	35.51	13.28
Fam177a	chr12	yes	-	0.02	0.07	0.12	0.08	0.07	0.07	0.03	11.91	0.86
Cap2	chr13	yes	-	0.09	0.08	0.08	0.09	0.13	0.10	0.02	13.90	3.52
Ctsl	chr13	yes	-	0.08	0.08	0.08	0.09	0.09	0.09	0.00	1196.91	252.12
Gzma	chr13	yes	-	0.04	0.06	0.05	0.04	0.05	0.05	0.01	258.18	35.25
Cdc14b	chr13	yes	-	0.06	0.09	0.09	0.09	0.07	0.08	0.01	64.69	11.97
Tubb2b	chr13	yes	-	0.95	0.99	0.98	0.97	0.97	0.97	0.01	56.25	9.22
Actn2	chr13	yes	-	0.09	0.09	0.06	0.05	0.04	0.07	0.02	11.98	1.41
Tubb2a	chr13	yes	-	0.97	0.96	0.96	0.96	0.96	0.96	0.01	158.10	45.86
mmu-mir-2134-4	chr13	yes	-	0.00	0.00	0.00	0.00	0.00	0.00	0.00	121.93	50.67
Chdh	chr14	yes	-	0.96	0.95	0.96	0.94	0.95	0.95	0.01	33.60	12.49
Epsti1	chr14	yes	-	0.96	0.96	0.97	0.93	0.95	0.95	0.02	29.50	3.79
Dzip1	chr14	yes	-	0.85	0.88	0.89	0.87	0.89	0.88	0.01	59.19	6.58
Gm9800	chr14	yes	-	0.06	0.06	0.05	0.05	0.05	0.05	0.01	117.84	41.71
Gm10076	chr14	yes	-	0.01	0.00	0.00	0.00	0.00	0.00	0.00	41.03	8.26
Trav1	chr14	yes	-	0.08	0.13	0.13	0.09	0.14	0.11	0.03	21.39	10.01
Itga5	chr15	yes	-	0.91	0.97	0.89	0.89	0.89	0.91	0.04	12.58	1.50
Endou	chr15	yes	-	0.92	0.91	0.90	0.89	0.89	0.90	0.01	387.52	123.13
Ly6e	chr15	yes	-	0.98	0.98	0.97	0.98	0.97	0.98	0.00	1177.22	420.62
Prickle1	chr15	yes	-	0.98	0.99	0.96	0.98	0.98	0.98	0.01	28.64	7.53
Apol11b	chr15	yes	-	0.00	0.00	0.00	0.01	0.00	0.00	0.00	13.82	2.38
Cd96	chr16	yes	-	0.98	0.98	0.98	0.98	0.97	0.98	0.00	128.58	9.45
Cd200	chr16	yes	-	0.07	0.03	0.05	0.03	0.02	0.04	0.02	68.90	11.12

Sdf2l1	chr16	yes	-	0.99	0.94	0.93	-	0.95	0.95	0.03	20.04	11.85
Bach1	chr16	yes	-	0.11	0.09	0.11	0.11	0.10	0.10	0.01	484.93	142.50
A930003A15Rik	chr16	yes	-	1.00	1.00	1.00	0.99	1.00	1.00	0.00	29.01	5.67
Plcxd2	chr16	yes	-	0.96	0.93	0.97	0.98	0.99	0.97	0.02	53.11	9.34
Crim1	chr17	yes	-	0.10	0.13	0.07	0.07	0.11	0.09	0.03	17.41	3.50
H2-T3	chr17	yes	-	0.01	0.01	0.01	0.01	0.01	0.01	0.00	473.51	111.29
H2-Q5	chr17	yes	-	0.01	0.00	0.01	0.01	0.00	0.01	0.00	31.66	13.08
H2-Q10	chr17	yes	-	0.00	0.00	0.00	0.06	0.02	0.02	0.03	48.31	13.36
H2-Bl	chr17	yes	-	0.01	0.04	0.02	0.03	0.02	0.02	0.01	19.40	2.97
H2-T10	chr17	yes	-	0.07	0.08	0.08	0.11	0.10	0.09	0.01	434.18	74.75
H2-Q3	chr17	yes	-	0.01	0.01	0.01	0.01	0.01	0.01	0.00	106.11	45.78
Rnaset2a	chr17	yes	-	0.97	1.00	1.00	1.00	0.99	0.99	0.01	22.32	6.32
Cyb5	chr18	yes	-	0.87	0.89	0.86	0.90	0.90	0.88	0.02	323.56	77.01
Osbpl1a	chr18	yes	-	0.06	0.11	0.15	0.13	0.12	0.11	0.03	32.58	0.87
Gm5506	chr18	yes	-	1.00	1.00	1.00	0.99	1.00	1.00	0.00	53.87	29.92
Pitpnm1	chr19	yes	-	0.86	0.92	0.89	0.90	0.95	0.90	0.03	29.55	3.78
Papss2	chr19	yes	-	1.00	0.99	0.99	0.99	0.99	0.99	0.00	24.48	3.40
Nmrk1	chr19	yes	-	0.13	0.09	0.09	0.08	0.08	0.09	0.02	44.58	13.36
Pcna-ps2	chr19	yes	-	0.00	0.00	0.01	0.01	0.01	0.01	0.00	19.69	13.79
Hnrnph2	chrX	yes	-	0.86	-	0.96	0.92	1.00	0.93	0.06	167.43	30.16
Zrsr1*	**chr11**	**yes**	**Imprinted**	**0.00**	**0.00**	**0.01**	**0.01**	**0.03**	**0.01**	**0.01**	**12.96**	**2.26**
Igf2r*	**chr17**	**yes**	**Imprinted**	**0.96**	**1.00**	**0.99**	**0.98**	**1.00**	**0.98**	**0.02**	**72.77**	**36.72**
Airn*	**chr17**	**yes**	**Imprinted**	-	**0.00**	**0.01**	**0.05**	**0.00**	**0.01**	**0.02**	**14.28**	**1.59**
Cdkn1c	chr7	maybe	Imprinted	0.98	1.00	-	-	-	0.99	0.02	13.12	1.15
Gm16299	chr19	maybe	Imprinted	-	-	-	0.01	-	0.01	-	11.85	-

Eef1b2	chr1	no	Imprinted	0.49	0.51	0.48	0.51	0.50	0.50	0.01	79.40	15.84
Stx6	chr1	no	Imprinted	0.48	0.48	0.49	0.49	0.49	0.49	0.00	431.38	99.20
Mr1	chr1	no	Imprinted	0.33	0.32	0.34	0.33	0.32	0.33	0.01	291.43	43.75
Bcl2l1	chr2	no	Imprinted	0.48	0.48	0.49	0.48	0.49	0.48	0.01	599.45	98.84
Bmp7	chr2	no	Imprinted	0.39	0.45	0.38	0.41	0.44	0.41	0.03	25.43	4.63
H13	chr2	no	Imprinted	0.48	0.49	0.51	0.46	0.47	0.48	0.02	300.74	56.81
Rbms1	chr2	no	Imprinted	0.48	0.55	0.51	0.47	0.52	0.51	0.03	91.87	24.27
Tpx2	chr2	no	Imprinted	0.47	0.50	0.50	0.47	0.49	0.49	0.02	135.57	32.64
Stx16	chr2	no	Imprinted	0.59	0.55	0.54	0.52	0.51	0.54	0.03	74.25	8.49
Zfp64	chr2	no	Imprinted	0.54	0.51	0.48	0.48	0.48	0.50	0.03	52.93	9.22
Npepl1	chr2	no	Imprinted	0.60	0.57	0.55	0.54	0.56	0.56	0.02	73.55	13.99
Sfmbt2	chr2	no	Imprinted	0.72	-	0.77	0.64	0.53	0.67	0.10	11.43	0.53
Blcap	chr2	no	Imprinted	0.67	0.45	0.71	0.42	0.62	0.57	0.13	17.58	2.21
Mafb	chr2	no	Imprinted	0.55	0.54	0.52	0.62	0.49	0.54	0.05	21.42	12.51
Casd1	chr6	no	Imprinted	0.49	0.45	0.53	0.52	0.50	0.50	0.03	57.56	8.67
Copg2	chr6	no	Imprinted	0.47	0.45	0.44	0.44	0.45	0.45	0.01	77.31	21.66
Klhdc10	chr6	no	Imprinted	0.44	0.36	0.48	0.44	0.47	0.44	0.05	60.90	13.34
Herc3	chr6	no	Imprinted	0.35	0.41	0.37	0.36	0.39	0.38	0.02	71.32	22.53
Etv6	chr6	no	Imprinted	0.46	0.46	0.56	0.60	0.54	0.52	0.06	30.21	4.28
Lsp1	chr7	no	Imprinted	0.44	0.47	0.44	0.44	0.44	0.45	0.01	650.93	178.81
Ube3a	chr7	no	Imprinted	0.53	0.42	0.52	0.51	0.51	0.50	0.04	155.62	27.91
Cd81	chr7	no	Imprinted	0.42	0.42	0.43	0.44	0.44	0.43	0.01	224.34	50.12
Inpp5f	chr7	no	Imprinted	0.36	0.35	0.36	0.43	0.35	0.37	0.04	21.92	3.71
Tnfrsf26	chr7	no	Imprinted	0.73	0.71	0.72	0.70	0.72	0.71	0.01	26.71	3.42
Tssc4	chr7	no	Imprinted	0.57	0.61	0.56	0.57	0.54	0.57	0.02	55.94	23.75

Dhcr7	chr7	no	Imprinted	0.59	0.60	0.49	0.49	0.49	0.53	0.06	101.61	15.29
Nap1l4	chr7	no	Imprinted	0.48	0.47	0.46	0.48	0.45	0.47	0.01	266.64	64.05
R74862	chr7	no	Imprinted	0.46	0.39	-	0.38	-	0.41	0.04	12.06	2.29
Arrdc2	chr8	no	Imprinted	0.55	0.49	0.50	0.45	0.44	0.49	0.04	29.27	10.18
Tle3	chr9	no	Imprinted	0.71	0.71	0.73	0.73	0.77	0.73	0.02	84.37	8.03
Snx14	chr9	no	Imprinted	0.35	0.36	0.42	0.29	0.42	0.37	0.06	20.79	2.88
Gramd1b	chr9	no	Imprinted	0.25	0.24	0.27	0.26	0.30	0.26	0.02	18.93	3.81
Phactr2	chr10	no	Imprinted	-	-	-	0.34	-	0.34	-	10.32	-
*Commd1**	chr11	no	Imprinted	0.45	0.52	0.61	0.43	0.59	0.52	0.08	55.94	18.73
Actn1	chr12	no	Imprinted	0.71	0.64	0.68	0.66	0.66	0.67	0.03	87.50	15.63
Ppp2r5c	chr12	no	Imprinted	0.58	0.58	0.59	0.57	0.57	0.58	0.01	318.86	13.55
Dync1h1	chr12	no	Imprinted	0.53	0.47	0.49	0.49	0.50	0.50	0.02	81.05	16.74
Smoc1	chr12	no	Imprinted	0.67	0.67	0.69	0.53	0.64	0.64	0.07	73.06	19.38
Wars	chr12	no	Imprinted	0.45	0.47	0.50	0.49	0.45	0.47	0.02	73.17	7.26
E2f3	chr13	no	Imprinted	-	-	0.46	0.57	0.53	0.52	0.05	11.29	1.51
Pde4d	chr13	no	Imprinted	0.62	0.64	0.55	0.58	0.59	0.60	0.04	36.77	6.82
Mbnl2	chr14	no	Imprinted	0.63	0.62	0.61	0.63	0.64	0.62	0.01	142.15	18.08
Slc38a2	chr15	no	Imprinted	0.68	0.64	0.69	0.69	0.69	0.68	0.02	366.56	76.16
Slc38a1	chr15	no	Imprinted	0.54	0.56	0.47	0.49	0.50	0.51	0.04	177.38	18.60
Eif2c2	chr15	no	Imprinted	0.58	0.45	0.51	0.53	0.52	0.52	0.05	98.36	12.03
Galnt6	chr15	no	Imprinted	0.63	0.65	0.69	0.54	0.63	0.63	0.05	25.34	5.74
Trappc9	chr15	no	Imprinted	0.56	0.53	0.49	0.50	0.48	0.51	0.03	63.74	7.71
Chrac1	chr15	no	Imprinted	0.45	0.56	0.52	0.52	0.55	0.52	0.04	64.57	26.40
Bbx	chr16	no	Imprinted	0.44	0.45	0.42	0.41	0.44	0.43	0.02	88.84	15.54
Runx1	chr16	no	Imprinted	0.42	0.41	0.43	0.41	0.44	0.42	0.01	136.01	9.63

Qk	chr17	no	Imprinted	0.51	0.51	0.54	0.51	0.51	0.52	0.01	175.28	25.65
Arid1b	chr17	no	Imprinted	0.58	0.55	0.56	0.54	0.56	0.56	0.02	131.69	36.87
*Impact**	chr18	no	Imprinted	0.28	0.35	0.41	0.53	0.00	0.31	0.20	13.81	3.72
Xlr4c	chrX	no	Imprinted	0.71	-	0.99	0.96	1.00	0.91	0.14	17.94	7.80
Xlr4b	chrX	no	Imprinted	0.49	0.56	0.89	0.96	0.99	0.78	0.23	42.19	3.33
Xist	chrX	no	Imprinted	0.54	0.45	0.15	0.01	0.01	0.23	0.25	197.56	24.32
										**mean**	**143.98**	**29.19**

The list of imprinted genes was retrieved from https://www.geneimprint.com and Tucci et al., 2019.A total of 167 genes were discoverable in the annotation file used in this study (ftp://ftp.ensembl.org/pub/release-68/gtf/Mus_musculus.GRCm38.68.gtf). The "Bias" threshold was set at 15% expression for the silenced allele and inclusion criteria was "all samples with a measurement". Additionally, an exclusion criteria due to missing datapoints was applied: for B cells (total = 11 samples), a minimum number of non-missing values was N=6, and if less than 6, the gene was classified as "maybe"; for T cell (total = 5 samples), one failed sample was enough for the gene to be classified as "maybe". Of the reported imprinted genes, 55 were expressed in our B cell samples, and 62 in T cell samples. To our knowledge, there are no accounts of imprinted genes in B or T cells in the literature. Of the 55 imprinted genes expressed in B cells, only 4 have been confirmed as imprinted in a related lymphoid tissue, the spleen (asterisks [*]; Andergassen et al, 2017), while only 5 genes were imprinted in the thymus among the 62 "imprinted" genes that were also expressed in T cells (Andergassen et al, 2017). The rows in the table are ordered: first biased (yes>maybe>no), then imprinted. Red highlights genes with nonrandom bias in both B and T cells. Blue highlights the genes described in the literature as imprinted and expressed biallelically in both B and T cells in our study. Purple highlights genes with nonrandom bias in both B and T cells and described in the literature as imprinted. There are imprinted genes in the literature that in our samples do not reach the AI nonrandom bias threshold criteria (<15% expression for the silenced allele), but are biased (e.g., *Impact*, see also **Supplementary Figure 6**). The only confirmed lymphoid imprinted gene (in spleen or thymus) we studied that is not biased in our B or T cell samples is *Commd1*. The abundance mean of all analyzed genes is 115.03 ± 477.90 in B cells and 101.65 ± 218.74 in T cells (not significantly different from the abundance mean from this table by t-test). Genes with low expression levels (<10 TMM-normalized counts) and genes suspected of LOH (detected by WES) were removed from the study and this table.

Supplementary Table 3. XCI escapees identified by other studies and our study showing values of AI and expression in HSC-derived lymphocytes *in vivo* from our NGS data. Genes in bold are escapees identified in our study. Genes in red are X escapees reported in the literature that were not expressed in our samples. Genes in blue were not included in our study due to lack of SNPs to estimate allelic imbalance. The abundance mean is represented in TMM-normalized counts.

	Allelic imbalance								
	T cells		B cells						
gene	E13.2	E13.29	E13.24	E13.29	E15.10	E6.42	E6.43	Abundance mean	Xi expression
1810030O07Rik	1.00	1.00	0.98	0.96	0.98	0.01	0.03	62.94	
5530601H04Rik	**0.73**	**0.74**	**0.73**	**0.74**	**0.78**	**0.16**	**0.17**	**29.65**	**yes**
5730416F02Rik	-	-	-	-	-	-	-	0.23	
Abcb7	0.99	1.00	0.99	0.97	0.99	0.00	0.01	36.13	
Alg13	0.95	0.97	0.81	0.93	0.99	0.09	0.10	14.65	
Amot	0.42	-	0.77	-	-	0.32	0.28	1.93	
Ap1s2	0.97	1.00	0.93	0.90	0.96	0.01	0.01	30.45	
Atp7a	0.95	0.99	0.82	0.93	0.99	0.04	0.10	47.07	
AU015836	-	-	-	-	-	-	-	0.49	
AU022751	-	-	-	0.00	-	-	-	0.21	
BC022960	0.98	0.94	1.00	0.89	0.96	-	-	0.86	
Bcor	1.00	1.00	0.93	0.95	0.97	0.01	0.01	71.23	
Bgn	-	-	-	-	-	-	-	0.19	
Bhlhb9	0.99	1.00	0.97	0.92	0.97	0.02	0.01	53.61	
Bmp15	-	-	-	-	-	-	-	0.22	
Car5b	0.48	0.77	0.28	0.45	0.59	0.44	0.35	1.18	
Cfp	0.89	NA	0.96	0.96	0.99	0.12	0.01	28.36	
Col4a5	-	-	-	-	-	-	-	1.81	
Cstf2	0.99	1.00	0.94	0.95	0.97	0.00	0.01	118.51	
Ctps2	0.99	1.00	0.96	0.95	0.98	0.01	0.01	113.44	
Cybb	0.30	0.54	0.93	0.84	0.93	0.00	0.01	291.67	
Ddx3x	0.79	0.99	0.73	0.91	0.72	0.02	0.02	299.47	
Dlg3	0.97	0.99	0.94	0.91	0.98	0.01	0.08	16.77	
Dynlt3	-	-	-	-	-	-	-	54.06	
Ebp	1.00	1.00	0.91	0.90	0.97	0.01	0.01	44.00	
Eif2s3x	**0.49**	**0.52**	**0.53**	**0.56**	**0.53**	**0.25**	**0.19**	**46.76**	**yes**
Ercc6l	1.00	1.00	0.90	0.89	0.98	0.01	0.10	21.35	
F8	0.88	1.00	-	0.76	1.00	-	0.33	3.55	
Fam199x	0.99	1.00	0.98	0.97	0.94	0.06	0.05	21.38	
Fam3a	0.99	1.00	0.94	0.96	0.97	0.01	0.01	27.94	
Fam50a	0.98	0.99	0.97	0.90	0.96	0.00	0.00	48.07	
Firre	0.93	0.93	-	-	-	0.00	-	8.42	
Flna	1.00	0.99	0.96	0.94	0.96	0.01	0.01	811.48	
Fmr1	0.99	1.00	0.93	0.94	0.98	0.10	0.03	93.35	
Ftx	0.91	0.91	0.96	0.92	0.95	0.35	0.21	25.17	

Fundc2	1.00	1.00	0.97	0.92	0.98	0.01	0.00	29.13	
G530011O06Rik	0.20	0.13	0.57	0.71	0.48	0.61	0.44	1.66	
G6pdx	0.99	1.00	0.96	0.95	0.97	0.01	0.01	73.63	
Gdi1	0.99	0.98	0.93	0.94	0.95	0.01	0.01	463.40	
Gla	0.97	1.00	-	0.84	1.00	-	-	5.44	
Gnl3l	0.99	0.99	0.95	0.92	0.94	0.03	0.03	64.96	
Gpm6b	0.58	-	0.93	0.79	0.95	0.23	0.24	3.03	
Gprasp1	0.98	0.99	0.93	0.90	0.94	0.01	0.01	51.27	
Gripap1	0.99	0.99	0.96	0.95	0.97	0.01	0.02	104.11	
Gk	0.95	0.99	0.97	0.89	0.99	0.01	0.10	15.92	
Hdac6	0.94	0.98	0.95	0.92	0.95	0.05	0.12	23.25	
Hs6st2	-	-	-	-	-	-	-	1.11	
Htatsf1	0.99	1.00	0.95	0.96	0.96	0.01	0.00	75.27	
Huwe1	0.98	0.99	0.92	0.93	0.95	0.02	0.03	100.25	
Idh3g	1.00	1.00	0.95	0.94	0.97	0.01	0.00	221.82	
Ids	1.00	0.99	0.97	0.92	0.96	0.01	0.02	47.79	
Ikbkg	0.99	1.00	0.96	0.95	0.97	0.01	0.01	85.87	
Il13ra1	-	-	-	-	-	-	-	1.32	
Iqsec2	-	-	-	0.58	-	-	-	1.14	
Irak1	0.99	0.99	0.95	0.94	0.97	0.01	0.01	144.37	
Itm2a	0.99	0.99	0.92	0.92	0.97	0.05	0.04	43.54	
Jpx	-	-	-	-	-	-	-	NA	
Kdm5c	**0.60**	**0.63**	**0.62**	**0.59**	**0.61**	**0.29**	**0.28**	**175.94**	**yes**
Kdm6a	**0.55**	**0.55**	**0.66**	**0.58**	**0.58**	**0.40**	**0.39**	**145.05**	**yes**
Kif4	0.99	0.99	0.86	0.88	0.98	0.14	0.17	33.39	
Lamp2	0.98	1.00	0.93	0.95	0.94	0.01	0.02	105.10	
Maged1	0.96	1.00	0.98	0.95	0.96	-	0.00	4.83	
Magt1	0.99	0.99	0.94	0.94	0.97	0.01	0.01	70.50	
Mecp2	0.99	0.99	0.95	0.95	0.95	0.01	0.04	44.26	
Mid1	0.45	0.40	0.62	0.68	0.65	0.08	0.06	6.87	
Mid2	-	-	-	-	-	-	-	0.68	
Mmgt1	0.99	1.00	0.93	0.91	0.98	0.04	0.05	43.28	
Msn	0.99	1.00	0.94	0.94	0.97	0.01	0.01	1347.44	
Mtcp1	0.96	0.99	0.91	0.91	0.90	0.00	0.06	14.88	
Nkap	0.99	0.99	0.94	0.94	0.97	0.01	0.02	38.00	
Nudt11	-	-	-	-	-	-	-	0.08	
Ofd1	0.97	0.99	0.94	0.94	0.96	0.02	0.06	19.34	
Ogt	1.00	1.00	0.96	0.94	0.96	0.01	0.01	556.30	
Otud5	0.99	1.00	0.93	0.94	0.96	0.03	0.04	227.57	
Pbdc1	**0.65**	**0.77**	**0.75**	**0.71**	**0.80**	**0.30**	**0.23**	**53.17**	**yes**
Pcdh19	-	-	-	-	-	-	-	0.84	
Pdha1	1.00	1.00	0.93	0.96	0.96	0.01	0.02	107.61	
Pim2	0.99	0.99	0.83	0.88	0.95	0.01	0.01	55.97	
Plp1	1.00	0.97	0.89	0.92	0.99	0.23	0.12	2.59	
Pls3	-	-	-	1.00	-	-	-	2.18	

Pqbp1	0.99	0.99	0.95	0.90	0.96	0.01	0.01	104.39	
Rbbp7	-	-	-	-	-	-	-	192.46	
Rlim	0.98	1.00	0.93	0.96	0.96	0.01	0.01	84.62	
Rnf128	-	-	-	-	-	-	-	0.53	
Rps4x	0.99	1.00	0.95	0.94	0.96	0.01	0.01	196.71	
Sh3bgrl	0.99	0.99	0.93	0.94	0.96	0.01	0.04	134.77	
Shroom4	-	-	-	-	-	0.65	-	1.07	
Siah1b	0.89	0.98	0.95	0.90	0.97	0.02	0.00	7.53	
Slc16a2	-	-	-	-	-	-	-	0.50	
Slc35a2	0.99	1.00	0.96	0.96	0.97	0.02	0.02	36.33	
Slc6a8	-	-	-	-	-	-	-	0.66	
Snx12	0.99	0.99	0.98	0.95	0.97	0.23	0.19	42.85	
Ssxb3	-	-	-	-	-	-	-	0.15	
Suv39h1	0.99	1.00	0.88	0.93	0.97	0.05	0.14	62.48	
Syap1	0.99	1.00	0.99	0.96	0.97	0.01	0.00	35.80	
Syp	-	-	-	-	-	-	-	1.33	
Tab3	0.98	0.99	0.95	0.90	0.97	0.02	0.07	20.34	
Tafl	0.98	1.00	0.86	0.90	0.94	0.05	0.03	64.98	
5430427O19Rik	0.63	0.93	0.96	0.92	0.98	0.02	0.03	40.30	
Tmem164	0.99	1.00	0.88	0.90	0.96	0.01	0.02	88.06	
Tmem29	0.83	0.83	0.86	0.89	0.83	0.25	0.29	3.81	
Tmem47	-	-	-	0.20	-	-	-	0.74	
Tmsb15l	-	1.00	1.00	0.74	1.00	-	-	0.28	
Tmsb4x	1.00	1.00	0.96	0.96	0.97	0.00	0.00	2015.50	
Uba1	1.00	0.99	0.95	0.93	0.96	0.00	0.00	326.39	
Ubl4a	1.00	0.99	0.95	0.90	0.95	0.01	0.00	69.97	
Usp9x	0.94	0.99	0.95	0.92	0.95	0.05	0.04	80.36	
Utp14a	**0.99**	**0.62**	**0.94**	**0.77**	**0.77**	**0.03**	**0.22**	**26.22**	**yes**
Vbp1	1.00	1.00	0.95	0.96	0.98	0.01	0.03	121.16	
Vsig4	-	-	-	-	-	-	-	0.27	
Wdr13	0.99	1.00	0.95	0.95	0.96	0.03	0.00	43.80	
Xist	**0.01**	**0.01**	**0.10**	**0.09**	**0.07**	**0.96**	**0.99**	**162.43**	**yes**
Yipf6	0.99	0.99	0.96	0.97	0.97	0.05	0.08	43.46	
Zbtb33	1.00	-	-	-	-	-	-	5.64	
Zfp280c	0.96	0.99	0.90	0.92	0.94	0.03	0.06	26.07	
Zmym3	0.99	1.00	0.93	0.93	0.96	0.02	0.04	37.85	
Zrsr2	0.98	1.00	0.92	0.93	0.95	0.01	0.01	33.89	
Gm8822	**0.38**	**0.35**	**0.44**	**0.35**	**0.38**	**0.39**	**0.30**	**19.69**	**yes**

Supplementary Table 4. List of all statistically significant AI differences between samples for the 14 genes identified as putatively RME in B cells (related with Figure 4A). P-value associated with each comparison after applying the QCC correction to the binomial test (Mendelevich et al., 2021). The dAI is the difference between AI values. Clonality indicates whether the sample was originated upon expansion of 1, 50 or 200 transplanted cells. Shaded rows highlight comparisons between only monoclonal (shaded) or only polyclonal samples (unshaded).

Gene	chr	sample1	sample2	AI (1)	AI (2)	p-val (BT, CC)	dAI	Abundance (1)	Abundance (2)	clonality (1)	clonality (2)
Dpp7	chr2	E6.42_B	E6.43_B	0.70	0.21	9.10E-13	0.49	28.88	26.50	1 HSC	1 HSC
Dpp7	chr2	E13.29_B	E6.43_B	0.51	0.21	1.76E-06	0.30	39.69	26.50	1 HSC	1 HSC
Dpp7	chr2	E15.10_B	E6.42_B	0.46	0.70	1.45E-06	0.24	48.56	28.88	1 HSC	1 HSC
Dpp7	chr2	E15.2_B	E6.42_B	0.42	0.70	1.63E-07	0.28	55.67	28.88	200 HSCs	1 HSC
Dpp7	chr2	E13.2_B	E6.42_B	0.45	0.70	4.26E-07	0.25	42.23	28.88	200 HSCs	1 HSC
Dpp7	chr2	E6.1_B	E6.42_B	0.45	0.70	2.87E-07	0.25	56.67	28.88	50 HSCs	1 HSC
Aldh4a1	chr4	E15.10_B	E6.42_B	0.94	0.44	3.54E-31	0.50	10.42	40.17	1 HSC	1 HSC
Aldh4a1	chr4	E15.10_B	E6.43_B	0.94	0.48	1.02E-20	0.46	10.42	18.46	1 HSC	1 HSC
Aldh4a1	chr4	E13.24_B	E15.10_B	0.49	0.94	3.10E-18	0.45	13.12	10.42	1 HSC	1 HSC
Aldh4a1	chr4	E13.29_B	E15.10_B	0.58	0.94	8.23E-17	0.36	18.59	10.42	1 HSC	1 HSC
Aldh4a1	chr4	E15.2_B	E15.10_B	0.45	0.94	2.50E-27	0.49	35.58	10.42	200 HSCs	1 HSC
Aldh4a1	chr4	E15.10_B	E6.1_B	0.94	0.48	6.44E-27	0.46	10.42	34.19	1 HSC	50 HSCs
Aldh4a1	chr4	E15.10_B	E6.2_B	0.94	0.48	1.47E-19	0.46	10.42	13.83	1 HSC	50 HSCs
Aldh4a1	chr4	E13.1_B	E15.10_B	0.50	0.94	5.69E-24	0.45	22.65	10.42	200 HSCs	1 HSC
Aldh4a1	chr4	E13.2_B	E15.10_B	0.50	0.94	3.58E-24	0.44	21.88	10.42	200 HSCs	1 HSC
Gnpda2	chr5	E15.10_B	E6.42_B	0.71	0.31	2.85E-07	0.41	37.79	28.76	1 HSC	1 HSC
Gnpda2	chr5	E13.29_B	E6.42_B	0.69	0.31	5.44E-07	0.38	29.53	28.76	1 HSC	1 HSC
Gnpda2	chr5	E13.2_B	E6.42_B	0.68	0.31	7.53E-08	0.38	34.71	28.76	200 HSCs	1 HSC
Igkv6-25	chr6	E13.24_B	E15.10_B	0.72	0.34	5.66E-58	0.39	50.26	30.99	1 HSC	1 HSC
Igkv6-25	chr6	E13.24_B	E13.29_B	0.72	0.37	1.08E-44	0.35	50.26	26.51	1 HSC	1 HSC
Igkv6-25	chr6	E13.24_B	E6.42_B	0.72	0.37	1.41E-49	0.35	50.26	38.39	1 HSC	1 HSC
Igkv6-25	chr6	E13.24_B	E6.43_B	0.72	0.45	2.38E-20	0.27	50.26	26.38	1 HSC	1 HSC

Igkv6-25	chr6	E15.10_B	E6.43_B	0.34	0.45	5.44E-07	0.12	30.99	26.38	1 HSC	1 HSC
Igkv6-25	chr6	E13.24_B	E6.1_B	0.72	0.29	3.78E-74	0.43	50.26	25.65	1 HSC	50 HSCs
Igkv6-25	chr6	E15.2_B	E13.24_B	0.30	0.72	4.08E-65	0.42	39.43	50.26	200 HSCs	1 HSC
Igkv6-25	chr6	E13.2_B	E13.24_B	0.33	0.72	2.44E-60	0.39	24.48	50.26	200 HSCs	1 HSC
Igkv6-25	chr6	E13.1_B	E13.24_B	0.35	0.72	3.66E-53	0.38	25.58	50.26	200 HSCs	1 HSC
Igkv6-25	chr6	E13.24_B	E6.2_B	0.72	0.36	3.95E-36	0.36	50.26	18.01	1 HSC	50 HSCs
Igkv6-25	chr6	E6.1_B	E6.43_B	0.29	0.45	1.33E-12	0.16	25.65	26.38	50 HSCs	1 HSC
Igkv6-25	chr6	E15.2_B	E6.43_B	0.30	0.45	1.98E-10	0.15	39.43	26.38	200 HSCs	1 HSC
Igkv6-25	chr6	E13.2_B	E6.43_B	0.33	0.45	1.18E-07	0.12	24.48	26.38	200 HSCs	1 HSC
Igkv6-25	chr6	E13.29_B	E6.1_B	0.37	0.29	4.19E-06	0.08	26.51	25.65	1 HSC	50 HSCs
Igkv6-25	chr6	E6.1_B	E6.42_B	0.29	0.37	7.85E-07	0.08	25.65	38.39	50 HSCs	1 HSC
Plekha8	chr6	E15.10_B	E6.42_B	0.09	0.45	4.63E-11	0.37	13.41	13.31	1 HSC	1 HSC
Plekha8	chr6	E13.29_B	E6.42_B	0.15	0.45	1.83E-07	0.31	14.34	13.31	1 HSC	1 HSC
Plekha8	chr6	E15.10_B	E6.43_B	0.09	0.37	6.23E-07	0.29	13.41	16.40	1 HSC	1 HSC
Plekha8	chr6	E13.2_B	E6.42_B	0.20	0.45	1.72E-06	0.26	17.30	13.31	200 HSCs	1 HSC
Kdm8	chr7	E15.10_B	E6.43_B	0.03	0.55	3.53E-24	0.53	12.81	16.94	1 HSC	1 HSC
Kdm8	chr7	E13.29_B	E15.10_B	0.48	0.03	3.26E-24	0.45	24.54	12.81	1 HSC	1 HSC
Kdm8	chr7	E13.24_B	E15.10_B	0.44	0.03	1.41E-17	0.42	17.24	12.81	1 HSC	1 HSC
Kdm8	chr7	E15.10_B	E6.42_B	0.03	0.43	1.82E-20	0.40	12.81	25.94	1 HSC	1 HSC
Kdm8	chr7	E15.2_B	E15.10_B	0.56	0.03	8.42E-28	0.53	27.69	12.81	200 HSCs	1 HSC
Kdm8	chr7	E15.10_B	E6.2_B	0.03	0.53	1.35E-23	0.50	12.81	14.96	1 HSC	50 HSCs
Kdm8	chr7	E13.2_B	E15.10_B	0.41	0.03	1.01E-19	0.39	19.70	12.81	200 HSCs	1 HSC
Kdm8	chr7	E13.1_B	E15.10_B	0.41	0.03	8.32E-19	0.38	20.61	12.81	200 HSCs	1 HSC
Kdm8	chr7	E15.10_B	E6.1_B	0.03	0.39	3.98E-18	0.36	12.81	27.73	1 HSC	50 HSCs
Pkp3	chr7	E13.29_B	E6.42_B	0.94	0.12	8.75E-159	0.82	47.17	96.81	1 HSC	1 HSC
Pkp3	chr7	E13.29_B	E15.10_B	0.94	0.18	1.51E-132	0.76	47.17	79.42	1 HSC	1 HSC
Pkp3	chr7	E6.42_B	E6.43_B	0.12	0.84	5.12E-120	0.72	96.81	71.84	1 HSC	1 HSC
Pkp3	chr7	E15.10_B	E6.43_B	0.18	0.84	2.55E-96	0.66	79.42	71.84	1 HSC	1 HSC

Pkp3	chr7	E13.24_B	E13.29_B	0.43	0.94	4.60E-61	0.51	139.94	47.17	1 HSC	1 HSC
Pkp3	chr7	E13.24_B	E6.43_B	0.43	0.84	6.62E-36	0.41	139.94	71.84	1 HSC	1 HSC
Pkp3	chr7	E13.24_B	E6.42_B	0.43	0.12	1.29E-36	0.30	139.94	96.81	1 HSC	1 HSC
Pkp3	chr7	E13.24_B	E15.10_B	0.43	0.18	9.35E-23	0.25	139.94	79.42	1 HSC	1 HSC
Pkp3	chr7	E13.29_B	E6.1_B	0.94	0.32	1.01E-97	0.62	47.17	110.71	1 HSC	50 HSCs
Pkp3	chr7	E13.2_B	E13.29_B	0.39	0.94	1.91E-74	0.55	79.17	47.17	200 HSCs	1 HSC
Pkp3	chr7	E6.1_B	E6.43_B	0.32	0.84	1.66E-64	0.52	110.71	71.84	50 HSCs	1 HSC
Pkp3	chr7	E13.1_B	E13.29_B	0.44	0.94	1.19E-60	0.50	75.25	47.17	200 HSCs	1 HSC
Pkp3	chr7	E13.29_B	E6.2_B	0.94	0.47	3.69E-45	0.48	47.17	55.39	1 HSC	50 HSCs
Pkp3	chr7	E13.2_B	E6.43_B	0.39	0.84	7.31E-46	0.45	79.17	71.84	200 HSCs	1 HSC
Pkp3	chr7	E15.2_B	E6.42_B	0.56	0.12	3.95E-65	0.43	119.50	96.81	200 HSCs	1 HSC
Pkp3	chr7	E13.1_B	E6.43_B	0.44	0.84	2.29E-35	0.40	75.25	71.84	200 HSCs	1 HSC
Pkp3	chr7	E15.2_B	E13.29_B	0.56	0.94	1.02E-39	0.39	119.50	47.17	200 HSCs	1 HSC
Pkp3	chr7	E15.2_B	E15.10_B	0.56	0.18	1.96E-46	0.38	119.50	79.42	200 HSCs	1 HSC
Pkp3	chr7	E6.2_B	E6.43_B	0.47	0.84	4.44E-24	0.37	55.39	71.84	50 HSCs	1 HSC
Pkp3	chr7	E6.2_B	E6.42_B	0.47	0.12	2.65E-33	0.34	55.39	96.81	50 HSCs	1 HSC
Pkp3	chr7	E13.1_B	E6.42_B	0.44	0.12	1.08E-40	0.32	75.25	96.81	200 HSCs	1 HSC
Pkp3	chr7	E15.10_B	E6.2_B	0.18	0.47	5.09E-21	0.29	79.42	55.39	1 HSC	50 HSCs
Pkp3	chr7	E15.2_B	E6.43_B	0.56	0.84	1.67E-19	0.28	119.50	71.84	200 HSCs	1 HSC
Pkp3	chr7	E13.2_B	E6.42_B	0.39	0.12	3.05E-34	0.27	79.17	96.81	200 HSCs	1 HSC
Pkp3	chr7	E13.1_B	E15.10_B	0.44	0.18	7.47E-26	0.26	75.25	79.42	200 HSCs	1 HSC
Pkp3	chr7	E13.2_B	E15.10_B	0.39	0.18	2.00E-20	0.21	79.17	79.42	200 HSCs	1 HSC
Pkp3	chr7	E6.1_B	E6.42_B	0.32	0.12	4.60E-21	0.19	110.71	96.81	50 HSCs	1 HSC
Pkp3	chr7	E15.10_B	E6.1_B	0.18	0.32	1.84E-10	0.14	79.42	110.71	1 HSC	50 HSCs
Pkp3	chr7	E15.2_B	E6.1_B	0.56	0.32	5.36E-21	0.24	119.50	110.71	200 HSCs	50 HSCs
Pkp3	chr7	E13.2_B	E15.2_B	0.39	0.56	7.22E-10	0.16	79.17	119.50	200 HSCs	200 HSCs
Pkp3	chr7	E6.1_B	E6.2_B	0.32	0.47	2.62E-06	0.15	110.71	55.39	50 HSCs	50 HSCs
Pkp3	chr7	E13.1_B	E6.1_B	0.44	0.32	4.24E-07	0.12	75.25	110.71	200 HSCs	50 HSCs

Trim5	chr7	E13.24_B	E6.43_B	0.63	0.29	6.30E-13	0.34	64.65	38.39	1 HSC	1 HSC
Trim5	chr7	E15.10_B	E6.43_B	0.64	0.29	4.36E-14	0.34	39.52	38.39	1 HSC	1 HSC
Trim5	chr7	E13.29_B	E6.43_B	0.62	0.29	7.95E-16	0.33	88.81	38.39	1 HSC	1 HSC
Trim5	chr7	E13.24_B	E6.42_B	0.63	0.36	3.39E-11	0.27	64.65	42.07	1 HSC	1 HSC
Trim5	chr7	E15.10_B	E6.42_B	0.64	0.36	1.56E-12	0.27	39.52	42.07	1 HSC	1 HSC
Trim5	chr7	E13.29_B	E6.42_B	0.62	0.36	7.46E-15	0.26	88.81	42.07	1 HSC	1 HSC
Trim5	chr7	E6.2_B	E6.43_B	0.63	0.29	4.23E-14	0.33	78.92	38.39	50 HSCs	1 HSC
Trim5	chr7	E13.1_B	E6.43_B	0.59	0.29	8.62E-13	0.30	70.32	38.39	200 HSCs	1 HSC
Trim5	chr7	E6.1_B	E6.43_B	0.56	0.29	6.88E-11	0.27	88.97	38.39	50 HSCs	1 HSC
Trim5	chr7	E6.2_B	E6.42_B	0.63	0.36	1.30E-12	0.26	78.92	42.07	50 HSCs	1 HSC
Trim5	chr7	E13.1_B	E6.42_B	0.59	0.36	2.40E-11	0.23	70.32	42.07	200 HSCs	1 HSC
Trim5	chr7	E13.2_B	E6.43_B	0.51	0.29	5.15E-08	0.22	73.83	38.39	200 HSCs	1 HSC
Trim5	chr7	E15.2_B	E13.24_B	0.43	0.63	3.62E-07	0.20	71.47	64.65	200 HSCs	1 HSC
Trim5	chr7	E15.2_B	E15.10_B	0.43	0.64	4.56E-08	0.20	71.47	39.52	200 HSCs	1 HSC
Trim5	chr7	E6.1_B	E6.42_B	0.56	0.36	2.97E-09	0.20	88.97	42.07	50 HSCs	1 HSC
Trim5	chr7	E15.2_B	E13.29_B	0.43	0.62	1.85E-09	0.19	71.47	88.81	200 HSCs	1 HSC
Trim5	chr7	E13.2_B	E6.42_B	0.51	0.36	3.93E-06	0.15	73.83	42.07	200 HSCs	1 HSC
Trim5	chr7	E15.2_B	E6.2_B	0.43	0.63	5.15E-08	0.19	71.47	78.92	200 HSCs	50 HSCs
Trim5	chr7	E13.1_B	E15.2_B	0.59	0.43	1.13E-06	0.16	70.32	71.47	200 HSCs	200 HSCs
Fam32a	chr8	E13.29_B	E6.43_B	0.50	0.02	4.72E-119	0.48	203.22	102.07	1 HSC	1 HSC
Fam32a	chr8	E15.10_B	E6.43_B	0.47	0.02	9.48E-113	0.46	255.71	102.07	1 HSC	1 HSC
Fam32a	chr8	E6.42_B	E6.43_B	0.45	0.02	4.55E-103	0.44	239.69	102.07	1 HSC	1 HSC
Fam32a	chr8	E13.24_B	E6.43_B	0.42	0.02	6.73E-82	0.40	238.14	102.07	1 HSC	1 HSC
Fam32a	chr8	E13.24_B	E13.29_B	0.42	0.50	1.95E-06	0.08	238.14	203.22	1 HSC	1 HSC
Fam32a	chr8	E15.2_B	E6.43_B	0.44	0.02	8.56E-98	0.43	317.59	102.07	200 HSCs	1 HSC
Fam32a	chr8	E13.2_B	E6.43_B	0.44	0.02	8.11E-99	0.42	197.30	102.07	200 HSCs	1 HSC
Fam32a	chr8	E13.1_B	E6.43_B	0.41	0.02	8.05E-86	0.39	189.71	102.07	200 HSCs	1 HSC
Fam32a	chr8	E6.1_B	E6.43_B	0.39	0.02	6.52E-82	0.37	261.42	102.07	50 HSCs	1 HSC

Fam32a	chr8	E6.2_B	E6.43_B	0.36	0.02	6.03E-68	0.35	163.06	102.07	50 HSCs	1 HSC
Fam32a	chr8	E13.29_B	E6.2_B	0.50	0.36	2.93E-15	0.13	203.22	163.06	1 HSC	50 HSCs
Fam32a	chr8	E13.29_B	E6.1_B	0.50	0.39	6.50E-18	0.11	203.22	261.42	1 HSC	50 HSCs
Fam32a	chr8	E15.10_B	E6.2_B	0.47	0.36	1.82E-11	0.11	255.71	163.06	1 HSC	50 HSCs
Fam32a	chr8	E13.1_B	E13.29_B	0.41	0.50	3.18E-11	0.09	189.71	203.22	200 HSCs	1 HSC
Fam32a	chr8	E15.10_B	E6.1_B	0.47	0.39	6.07E-13	0.09	255.71	261.42	1 HSC	50 HSCs
Fam32a	chr8	E6.2_B	E6.42_B	0.36	0.45	9.54E-08	0.09	163.06	239.69	50 HSCs	1 HSC
Fam32a	chr8	E13.1_B	E15.10_B	0.41	0.47	2.22E-07	0.07	189.71	255.71	200 HSCs	1 HSC
Fam32a	chr8	E13.2_B	E13.29_B	0.44	0.50	3.15E-06	0.06	197.30	203.22	200 HSCs	1 HSC
Fam32a	chr8	E6.1_B	E6.42_B	0.39	0.45	1.30E-07	0.06	261.42	239.69	50 HSCs	1 HSC
Fam32a	chr8	E15.2_B	E6.2_B	0.44	0.36	2.79E-06	0.08	317.59	163.06	200 HSCs	50 HSCs
Slc38a7	chr8	E13.24_B	E13.29_B	0.60	0.13	2.25E-18	0.47	21.53	11.72	1 HSC	1 HSC
Slc38a7	chr8	E13.29_B	E6.43_B	0.13	0.53	6.39E-14	0.40	11.72	20.15	1 HSC	1 HSC
Slc38a7	chr8	E13.29_B	E6.42_B	0.13	0.51	7.50E-17	0.38	11.72	30.82	1 HSC	1 HSC
Slc38a7	chr8	E13.29_B	E15.10_B	0.13	0.44	2.39E-11	0.31	11.72	20.46	1 HSC	1 HSC
Slc38a7	chr8	E13.29_B	E6.1_B	0.13	0.59	1.33E-23	0.46	11.72	32.01	1 HSC	50 HSCs
Slc38a7	chr8	E15.2_B	E13.29_B	0.57	0.13	3.51E-19	0.44	33.63	11.72	200 HSCs	1 HSC
Slc38a7	chr8	E13.2_B	E13.29_B	0.56	0.13	3.06E-20	0.43	22.21	11.72	200 HSCs	1 HSC
Slc38a7	chr8	E13.1_B	E13.29_B	0.49	0.13	1.60E-14	0.36	25.01	11.72	200 HSCs	1 HSC
Slc38a7	chr8	E13.29_B	E6.2_B	0.13	0.45	9.03E-10	0.32	11.72	16.73	1 HSC	50 HSCs
Apba3	chr10	E13.29_B	E6.43_B	0.52	0.02	9.71E-20	0.50	31.56	18.60	1 HSC	1 HSC
Apba3	chr10	E13.29_B	E15.10_B	0.52	0.05	2.04E-29	0.47	31.56	20.35	1 HSC	1 HSC
Apba3	chr10	E6.42_B	E6.43_B	0.48	0.02	1.26E-18	0.46	59.59	18.60	1 HSC	1 HSC
Apba3	chr10	E13.24_B	E6.43_B	0.46	0.02	9.78E-15	0.44	31.16	18.60	1 HSC	1 HSC
Apba3	chr10	E15.10_B	E6.42_B	0.05	0.48	7.11E-29	0.42	20.35	59.59	1 HSC	1 HSC
Apba3	chr10	E13.24_B	E15.10_B	0.46	0.05	1.55E-20	0.40	31.16	20.35	1 HSC	1 HSC
Apba3	chr10	E15.2_B	E6.43_B	0.60	0.02	1.84E-26	0.58	67.69	18.60	200 HSCs	1 HSC
Apba3	chr10	E13.2_B	E6.43_B	0.57	0.02	4.05E-24	0.55	34.72	18.60	200 HSCs	1 HSC

Apba3	chr10	E15.2_B	E15.10_B	0.60	0.05	2.34E-40	0.55	67.69	20.35	200 HSCs	1 HSC
Apba3	chr10	E13.1_B	E6.43_B	0.54	0.02	4.77E-21	0.52	33.81	18.60	200 HSCs	1 HSC
Apba3	chr10	E6.2_B	E6.43_B	0.54	0.02	2.04E-18	0.52	25.21	18.60	50 HSCs	1 HSC
Apba3	chr10	E13.2_B	E15.10_B	0.57	0.05	4.62E-37	0.51	34.72	20.35	200 HSCs	1 HSC
Apba3	chr10	E6.1_B	E6.43_B	0.53	0.02	3.08E-22	0.51	53.93	18.60	50 HSCs	1 HSC
Apba3	chr10	E13.1_B	E15.10_B	0.54	0.05	1.21E-31	0.48	33.81	20.35	200 HSCs	1 HSC
Apba3	chr10	E15.10_B	E6.1_B	0.05	0.53	9.84E-35	0.48	20.35	53.93	1 HSC	50 HSCs
Apba3	chr10	E15.10_B	E6.2_B	0.05	0.54	2.67E-26	0.48	20.35	25.21	1 HSC	50 HSCs
Zfp873	chr10	E13.1_B	E6.43_B	0.54	0.06	1.60E-07	0.48	26.78	17.10	200 HSCs	1 HSC
Zfp873	chr10	E6.1_B	E6.43_B	0.52	0.06	1.39E-07	0.46	46.67	17.10	50 HSCs	1 HSC
Zfp873	chr10	E15.2_B	E6.43_B	0.52	0.06	1.52E-06	0.45	33.69	17.10	200 HSCs	1 HSC
Zfp873	chr10	E6.2_B	E6.43_B	0.48	0.06	4.06E-06	0.42	45.50	17.10	50 HSCs	1 HSC
Flnb	chr14	E15.10_B	E6.43_B	0.47	0.11	3.74E-08	0.36	32.79	15.31	1 HSC	1 HSC
Flnb	chr14	E13.24_B	E6.43_B	0.45	0.11	1.39E-06	0.34	26.12	15.31	1 HSC	1 HSC
Flnb	chr14	E6.42_B	E6.43_B	0.40	0.11	4.40E-06	0.29	34.86	15.31	1 HSC	1 HSC
Flnb	chr14	E13.29_B	E15.10_B	0.20	0.47	2.12E-10	0.27	25.82	32.79	1 HSC	1 HSC
Flnb	chr14	E13.24_B	E13.29_B	0.45	0.20	9.07E-07	0.24	26.12	25.82	1 HSC	1 HSC
Flnb	chr14	E13.29_B	E6.42_B	0.20	0.40	1.40E-06	0.20	25.82	34.86	1 HSC	1 HSC
Flnb	chr14	E15.2_B	E6.43_B	0.62	0.11	3.08E-12	0.51	23.97	15.31	200 HSCs	1 HSC
Flnb	chr14	E15.2_B	E13.29_B	0.62	0.20	7.49E-16	0.42	23.97	25.82	200 HSCs	1 HSC
Flnb	chr14	E13.1_B	E6.43_B	0.52	0.11	2.38E-10	0.41	39.50	15.31	200 HSCs	1 HSC
Flnb	chr14	E13.2_B	E6.43_B	0.45	0.11	6.92E-08	0.34	44.62	15.31	200 HSCs	1 HSC
Flnb	chr14	E13.1_B	E13.29_B	0.52	0.20	6.39E-15	0.32	39.50	25.82	200 HSCs	1 HSC
Flnb	chr14	E6.1_B	E6.43_B	0.41	0.11	2.80E-06	0.30	27.33	15.31	50 HSCs	1 HSC
Flnb	chr14	E13.2_B	E13.29_B	0.45	0.20	1.41E-10	0.25	44.62	25.82	200 HSCs	1 HSC
Flnb	chr14	E13.29_B	E6.1_B	0.20	0.41	7.56E-07	0.21	25.82	27.33	1 HSC	50 HSCs
Pacsin1	chr17	E13.24_B	E6.42_B	0.29	0.70	3.27E-10	0.40	24.21	29.09	1 HSC	1 HSC
Pacsin1	chr17	E6.42_B	E6.43_B	0.70	0.31	1.69E-06	0.38	29.09	10.74	1 HSC	1 HSC

Pacsin1	chr17	E13.29_B	E6.42_B	0.32	0.70	5.11E-11	0.37	17.04	29.09	1 HSC	1 HSC
Pacsin1	chr17	E13.24_B	E15.10_B	0.29	0.63	6.09E-07	0.33	24.21	25.47	1 HSC	1 HSC
Pacsin1	chr17	E13.29_B	E15.10_B	0.32	0.63	3.14E-07	0.30	17.04	25.47	1 HSC	1 HSC
Pacsin1	chr17	E13.1_B	E13.24_B	0.62	0.29	6.78E-07	0.33	26.21	24.21	200 HSCs	1 HSC
Pacsin1	chr17	E13.1_B	E13.29_B	0.62	0.32	3.53E-07	0.30	26.21	17.04	200 HSCs	1 HSC
Pacsin1	chr17	E15.2_B	E6.42_B	0.42	0.70	1.89E-06	0.28	26.33	29.09	200 HSCs	1 HSC

Supplementary Table 5. Probability of finding AI for any given gene in the monoclonal animals due to somatic indels and single nucleotide variants (null hypothesis). The parameter values based on the literature that are overestimations (as explained in the comments) are shown in bold. We privileged studies of murine cells (references in bold) and used studies on human cells as the second-best option (references in italics). Most parameter values based on assumptions (in italics) are also overestimations. This estimation suggests that not all AI patterns of the 14 genes we identify can be explained by somatic mutations. The most notable example is *Pkp3*, for which we show the probability (which led us to reject the null hypothesis of a genetic mutation explanation for the AI pattern).

	**Value for estimation**	**Comment**	**Ref.**
SNV per HSC (n_s)	**195**	Overestimation. We used the HSC with the highest number of mutations (195). The average number is around 105. The animals used for WGS were 8-month old, and ours were <5 months (mutations accumulate over time).	**(Druce, 2021)**
Indels per HSC (n_i)	**42**	Overestimation. The HSC with the highest number of mutations (42) was used. The average number is around 26.	
Total number of genes expressed by a cell (n_g)	**24,000**	Overestimation. The average number of expressed genes in a given cell is 10,700, but all genes were assumed to be expressed.	***(Ramsköld, Wang, Burge, & Sandber, 2009)***
Intergenic mutations ($P_{intergenic}$)		These mutations were assumed to have no impact on AI.	
**Mutations in cis**			
Impact of indels in exons (P_{iie})	*1.000*	Overestimation. All indels are assumed to lead to non-sense mediated mRNA decay, which is unlikely.	
Impact of indels in introns (P_{iii})	*0.200*	Overestimation. 20% of indels in introns are assumed to lead to AI, which is unlikely.	
Impact of indels in regulatory regions (P_{iir})	*0.200*	Overestimation. 20% of indels in regulatory regions are assumed to lead to AI, which is unlikely.	
Frequency of indels in exons (P_{fie})	0.015	The values were assumed to be identical to the frequencies of SNVs.	
Frequency of indels in introns (P_{fii})	0.261		
Frequency of indels in regulatory regions (P_{fir})	0.020		
**Probability of impactful cis indel in a given gene (P_{icis})** $1-(1-P_{iie}*P_{fie}/n_g)^{ni}*(1-P_{iii}*P_{fii}/n_g)^{ni}*(1-P_{iir}*P_{firi}/n_g)^{ni}$	$1.25*10^{-4}$		
Impact on transcription of SNVs in exons (P_{ise})	0.061	Empirical frequency of premature stop codons.	**(Druce, 2021)**
Impact on transcription SNVs in introns (P_{isi})	0.200	20% of SNVs in introns are assumed to lead to AI, which is unlikely.	

Impact on transcription SNVs regulatory regions (P_{isr})	0.200	20% of SNVs in regulatory regions are assumed to lead to AI, which is unlikely.	
Frequency of SNVS in exons (P_{fse})	0.015	Empirical estimations.	**(Druce, 2021)**
Frequency of SNVs in introns (P_{fsi})	0.261		
Frequency of SNVs in regulatory regions (P_{fsr})	0.020		
**Probability of impactful cis SNV in a given gene (P_{scis})** $1\text{-}(1\text{-} P_{ise} * P_{fse}/ n_g)^{ns} *(1\text{-} P_{isi} * P_{fsi}/ n_g)^{ns} *(1\text{-} P_{isr} * P_{fsri}/ n_g)^{ns}$	$4.64*10^{-4}$		
**Mutations in trans**			
**Transcription factors**			
Number of transcription factors	1,500		**(Zhou, Liu, Xia, Gong, Feng, Liu et al., 2017)**
Probability of affecting transcription in trans. Only mutations changing the protein (exonic non-synonymous) were assumed to impact the transcription of a gene in trans (if they affect a region of the protein that interacts with the alleles) ($P_{TFtrans}$)	*0.2*	Overestimation. Gene-specific transcription factors are relatively small (~50 kDa/1.35 kb). Promoters typically recognize binding sites of 10 bp (5-31 bp). The homeobox sequence encodes the homeodomain, a globular domain of about 60 amino acids that normally functions as a DNA-binding domain. That's roughly 13% of the TF.	(***Stewart, Hannenhalli, & Plotkin, 2012***; **Bürglin, & Affolter, 2016**)
Probability of interfering with AI (if the mutation and SNP are close) ($P_{TFtransAI}$)	*0.5*	Overestimation.	
Number of transcription factors regulating a gene (n_{tfg})	**58**	Overestimation. 90% of genes have between 0 and 26 transcription factor binding sites. The maximum number is 58.	(*Hurst, Sachenkova, Daub, Forrest, & Huminiecki, 2014*)
**Probability of impactful mutation from a transcription factor gene ($P_{Tftrans}$)** $1\text{-}(1\text{-} P_{ise} * P_{fse} *P_{TFtrans} * P_{TFtransAI} * n_{tfg} / n_g)^{ns} *(1\text{-} P_{fie} *P_{TFtrans} * P_{TFtransAI} * n_{tfg} / n_g)^{ni}$	$1.95*10^{-4}$		
**RNA binding proteins**			
Number of RNA binding proteins	1,870	The database contains 292 mouse RNA-seq datasets for a comprehensive list of 187 RNA-binding proteins (RBPs). These RBPs account for only 10% of all known RBPs annotated in Gene Ontology, indicating that most are still unexplored using high-throughput sequencing.	**(Li, Deng, Vieira, Thomas, Costa, Tseng et al., 2018)**
Assume only mutations changing the protein (exonic non-synonymous) can impact the transcription of a gene in cis if they affect a region of the protein that interacts with the SNP distinguishing the two alleles. ($P_{RNAptrans}$)	0.2		

Probability of interfering with AI (if the mutation and SNP are close) ($P_{RNAptransAI}$)	0.1		
Number of RNA binding proteins regulating a gene (n_{rnap})	197	On average, a human mRNA transcript has 197 RBP binding sites (median 85 binding sites).	(*Liu, Pan, Kong, Luo, & Zhang, 2020*)
**Probability of impactful mutation from an RNA protein gene ($P_{RNAptrans}$)** $1-(1- P_{ise} * P_{fse} * P_{RNAptrans} * P_{RNAptransAI} * n_{rnap} / n_g)^{ns} *(1- P_{fie} * P_{RNAptrans} * P_{RNAptransAI} * n_{rnap} / n_g)^{ni}$	1.38 $*10^{-4}$		
**REGULATORY RNAs (miRNA, snRNA, miscellaneous small noncoding, and lncRNA)**			
The number of RNA regulatory RNAs (n_{rnareg})	3,918		(**Isakova, Fehlmann, Keller, & Quake, 2020; Zhou, Wan, Jiang, Liu, Qiang, & Sun, 2020**)
Assume only mutations changing the RNA (exonic) can impact the transcription of a gene in cis if they affect a region of the RNA that interacts with the two alleles ($P_{RNAregtrans}$)	*0.15*	Informed assumption.	
Probability of interfering with AI (if the mutation and SNP are close) ($P_{RNAregtransAI}$)	*0.5*	Overestimation.	
The average number of regulatory RNAs regulating a gene (n_{RNAreg})	195		(*Gennarino, D'Angelo, Dharmalingam, Fernandez, Russolillo, Sanges et al., 2012*)
**Probability of impactful mutation from a regulatory RNA gene ($P_{RNAregtrans}$)** $1-(1- P_{ise} * P_{fse} * P_{RNAregtrans} * P_{RNAregtransAI} * n_{RNAreg} / n_g)^{ns} *(1- P_{fie} * P_{RNAregtrans} * P_{RNAregtransAI} * n_{RNAreg} / n_g)^{ni}$	4.92 $*10^{-4}$		
**PROBABILITY OF AT LEAST ONE MUTATION IMPACTING AI IN A GIVEN GENE (P_{AI})** $1-(1- P_{icis})*(1- P_{scis})*(1- P_{TFtrans})*(1- P_{RNAptrans})*(1- P_{RNAregtrans})$	$1.41*10^{-3}$		
Considering **P_{AI}** and the binomial distribution, the probability of finding 4 animals in 5 with an AI deviating from 0.5 (in any direction) for a given gene (as observed for *Pkp3*) is:<br><br>0.001 alpha value with Bonferroni correction for the total number of analyzed genes (7,088) is $1.4*10^{-6}$	$1.97*10^{-11}$		

Supplementary Table 6. *Pkp3* AI data from the 28 libraries. We emphasize the concordance of the AI data between replicas of the same samples (3rd and 4th columns; r^2=0.987, P= $1.577*10^{-14}$) and between B and T cells (AI average, in red; r^2=0.976, P= 0.0123) from the same animal (E13, in bold). The dispersion (standard deviation) of the monoclonal and polyclonal AI values for B cells is distinct (asterisks, F test, P-value = 0.0155).

		AI			Group	
**Clonality**	**Sample**	**Replicate 1**	**Replicate 2**	**Average**	**Mean**	**STD**
**B cells**						
nonclonal	Control	0.426	0.472	0.449	-	-
polyclonal	**E13.1**	**0.451**	**0.431**	**0.441**	0.318	0.087*
	**E13.2**	**0.397**	**0.392**	**0.394**		
	E6.1	0.313	0.323	0.318		
	E6.2	0.467	0.432	0.450		
	E15.2	0.560	0.554	0.557		
monoclonal	**E13.24**	**0.417**	**0.435**	**0.426**	0.380	0.373*
	**E13.29**	**0.946**	**0.939**	**0.943**		
	E6.42	0.148	0.101	0.124		
	E6.43	0.842	0.830	0.836		
	E15.10	0.176	0.185	0.181		
**T cells**						
nonclonal	Control	0.432	0.478	0.455	-	-
polyclonal	**E13.1**	**0.531**	**0.535**	**0.533**	**0.464**	**0.097**
	**E13.2**	**0.370**	**0.421**	**0.395**		
monoclonal	**E13.24**	**0.433**	**0.411**	**0.422**	**0.708**	**0.404**
	**E13.29**	**0.987**	**1.000**	**0.994**		

Supplementary Figures

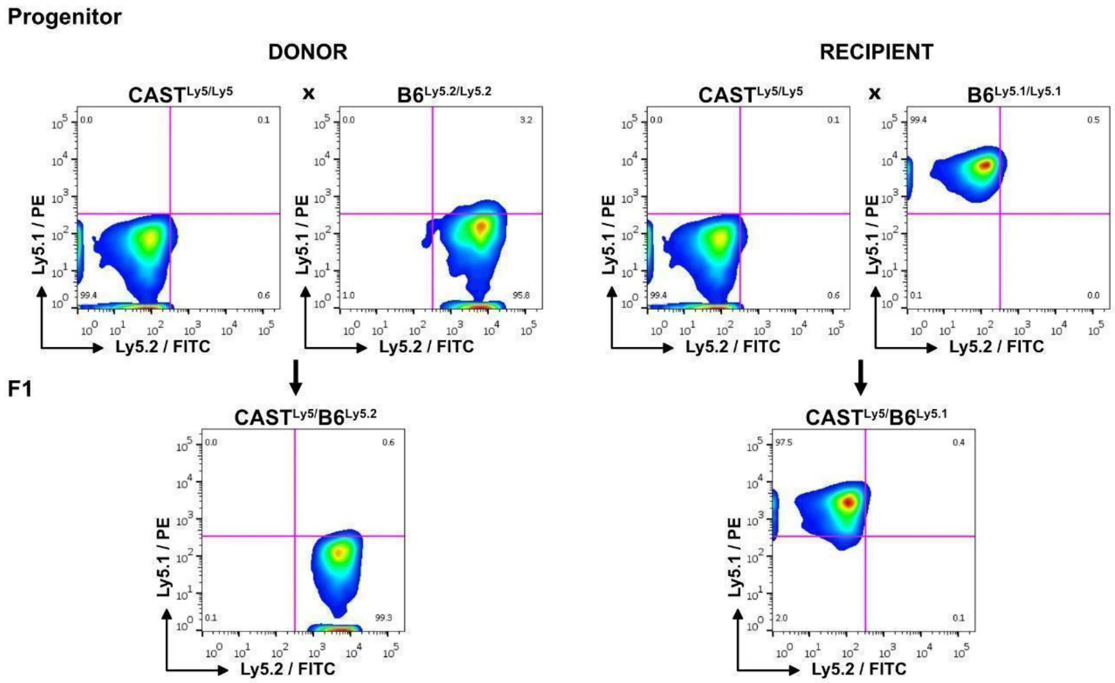

Supplementary Figure 1. Ly5.1 and Ly5.2 pan-leukocytic markers were used to distinguish recipient and donor cells in reconstituted animals, respectively. Ly5.1 and Ly5.2 do not label the CAST progenitor line, and when CAST is crossed with B6$^{Ly5.1/Ly5.1}$ or B6$^{Ly5.2/Ly5.2,}$ to produce the recipient and donor F1 animals, respectively, the recipient and donor cells are distinguishable by these two markers. Blood samples of progenitor and descendants (F1) were lysed for red cells, stained with FITC-conjugated anti-Ly5.2 and PE-conjugated anti-Ly5.1, and analyzed using FACSCanto.

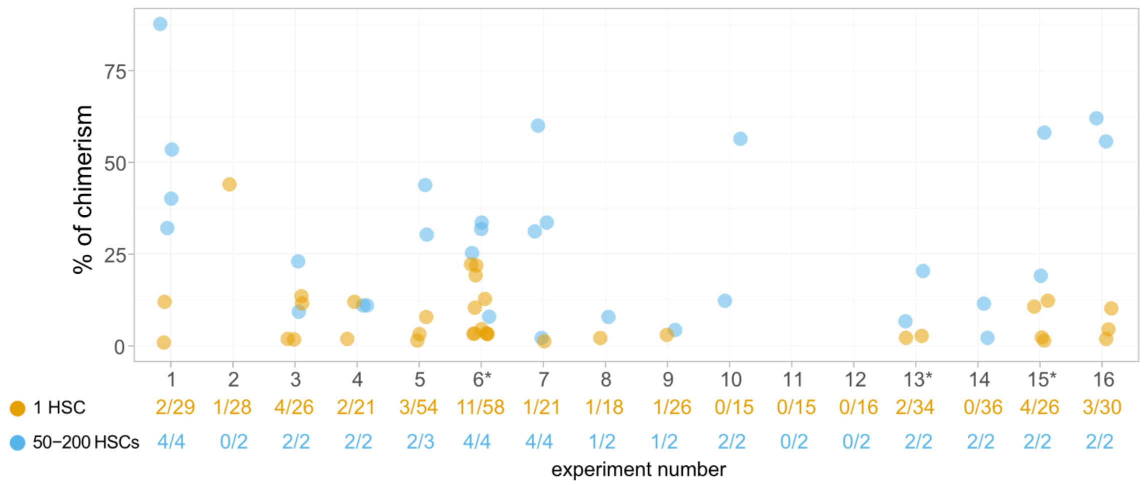

Supplementary Figure 2. Percentages of chimerism identified in the blood of reconstituted animals for 16 experiments at 12 weeks post-injection (orange dots, monoclonal animals; blue dots, polyclonal animals). An animal was considered reconstituted if the chimerism percentage was above 1%. The sequenced samples in this study belong to experiments 6, 13, and 15 (marked with asterisk (*)).

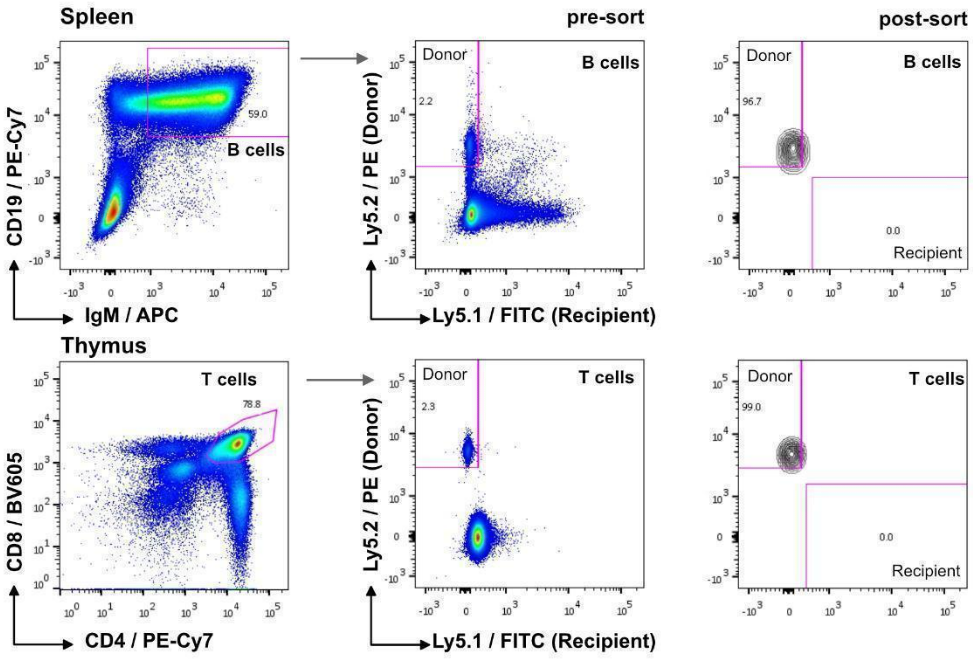

Supplementary Figure 3. Representative plots of pre-sorted and post-sorted B/T-cell populations of an animal reconstituted with a single HSC. Cells from the spleen and thymus of recipient animals were isolated, stained for B-cell markers with PE anti-Ly5.2, FITC anti-Ly5.1, and PE-Cy7 anti-CD19 and APC anti-IgM (splenocytes), or T-cell markers with PE-Cy7 anti-CD4 and BV605 anti-CD8 (thymocytes), and sorted on a FACSAria. The cells were gated for PI^- to exclude dead cells and on $Ly5.2^+/Ly5.1^-$ to obtain pure donor cells, and then for $CD19^+/IgM^+$ to select B-cells or for $CD4^+/CD8^+$ to select for T-cells. The purity of sorted cells was assessed by analyzing 150–250 of the sorted cells.

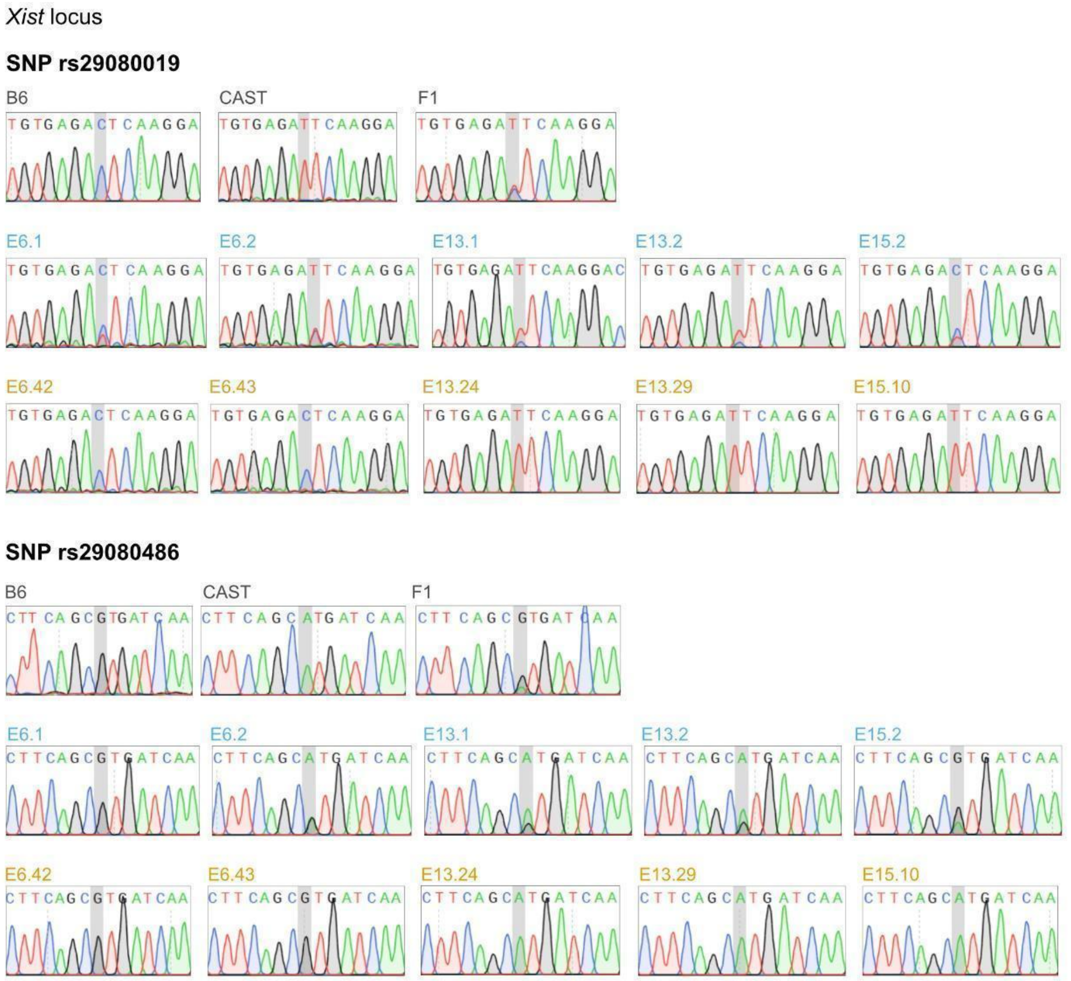

Supplementary Figure 4. Monoclonality screening was used to confirm if the recipient system was reconstituted with a single HSC. The cDNA Sanger sequencing chromatograms cover a region with two SNPs in the *Xist locus* that allow us to assign the *Xist* transcript to the CAST or B6 X chromosome. Due to XCI, when a single cell is used for the reconstitution, a single peak is expected in the position of the SNP; when multiple cells were used for reconstitutions, two peaks should be observed in each of the SNP positions. Samples E6.1, E6.2, E13.1, E13.2 and E13.5 are cells expanded after the injection of multiple HSCs (polyclonal samples), samples E6.42, E6.43, E13.24, E13.29 and E15.10 are cells expanded after the injection of a single HSC (monoclonal samples).

A.

SNPs (Ly5/Ptprc)		Donor (CAST$^{Ly5/Ly5}$ x B6$^{Ly5.2/Ly5.2}$)		Recipient (CAST$^{Ly5/Ly5}$ x B6$^{Ly5.1/Ly5.1}$)	
**Position_id**		**CAST^{Ly5}**	**B6$^{Ly5.2}$**	**CAST^{Ly5}**	**B6$^{Ly5.1}$**
1	**1_138107837**_rs33604282	**T**	**T**	**T**	**C**
	-	-	-	-	-
2	**1_138107824**_rs260353747	**G**	**G**	**G**	**T**
3	**1_138107823**_rs33604283	**T**	**T**	**T**	**C**

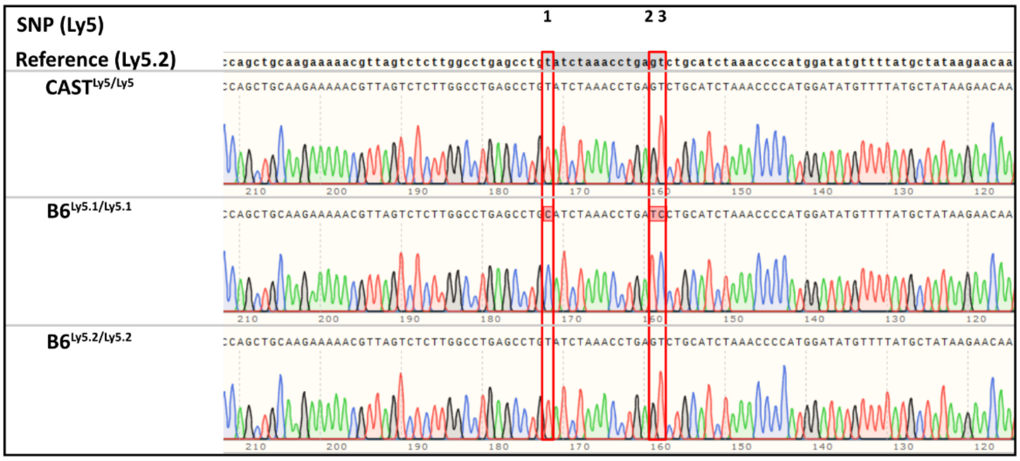

B.

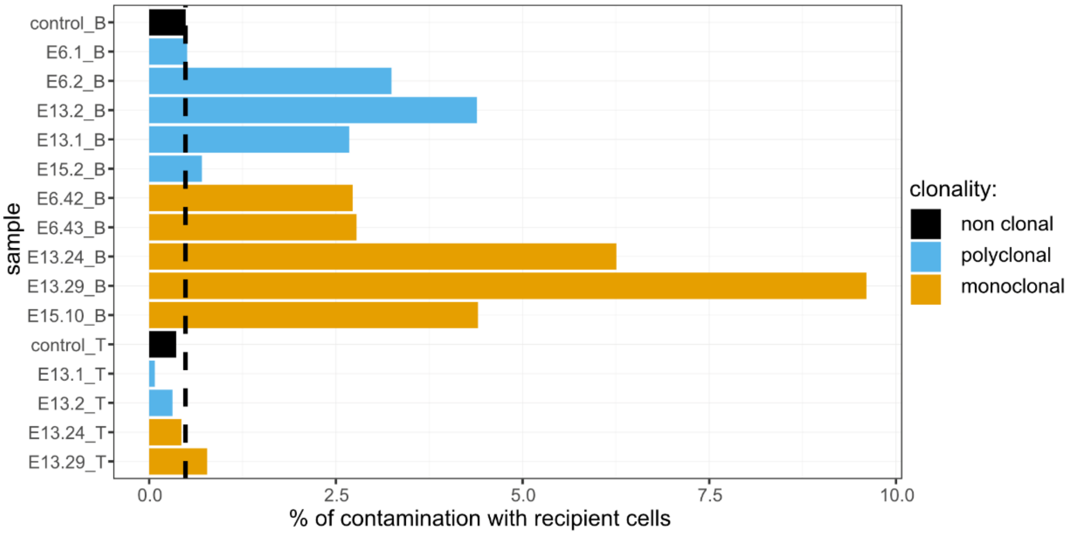

Supplementary Figure 5. Estimation of donor population contamination with recipient cells. **(A)** Identification and cDNA Sanger sequencing focusing on three different SNPs for the *Ly5* gene that distinguish two pan-leukocytic markers, Ly5.1 and Ly5.2, present in the recipient and donor animals, respectively. CAST and B6 *Ly5.2 loci* are indistinguishable, but B6 *Ly5.1* has different variation, allowing the estimation of the level of recipient cell contamination in the sorted donor cell populations. **(B)** Percentages of recipient cells (Ly5.1^{+} cells) estimated in the sorted donor cell populations based on next-generation sequencing results. Nucleotide bases for *Ly5.1* and *Ly5.2* were counted for each SNP position. The bar corresponds to the average of two replicates. The dashed line (0.5%) represents the percentage of artifactual SNPs due to errors introduced by sequencing, which was estimated by sequencing of an unmanipulated donor mouse.

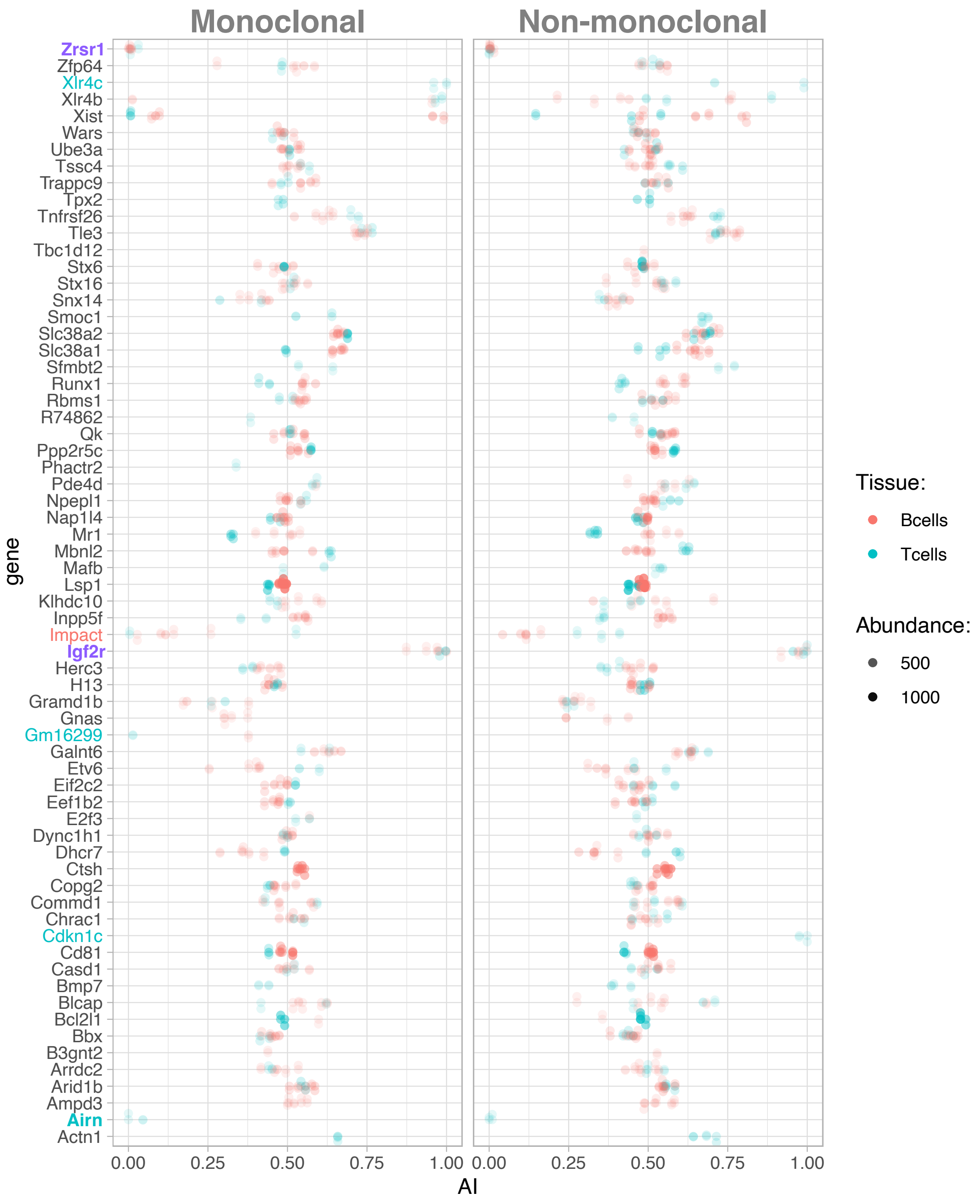

Supplementary Figure 6. Imprinted genes expressed in B cells or T cells in this study. The list of imprinted genes was retrieved from https://www.geneimprint.com and from Tucci et al., 2019. A total of 66 imprinted genes were expressed (55 in B cells and 62 in T cells), out of 167 imprinted genes discoverable in the annotation file used in this study (ftp://ftp.ensembl.org/pub/release-68/gtf/Mus_musculus.GRCm38.68.gtf). Genes highlighted in purple are randomly biased in B cells and T cells. Genes highlighted in light blue or light red could have tissue-specific interest, although not necessarily reaching our criteria for "randomly biased". Genes in bold were classified as randomly biased. Genes with low expression levels (<10 TMM-normalized counts) and genes suspected of LOH (detected by WES) were not included. This figure is related to **Supplementary Table 2**.

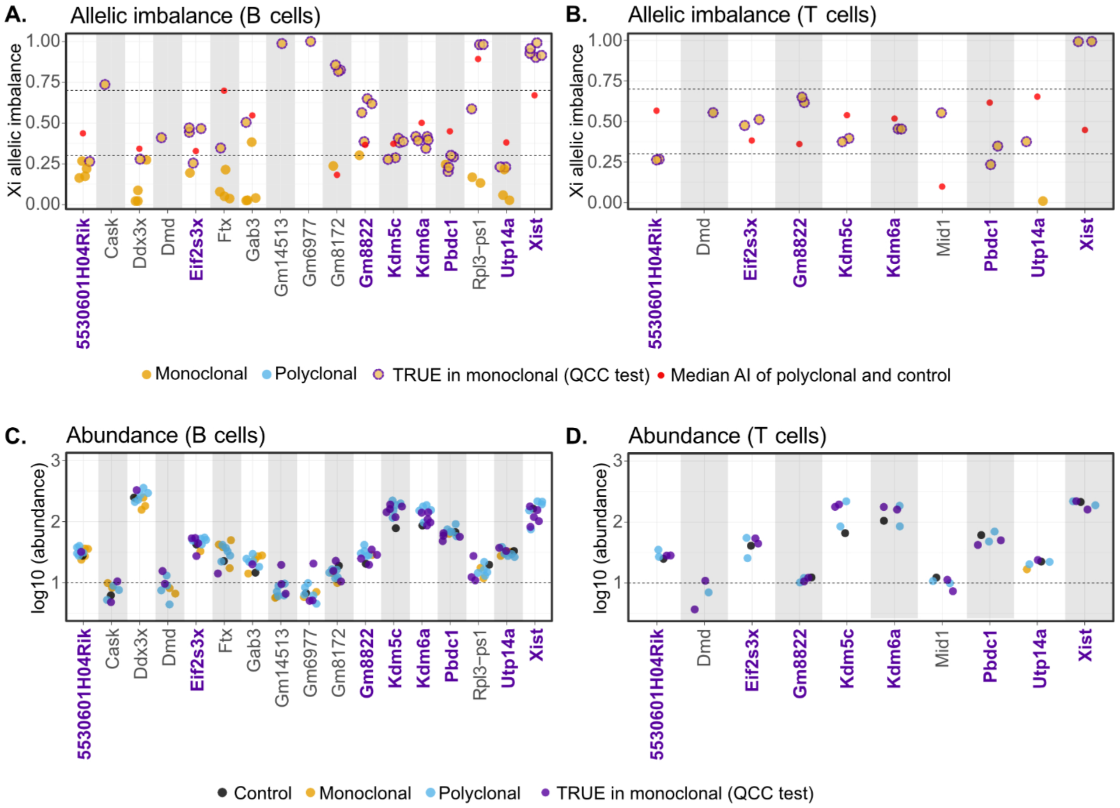

Supplementary Figure 7. Identification of XCI escapees. **(A), (B)** AI of X-linked genes for B and T cells. As a convention, an AI=1 means that the gene is 100% expressed from the allele of the inactive chromosome X (Xi); *Xi allelic imbalance=1* means that the gene is 100% expressed from the inactive X-linked allele; *Xi allelic imbalance=0* means that only the active X-linked allele was detected. Dots represent genes with expression higher than 10 TMM-normalized counts and only genes that were statistically different from the threshold at least once are shown. Yellow dots represent monoclonal samples; dotted violet stroke surrounding yellow dots denote statistical significance for that sample. Red dots represent the median of the AI observed for polyclonal and control samples (which are otherwise excluded from this top panel). Statistical significance was calculated by comparison of the AI with the sample-corrected threshold using binomial test and QCC correction. The threshold was calculated per sample, as 0.1 (which is the value usually found in the literature) + the value of the median of AI of all X-linked genes in the sample. **(C), (D)** Abundance (TMM-normalized counts) of the same genes and same samples represented in (A), (B). In addition, individual polyclonal and control samples are shown, as well as samples with abundance <10. Violet dots represent the monoclonal samples in which the AI significantly deviates from the sample-corrected threshold. Yellow dots represent the other monoclonal samples, blue dots, the polyclonal samples, and black dots are the control samples. Genes in violet (*x-axis*) were identified as escapees using three criteria: 1) only samples with abundance higher than 10 were considered; 2) the median of AI in the control samples (polyclonal and control samples) was balanced (0.5±0.2); and 3) the AI of the gene is statistically different from the threshold in at least two samples, irrespective of the tissue.

B cells

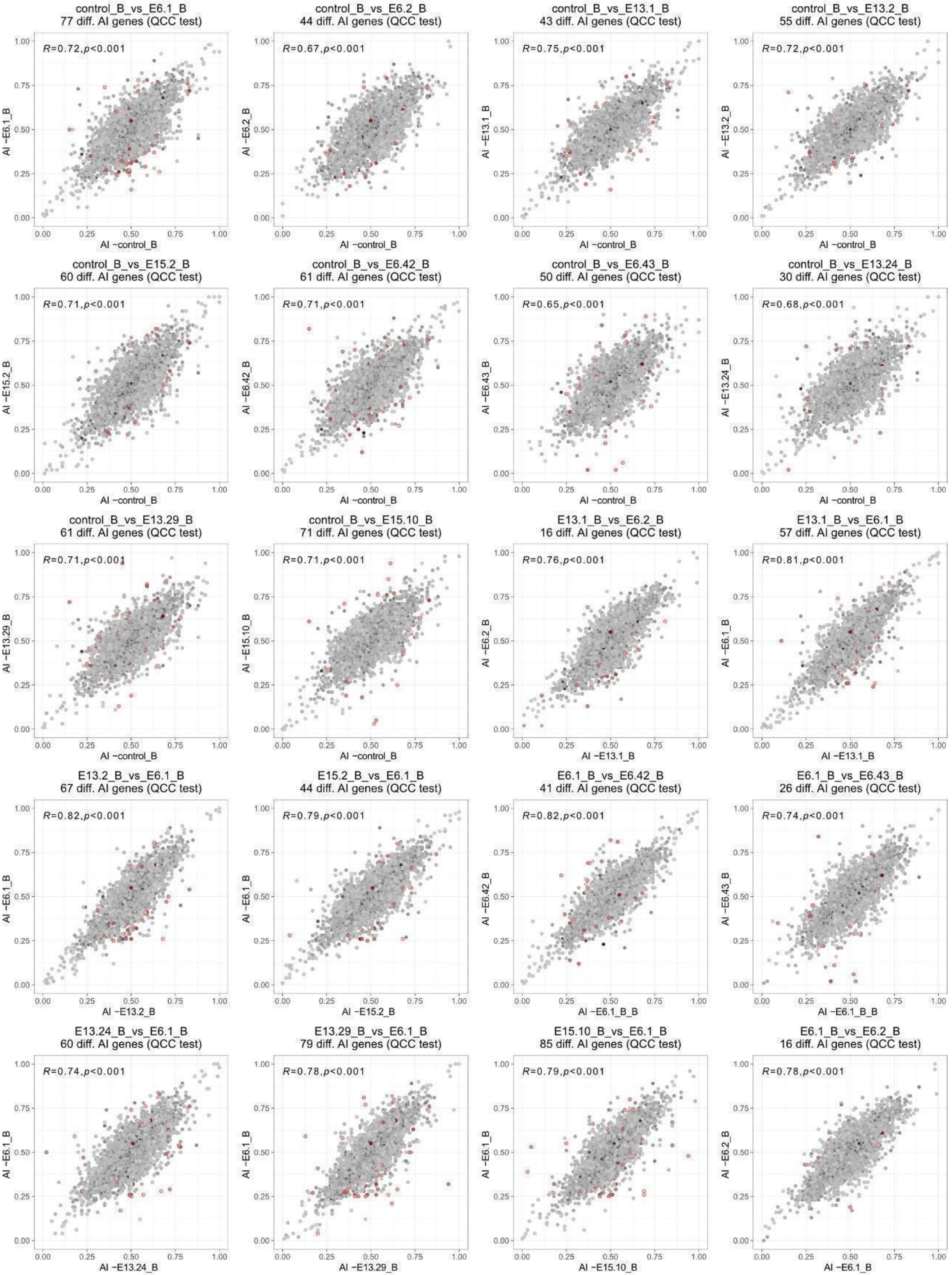
control_B_vs_E6.1_B
77 diff. AI genes (QCC test)
R=0.72,p<0.001
AI −E6.1_B
AI −control_B
control_B_vs_E6.2_B
44 diff. AI genes (QCC test)
R=0.67,p<0.001
AI −E6.2_B
AI −control_B
control_B_vs_E13.1_B
43 diff. AI genes (QCC test)
R=0.75,p<0.001
AI −E13.1_B
AI −control_B
control_B_vs_E13.2_B
55 diff. AI genes (QCC test)
R=0.72,p<0.001
AI −E13.2_B
AI −control_B
control_B_vs_E15.2_B
60 diff. AI genes (QCC test)
R=0.71,p<0.001
AI −E15.2_B
AI −control_B
control_B_vs_E6.42_B
61 diff. AI genes (QCC test)
R=0.71,p<0.001
AI −E6.42_B
AI −control_B
control_B_vs_E6.43_B
50 diff. AI genes (QCC test)
R=0.65,p<0.001
AI −E6.43_B
AI −control_B
control_B_vs_E13.24_B
30 diff. AI genes (QCC test)
R=0.68,p<0.001
AI −E13.24_B
AI −control_B
control_B_vs_E13.29_B
61 diff. AI genes (QCC test)
R=0.71,p<0.001
AI −E13.29_B
AI −control_B
control_B_vs_E15.10_B
71 diff. AI genes (QCC test)
R=0.71,p<0.001
AI −E15.10_B
AI −control_B
E13.1_B_vs_E6.2_B
16 diff. AI genes (QCC test)
R=0.76,p<0.001
AI −E6.2_B
AI −E13.1_B
E13.1_B_vs_E6.1_B
57 diff. AI genes (QCC test)
R=0.81,p<0.001
AI −E6.1_B
AI −E13.1_B
E13.2_B_vs_E6.1_B
67 diff. AI genes (QCC test)
R=0.82,p<0.001
AI −E6.1_B
AI −E13.2_B
E15.2_B_vs_E6.1_B
44 diff. AI genes (QCC test)
R=0.79,p<0.001
AI −E6.1_B
AI −E15.2_B
E6.1_B_vs_E6.42_B
41 diff. AI genes (QCC test)
R=0.82,p<0.001
AI −E6.42_B
AI −E6.1_B_B
E6.1_B_vs_E6.43_B
26 diff. AI genes (QCC test)
R=0.74,p<0.001
AI −E6.43_B
AI −E6.1_B_B
E13.24_B_vs_E6.1_B
60 diff. AI genes (QCC test)
R=0.74,p<0.001
AI −E6.1_B
AI −E13.24_B
E13.29_B_vs_E6.1_B
79 diff. AI genes (QCC test)
R=0.78,p<0.001
AI −E6.1_B
AI −E13.29_B
E15.10_B_vs_E6.1_B
85 diff. AI genes (QCC test)
R=0.79,p<0.001
AI −E6.1_B
AI −E15.10_B
E6.1_B_vs_E6.2_B
16 diff. AI genes (QCC test)
R=0.78,p<0.001
AI −E6.2_B
AI −E6.1_B

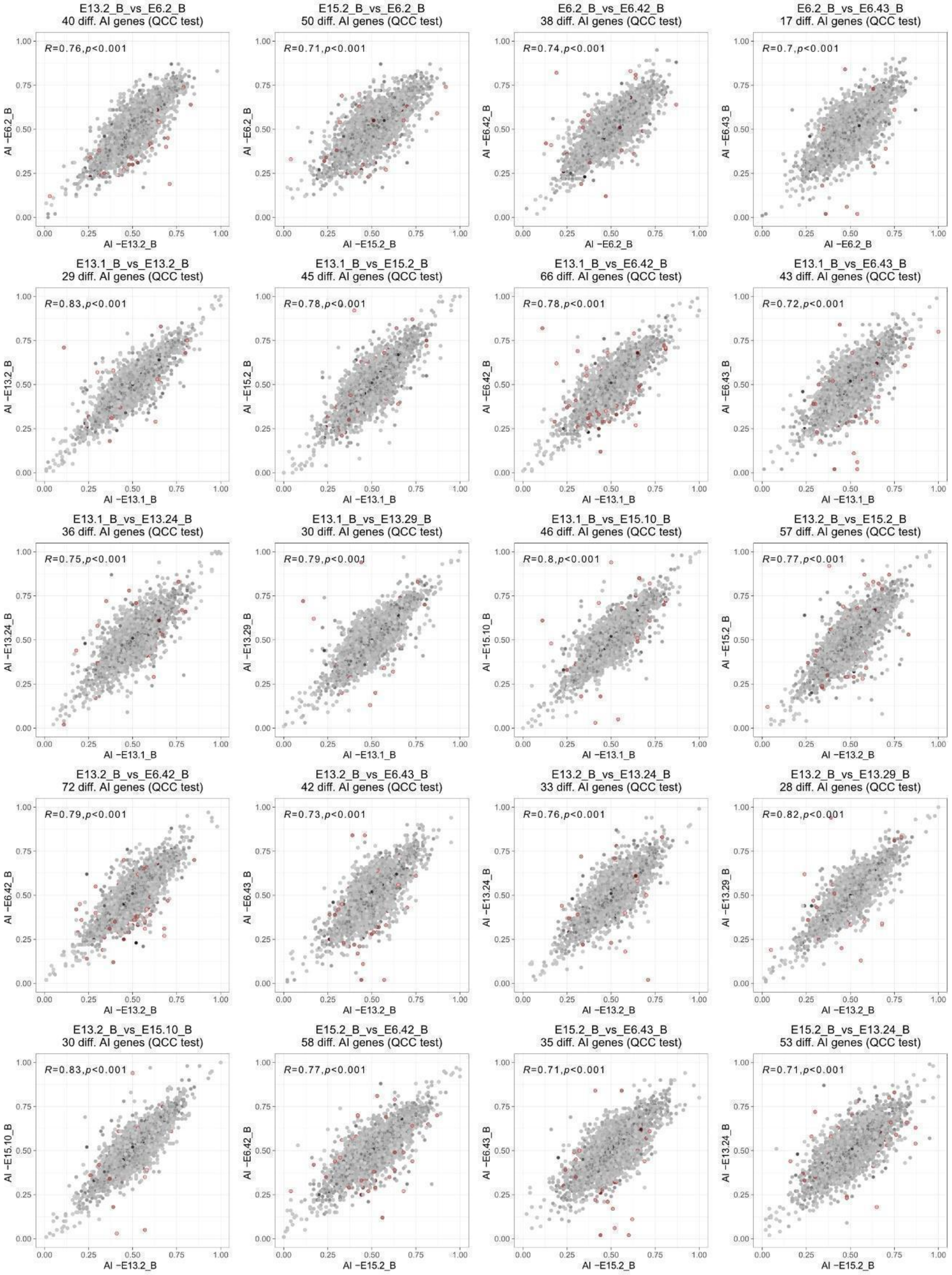
E13.2_B_vs_E6.2_B
40 diff. AI genes (QCC test)
R=0.76,p<0.001
AI −E6.2_B
AI −E13.2_B
E15.2_B_vs_E6.2_B
50 diff. AI genes (QCC test)
R=0.71,p<0.001
AI −E6.2_B
AI −E15.2_B
E6.2_B_vs_E6.42_B
38 diff. AI genes (QCC test)
R=0.74,p<0.001
AI −E6.42_B
AI −E6.2_B
E6.2_B_vs_E6.43_B
17 diff. AI genes (QCC test)
R=0.7,p<0.001
AI −E6.43_B
AI −E6.2_B
E13.1_B_vs_E13.2_B
29 diff. AI genes (QCC test)
R=0.83,p<0.001
AI −E13.2_B
AI −E13.1_B
E13.1_B_vs_E15.2_B
45 diff. AI genes (QCC test)
R=0.78,p<0.001
AI −E15.2_B
AI −E13.1_B
E13.1_B_vs_E6.42_B
66 diff. AI genes (QCC test)
R=0.78,p<0.001
AI −E6.42_B
AI −E13.1_B
E13.1_B_vs_E6.43_B
43 diff. AI genes (QCC test)
R=0.72,p<0.001
AI −E6.43_B
AI −E13.1_B
E13.1_B_vs_E13.24_B
36 diff. AI genes (QCC test)
R=0.75,p<0.001
AI −E13.24_B
AI −E13.1_B
E13.1_B_vs_E13.29_B
30 diff. AI genes (QCC test)
R=0.79,p<0.001
AI −E13.29_B
AI −E13.1_B
E13.1_B_vs_E15.10_B
46 diff. AI genes (QCC test)
R=0.8,p<0.001
AI −E15.10_B
AI −E13.1_B
E13.2_B_vs_E15.2_B
57 diff. AI genes (QCC test)
R=0.77,p<0.001
AI −E15.2_B
AI −E13.2_B
E13.2_B_vs_E6.42_B
72 diff. AI genes (QCC test)
R=0.79,p<0.001
AI −E6.42_B
AI −E13.2_B
E13.2_B_vs_E6.43_B
42 diff. AI genes (QCC test)
R=0.73,p<0.001
AI −E6.43_B
AI −E13.2_B
E13.2_B_vs_E13.24_B
33 diff. AI genes (QCC test)
R=0.76,p<0.001
AI −E13.24_B
AI −E13.2_B
E13.2_B_vs_E13.29_B
28 diff. AI genes (QCC test)
R=0.82,p<0.001
AI −E13.29_B
AI −E13.2_B
E13.2_B_vs_E15.10_B
30 diff. AI genes (QCC test)
R=0.83,p<0.001
AI −E15.10_B
AI −E13.2_B
E15.2_B_vs_E6.42_B
58 diff. AI genes (QCC test)
R=0.77,p<0.001
AI −E6.42_B
AI −E15.2_B
E15.2_B_vs_E6.43_B
35 diff. AI genes (QCC test)
R=0.71,p<0.001
AI −E6.43_B
AI −E15.2_B
E15.2_B_vs_E13.24_B
53 diff. AI genes (QCC test)
R=0.71,p<0.001
AI −E13.24_B
AI −E15.2_B
0.00
0.25
0.50
0.75
1.00

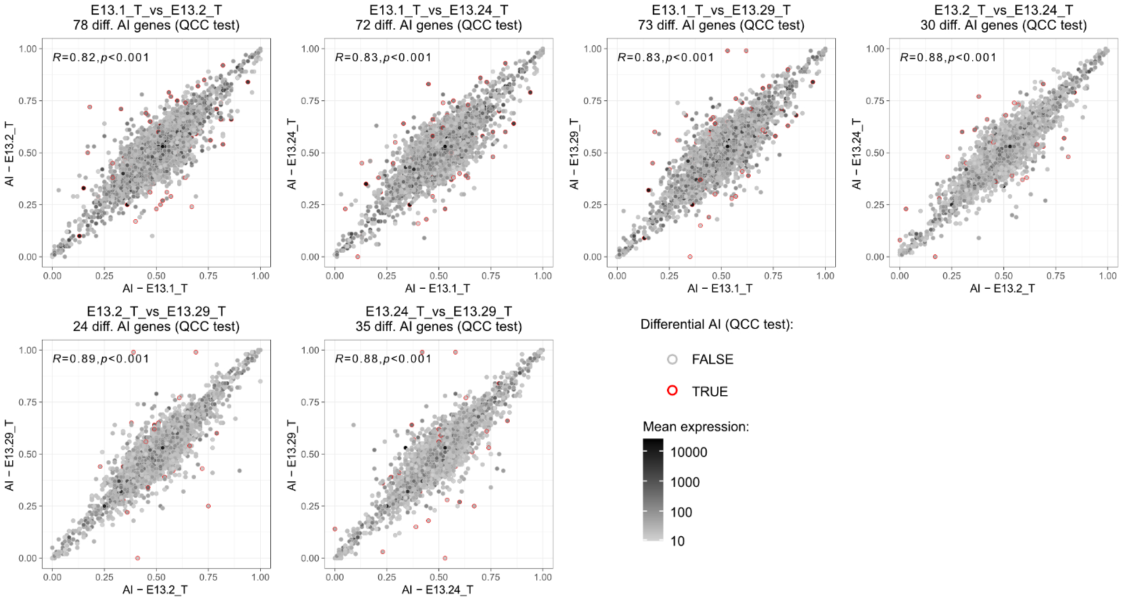

Supplementary Figure 8. Pairwise comparisons of AI between animals for B and T cells, with values of Pearson's coefficient correlation and the number of genes with a significant differential AI after applying QCC correction on the binomial tests. Abundance values are TMM-normalized counts.

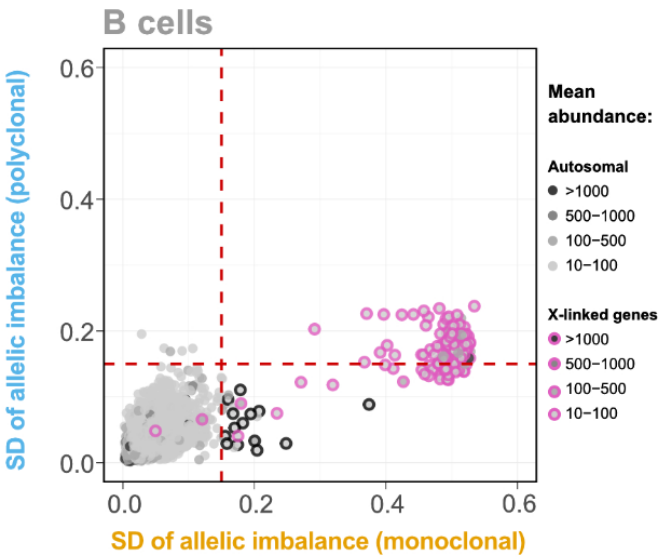

Supplementary Figure 9. Dot plot showing the AI standard deviation (SD) of five B-cell monoclonal samples (*x-axis*) against the AI SD of five polyclonal samples (*y-axis*). Dashed vertical and horizontal lines - arbitrarily set at an AI SD of 0.15 - represent the threshold above which genes were considered as potentially intrinsically imbalanced. Dots represent genes, black-circled dots highlight genes with the highest AI variance among monoclonal samples in the autosomes (from Figure 4A), while pink-circled dots denote the X-linked genes (control). In contrast to Figure 4A, here are shown all genes, and not only those statistically significant after QCC correction in at least one pairwise comparison (see **Figure 3** and **Supplementary Figure 8** - grey genes are included, not only the ones with at least one red dot). As in Figure 4A, only genes expressed in all the 10 B-cell samples were kept, and genes with evidence of nonrandom (genetic or imprinted) AI were excluded.

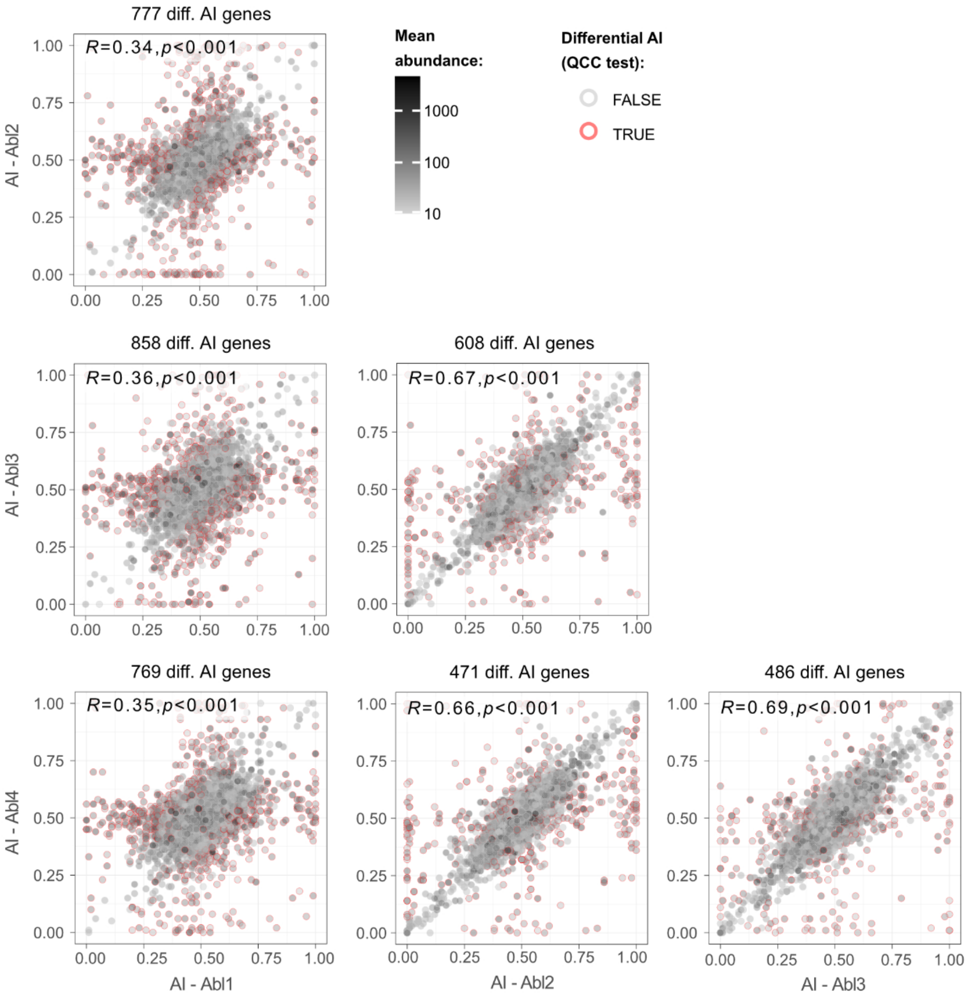

Supplementary Figure 10. Pairwise comparisons of AI between Abelson-immortalized B-cell clones, with values of Pearson's coefficient correlation and the number of genes with a significant differential AI after applying QCC correction on the binomial tests. Abundance values are TMM-normalized counts.

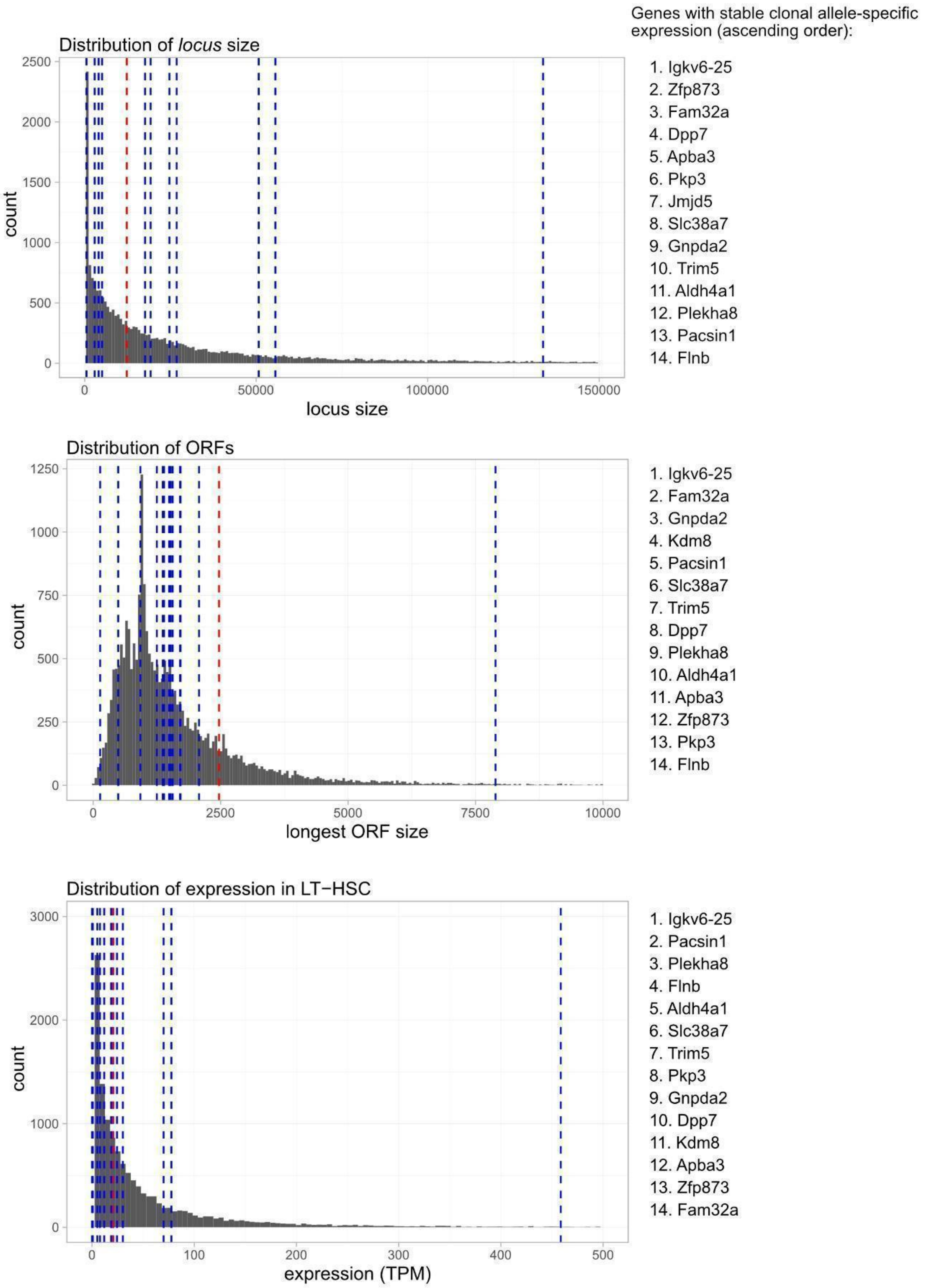

Supplementary Figure 11. Location of 14 genes with persistent clone- and allele-specific autosomal transcriptional states across distributions of *locus* size, open reading frame (ORF) size, and expression in long-term hematopoietic stem cells (LT-HSCs), including all protein-coding genes. Gene sizes were obtained from the latest release of the gencode mouse genome annotations downloaded GTF file (http://ftp.ebi.ac.uk/pub/databases/gencode/Gencode_mouse/release_M27/gencode.vM27.annotation.gtf.gz) with custom scripts. ORFs were generated from the downloaded genecode transcript

sequences fasta file (https://ftp.ebi.ac.uk/pub/databases/gencode/Gencode_mouse/release_M27/gencode.vM27.transcripts.fa.gz) using the orfipy tool (Singh & Wurtele, 2021) with the standard codon table and default parameters. The longest ORF for each gene was plotted for distribution. Expression in LT-HSC was obtained from the Immunological Genome Project (https://www.immgen.org/), GEO:GSE109125. *Locus* and expression plots were zoomed-in for more fitting representation. The blue lines correspond to genes with stable allele-specific transcription through HSC differentiation and the red line corresponds to the gene *Pkp3*.

References

Andergassen, D., Dotter, C. P., Wenzel, D., Sigl, V., Bammer, P. C., Muckenhuber, M., et al. (2017). Mapping the mouse Allelome reveals tissue-specific regulation of allelic expression. eLife, 6, e25125. https://doi.org/10.7554/eLife.25125

Bürglin, T. R., & Affolter, M. (2016). Homeodomain proteins: an update. *Chromosoma*, *125*(3), 497-521. https:// 10.1007/s00412-015-0543-8

Druce, M. (2021). The impact of ageing, replication and stress on genome stability in hematopoietic stem cells. Ph.D. dissertation. Ruperto Carola University, Heidelberg. Germany. https://doi.org/10.11588/heidok.00029501

Gennarino, V. A., D'Angelo, G., Dharmalingam, G., Fernandez, S., Russolillo, G., Sanges, R., et al. (2012). Identification of microRNA-regulated gene networks by expression analysis of target genes. *Genome research*, *22*(6), 1163–1172. https://doi.org/10.1101/gr.130435.111

Hurst, L. D., Sachenkova, O., Daub, C., Forrest, A. R., & Huminiecki, L. (2014). FANTOM consortium. A simple metric of promoter architecture robustly predicts expression breadth of human genes suggesting that most transcription factors are positive regulators. *Genome Biology*, *15*(7), 413. https://10.1186/s13059-014-0413-3

Isakova, A., Fehlmann, T., Keller, A., & Quake, S. R. (2020). A mouse tissue atlas of small noncoding RNA. *Proceedings of the National Academy of Sciences of the United States of America*, *117*(41), 25634–25645. https://doi.org/10.1073/pnas.2002277117

Li, J., Deng, S. P., Vieira, J., Thomas, J., Costa, V., Tseng, C. S., et al. (2018). RBPMetaDB: a comprehensive annotation of mouse RNA-Seq datasets with perturbations of RNA-binding proteins. *Database: the journal of biological databases and curation*, *2018*, bay054. https://doi.org/10.1093/database/bay054

Liu, Y., Pan, C., Kong, D., Luo, J., & Zhang, Z. (2020). A Survey of Regulatory Interactions Among RNA Binding Proteins and MicroRNAs in Cancer. *Frontiers in genetics*, *11*, 515094. https://doi.org/10.3389/fgene.2020.515094

Mendelevich, A., Vinogradova, S., Gupta, S., Mironov, A. A., Sunyaev, S. R., and Gimelbrant, A. A. (2021). Replicate sequencing libraries are important for quantification of allelic imbalance. Nat. Commun. 12, 3370–3382. https://doi.org/10.1038/s41467-021-23544-8.

Ramsköld, D., Wang, E. T., Burge, C. B., & Sandberg R. (2009). An abundance of ubiquitously expressed genes revealed by tissue transcriptome sequence data. *PLoS Computational Biology, 5*(12), e1000598. https://10.1371/journal.pcbi.1000598

Singh, U., & Wurtele, E. S. (2021). orfipy: a fast and flexible tool for extracting ORFs. *Bioinformatics*, *37*(18), 3019–3020. https://doi.org/10.1093/bioinformatics/btab090

Stewart, A. J., Hannenhalli, S., & Plotkin, J. B. (2012). Why transcription factor binding sites are ten nucleotides long. *Genetics*, *192*(3), 973-85. https://10.1534/genetics.112.143370

Tucci, V., Isles, A. R., Kelsey, G., Ferguson-Smith, A. C., Bartolomei, M. S., Benvenisty, N., et al. (2019). Genomic Imprinting and Physiological Processes in Mammals. Cell 176, 952–965. https://doi.org/10.1016/j.cell.2019.01.043.

Zhou, Q., Liu, M., Xia, X., Gong, T., Feng, J., Liu, W., et al. (2017). A mouse tissue transcription factor atlas. *Nature Communications*, *8*, 15089. https://10.1038/ncomms15089

Zhou, Q., Wan, Q., Jiang, Y., Liu, J., Qiang, L., & Sun, L. (2020). A Landscape of Murine Long Non-Coding RNAs Reveals the Leading Transcriptome Alterations in Adipose Tissue during Aging. *Cell reports*, *31*(8), 107694. https://doi.org/10.1016/j.celrep.2020.107694
